# Methane-yielding microbial communities processing lactate-rich substrates: a piece of the anaerobic digestion puzzle

**DOI:** 10.1186/s13068-018-1106-z

**Published:** 2018-04-21

**Authors:** Anna Detman, Damian Mielecki, Łukasz Pleśniak, Michał Bucha, Marek Janiga, Irena Matyasik, Aleksandra Chojnacka, Mariusz-Orion Jędrysek, Mieczysław K. Błaszczyk, Anna Sikora

**Affiliations:** 1 Department of Molecular Biology, Institute of Biochemistry and Biophysics, Polish Academy of Sciences, Warsaw, Poland; 2 Faculty of Earth Sciences, University of Silesia in Katowice, Sosnowiec, Poland; 3 Oil and Gas Institute, National Research Institute, Cracow, Poland; 4 Institute of Geological Sciences, University of Wroclaw, Wroclaw, Poland; 5 Faculty of Agriculture and Biology, Warsaw University of Life Sciences, Warsaw, Poland

**Keywords:** Anaerobic lactate oxidation, Acetogenic step of anaerobic digestion, Wood–Ljungdahl pathway, Microbial communities, 16S rRNA profiling, Genome search

## Abstract

**Background:**

Anaerobic digestion, whose final products are methane and carbon dioxide, ensures energy flow and circulation of matter in ecosystems. This naturally occurring process is used for the production of renewable energy from biomass. Lactate, a common product of acidic fermentation, is a key intermediate in anaerobic digestion of biomass in the environment and biogas plants. Effective utilization of lactate has been observed in many experimental approaches used to study anaerobic digestion. Interestingly, anaerobic lactate oxidation and lactate oxidizers as a physiological group in methane-yielding microbial communities have not received enough attention in the context of the acetogenic step of anaerobic digestion. This study focuses on metabolic transformation of lactate during the acetogenic and methanogenic steps of anaerobic digestion in methane-yielding bioreactors.

**Results:**

Methane-yielding microbial communities instead of pure cultures of acetate producers were used to process artificial lactate-rich media to methane and carbon dioxide in up-flow anaerobic sludge blanket reactors. The media imitated the mixture of acidic products found in anaerobic environments/digesters where lactate fermentation dominates in acidogenesis. Effective utilization of lactate and biogas production was observed. 16S rRNA profiling was used to examine the selected methane-yielding communities. Among *Archaea* present in the bioreactors, the order *Methanosarcinales* predominated. The acetoclastic pathway of methane formation was further confirmed by analysis of the stable carbon isotope composition of methane and carbon dioxide. The domain *Bacteria* was represented by *Bacteroidetes*, *Firmicutes*, *Proteobacteria*, *Synergistetes*, *Actinobacteria*, *Spirochaetes*, *Tenericutes*, *Caldithrix*, *Verrucomicrobia*, *Thermotogae*, *Chloroflexi*, *Nitrospirae,* and *Cyanobacteria.* Available genome sequences of species and/or genera identified in the microbial communities were searched for genes encoding the lactate-oxidizing metabolic machinery homologous to those of *Acetobacterium woodii* and *Desulfovibrio vulgaris*. Furthermore, genes for enzymes of the reductive acetyl-CoA pathway were present in the microbial communities.

**Conclusions:**

The results indicate that lactate is oxidized mainly to acetate during the acetogenic step of AD and this comprises the acetotrophic pathway of methanogenesis. The genes for lactate utilization under anaerobic conditions are widespread in the domain *Bacteria.* Lactate oxidation to the substrates for methanogens is the most energetically attractive process in comparison to butyrate, propionate, or ethanol oxidation.

**Electronic supplementary material:**

The online version of this article (10.1186/s13068-018-1106-z) contains supplementary material, which is available to authorized users.

## Background

Anaerobic digestion is a complex four-step process, promoted by the interaction of many groups of microorganisms, that is comprised of the following steps: (i) hydrolysis of complex organic polymers to monomers; (ii) acidogenesis that results in the formation of hydrogen, carbon dioxide, ammonium, short-chain fatty acids, and alcohols; (iii) acetogenic step that involves the oxidation of non-gaseous fermentation products under anaerobic conditions; and (iv) methanogenesis, which occurs in conditions of low redox potential (< 240 mV) [1–3]. The final two steps, the acetogenic step and the methane formation, are tightly coupled. The acetogenic step, i.e., the oxidation of non-gaseous products of acidogenesis to acetate, hydrogen or formate, and carbon dioxide, is an endergonic process. It involves a reverse electron transfer: the energetically unfavourable movement of electrons that requires the input of energy to drive the oxidation/reduction reaction, as is demonstrated by the positive change in Gibbs free energy. However, when the oxidation processes are coupled to methane production, the conversion becomes thermodynamically favourable [1, 3–6]. The process responsible for energy conservation in syntrophically growing acetate producers is called flavin-based electron bifurcation [7, 8].

The metabolic pathways utilized for syntrophic oxidation of common non-gaseous products of acidogenesis include beta-oxidation for butyrate and the methylmalonyl-CoA pathway for propionate [9]. Worm et al. [10] analyzed the genomes of butyrate- and propionate-oxidizing syntrophs and identified syntrophy-specific functional domains and functional domains involved in electron transfer.

Acetate is a direct substrate for methanogenesis and can also be syntrophically oxidized to hydrogen and carbon dioxide, probably via the oxidative carbon-monoxide dehydrogenase/acetyl-CoA synthase pathway (oxidative CODH/ACS). It is believed that ethanol is oxidized to acetaldehyde coupled to NADH formation. Subsequently, acetaldehyde is oxidized to acetate and reduced ferredoxin is formed. *Pelobacter* species oxidize ethanol in syntrophic cooperation with methanogens [11]. Recently, Bertsch et al. [12] showed that in the acetogen *Acetobacterium woodii* ethanol is converted to acetyl-CoA by the bifunctional ethanol/acetaldehyde dehydrogenase and joining the two activities in one enzyme makes this conversion thermodynamically favoured. Adding the reductive carbon-monoxide dehydrogenase/acetyl-CoA synthase pathway (the Wood–Ljungdahl pathway), *A. woodii* converts ethanol and carbon dioxide to acetate (Δ*G*^0′^ = − 75.4 kJ/mol ethanol).

Surprisingly, until recently, the oxidation of lactate under anaerobic conditions performed by lactate oxidizers did not draw enough attention in the context of the acetogenic step of AD, despite the fact that lactate is a key intermediate in anaerobic digestion of organic matter [13, 14]. Furthermore, lactate oxidation is a thermodynamically attractive process when compared to butyrate, propionate, acetate, or ethanol oxidation. Syntrophic lactate oxidation in the presence of a hydrogenotrophic methane-producing partner has been described for *Desulfovibrio* spp. This occurs only in environments poor in sulfates; otherwise, sulfate reduction occurs. Lactate can also act as a substrate for the non-methanogen *Archaeoglobus*, a known sulfate reducer capable of oxidizing lactate to carbon dioxide [1, 9]. It has also been demonstrated that lactate can be used as a sole carbon source and oxidized to acetate, propionate, and hydrogen by *Megasphaera elsdenii* [15].

Recently, Weghoff et al. [16] described a mode of anaerobic lactate oxidation used by the acetogen *A. woodii*. FAD-dependent lactate dehydrogenase LDH (GlcD domain) in a stable complex with an electron transfer flavoprotein (EtfA/B) catalyzes the following reaction: lactate + Fd^2−^ + 2NAD^+^ → pyruvate + Fd + 2NADH. This process requires reverse electron transport via EtfA/B (electron-bifurcating mechanism). The Etf complex drives endergonic lactate oxidation with NAD^+^ as oxidant at the expense of simultaneous oxidation of reduced ferredoxin. The Rnf complex drives ferredoxin reduction with NADH as reductant.

The Rnf, Ech, or hydrogenase complexes are recognized as functional domains involved in electron transfer in syntrophic bacteria [10], and they have been detected in potential lactate oxidizers [16]. Pyruvate is transformed to acetyl-CoA and further to acetate with the release of ATP. Two molecules of lactate are transformed to two molecules of acetate and two molecules of carbon dioxide. Carbon dioxide is further reduced to acetate via the Wood–Ljungdahl pathway by NADH formed from lactate oxidation. It is important to link the carbon reduction steps with the regeneration of NAD.

Finally, lactate is converted exclusively to acetate (Δ*G*^0′^ = − 61 kJ/mol lactate) and does not require a partner methanogen [17]. It was suggested that this mechanism is utilized by many anaerobic microbes including members of the *Clostridiales*, *Halanaerobiales*, *Fusobacteriales*, *Thermotogales,* and *Thermoanaerobacteriales*, based on the finding that their genomes contain probable operons including the LldP domain (lactate permease), GlcD domain, EtfA and EtfB, and also the LarA domain (lactate racemase) in several cases [16].

In this contribution, we report on an investigation of methane-yielding microbial communities grown on lactate-rich artificial media in up-flow anaerobic sludge blanket (UASB) reactors. These bioreactor communities have been characterized by 16S rRNA profiling. Some of the identified microorganisms had previously sequenced genomes, which we searched to identify genes encoding proteins potentially involved in lactate oxidation and the Wood–Ljungdahl pathway. Lactate was efficiently utilized by the microbial communities. Stable carbon isotopes analysis of methane and carbon dioxide revealed domination of the acetoclastic pathway of methanogenesis. In conclusion, we postulate that when a lactate-rich substrate is processed by a methane-yielding microbial community, the main end product of the acetogenic step is acetate, which is then utilized by acetotrophic methanogens.

## Methods

### Inocula, feed composition, and experimental set-up for processing of lactate-rich media to methane

An experimental set-up for collection of data enabling the description of metabolic transformation of lactate during the acetogenic and methanogenic steps of anaerobic digestion in methane-yielding bioreactors was presented in this study in two repeats. The objects were two methane-yielding microbial communities (designated M1A and M1B) processing a lactate-rich medium in 3.5-L up-flow anaerobic sludge blanket (UASB) bioreactors (Table 1). Both UASB reactors were filled with a methanogenic inoculum (1.5 L) and neutralized lactate-rich artificial medium (2 L), and then incubated at room temperature (20–25 °C). The inocula came originally from the same municipal waste treatment plant. In both cases, the volumes were the same. The lactate-rich artificial medium was a modified M9 medium [18] in which MgSO_4_ was replaced by MgCl_2_ (190 mg/L) and no glucose was added. The medium was supplemented with sodium lactate, butyric acid/sodium butyrate, propionic acid, and acetic acid, as shown in Table 1. Neutralization of the medium with calcium hydroxide (7 g/L) was performed in a separate tank. The medium was supplied to the UASB reactors using a peristaltic pump (ZALIMP, Poland). The M1A bioreactor run lasted for 40 weeks. In the case of M1B, after taking samples for total DNA isolation, the medium flow was switched off for 3 weeks during which the COD of the fluid phase in the bioreactor dropped below 100 mg O_2_/L. Then, the medium with a known carbon isotopic composition was supplied to the bioreactor again and fermentation gas samples were taken for isotope analyses over the next 7 weeks.

**Table 1:** Summary of the experimental set-up used for the processing of a lactate-rich medium to methane

Methane-yielding microbial communities processing lactate-rich medium to methane		
	M1A	M1B
The seed methanogenic inoculum	Activated sludge from a municipal waste treatment plant “Warszawa Południe” in Warsaw, Poland, sampled in the winter^a^	Methane-yielding sludge from the 50-L-UASB bioreactor processing acidic effluent from molasses fermentation [19] inoculated with activated sludge from a municipal waste treatment plant “Warszawa Południe” in Warsaw, Poland, sampled in the autumn^a^
Lactate-rich medium—modified M9 (containing MgCl_2_ instead of MgSO_4_, without glucose)	Sodium lactate 8.26 g/L, butyric acid 1.06 g/L, propionic acid 0.97 g/L, acetic acid 1.54 g/L	Sodium lactate 7 g/L, sodium butyrate 1.3 g/L, propionic acid 0.99 g/L, acetic acid 1.05 g/L, yeast extract 0.5 g/L
Hydraulic retention time (HRT), days	7	7
Culture history	- Incubation at room temperature (20–25 °C) for 26 days after inoculation - 27th–57th day of cultivation—neutralized medium continuously supplied to the bioreactor - Since 58th day of cultivation—non-neutralized medium supplied to the bioreactor	Incubation at room temperature (20–25 °C) for 10 days after inoculation. Since 11th day of cultivation—non-neutralized medium supplied to the bioreactor
Sample collection for 16S rRNA profiling	37th week of cultivation	40th week of cultivation
Analyses performed on the samples collected from the UASB reactors shown in Table 2	33rd—40th week of cultivation	35th—40th week of cultivation
Sample collection for isotope analyses of fermentation gas	–	44th—50th week of cultivation

^a^The director of the Municipal Water and Sewage Enterprise in the capital city of Warsaw in Poland issued the permission to sample activated sludge and use it for scientific research

### Analytical methods

The pH of the medium and the methanogenic effluents as well as the redox potential in the UASB reactor were measured using a standard pH meter (ELMETRON model CP-502) equipped with a combination ORP (redox, mV) electrode-type ERPt-13. The chemical oxygen demand (COD) of the medium and the methanogenic effluents was determined using a NANOCOLOR COD 1500 kit (Macherey-Nagel) according to ISO 1575:2002.

The total rate of gas production was measured using an MGC-1 MilliGascounter (RITTER). The composition of the fermentation gas was analyzed using an HPR20 mass spectrometer (Hiden, England) with QGA version 1.37.

The concentration of short-chain fatty acids in the methanogenic effluents was analyzed by HPLC with photometric detection (Waters HPLC system with Waters 2996—Photodiode Array Detector, using a 300 × 7.8 mm Aminex HPX-87 H column with guard column). The HPLC conditions were as described previously [20].

The concentration of sulfide (S^2−^) in the methanogenic effluents was determined using a NANOCOLOR SULFID 3 kit (Macherey-Nagel) according to the method DIN 38405-D26/27. Effluents were centrifuged before the analyses to remove microbial cells and debris.

Data from all analyses performed on samples collected from the UASB reactors are presented in the respective tables. In each case, the mean values ± SD (standard deviation) are shown.

### Total DNA isolation and 16S rRNA profiling

Total DNA from the methanogenic communities formed in the UASB reactors was isolated from samples taken in the 37th and 40th week, for the M1A and M1B, respectively (Table 1). DNA was extracted and purified using a PowerSoil DNA isolation kit (MoBio Laboratories, Carlsbad, CA) according to the manufacturer’s protocol with some modifications. In each case (M1A and M1B), five 0.3-g samples of the methane-yielding microbial community were placed into five bead tubes for extraction. These tubes were incubated at 65 °C for 20 min and then shaken horizontally in a MoBio vortex adapter for 15 min at maximum speed. The remaining steps were performed as directed by the manufacturer. The final samples of DNA extracted from the five replicates were pooled and stored at − 20 °C. The total masses of purified DNA obtained from the M1A and M1B microbial communities were 25.7 and 20.5 μg, respectively.

Using the total DNA isolated from the methanogenic communities as template, the hypervariable V3–V4 region of the 16S rRNA gene was amplified by PCR. The universal primers 341F and 785R were employed for the simultaneous detection of *Bacteria* and *Archaea* [21]. PCR was performed using Q5 Hot Start High-Fidelity Master Mix (NEB) according to the manufacturer’s instructions. Sequencing of the amplified V3–V4 region libraries was performed using a MiSeq next-generation sequencer (Illumina) with 2 × 250 nt paired-end technology (PE), using the v2 Illumina kit. Automatic analysis of the data to determine the composition of the microbial communities was carried out using 16S Metagenomics software available on the BaseSpace server (Illumina). This analysis consisted of three stages: (i) automatic demultiplexing of the samples, (ii) generation of fastq files containing the raw reads, and (iii) classification of the reads into taxonomic categories.

The 16S Metagenomics Protocol classifies the reads to species level based on the Greengenes v13_5 reference database, modified by Illumina. This modification comprises filtering out the following sequences: (i) of < 1250 base pairs (bp) in length; (ii) containing > 50 degenerate bases (M, R, W, S, Y, K, V, H, D, B, and N); and (iii) those incompletely classified, i.e., not to the level of genus or species. DNA sequencing was performed by the Genomed Joint-Stock Company (Warsaw, Poland).

Two diversity indices were calculated: the Shannon–Wiener index, according to the equation $H^{\prime} = - \mathop \sum \nolimits_{i = 1}^{R} {\text{pi ln pi}}$ (where pi is the proportion of the *i*th element), and true diversity being $^{ 1} D = e^{H'}$ [22, 23].

All raw sequences generated in this study have been deposited in NCBI databases with the following accession numbers: BioProject—PRJNA377904; BioSamples—SAMN06475116 (for M1A) and SAMN06475215 (for M1B); and SRA—SRS2040135 (for M1A) and SRS2040199 (for M1B).

### Intersection analysis of two lactate-processing microbial communities M1A and M1B

For intersection analysis of the two lactate to methane-processing microbial communities, M1A and M1B, only species identified by > 10 reads were selected. Species common to both microbial communities, as well as those present in only one community, were computed and visualized as a Venn diagram using a custom script written in Python 2.7, producing three groups: group I—present only in M1A; group II—present in both M1A and M1B; and group III—present only in M1B.

The distribution of aligned reads between these three groups was visualized in box and violin plots prepared using the ‘vioplot’ package version 0.2 of R 3.2.2 [24].

### Collecting genome sequences for identification of lactate metabolism genes

Genome sequences of species classified in group II (i.e. present in both the M1A and M1B communities) that were available in the NCBI databases were collected. Where only particular genera were identified in the communities, all species of such genera available in the NCBI databases were also collected for inclusion in the analysis. The Assembly database was queried with proper names for identified species or genera. Genome sequences were obtained from the Complete genome, Scaffold and Contig categories within the RefSeq database, and from the GenBank database in the case of *Acidimicrobium* and *Negativicoccus succinicivorans* DORA_17_25. The XML files were downloaded. The ftp addresses for .gbff files were retrieved from these files using a custom R Project script. For every species or genus, a file containing the ftp NCBI addresses for genome sequences was prepared. The .gbff files were downloaded with the wget64 application and imported into Geneious 10.1.2 software (http://www.geneious.com) [25]. The final custom database consisted of 38 items, representing 34 common species and their plasmids, and species for identified genera.

### Searching for genes involved in lactate utilization under anaerobic conditions

The following enzymes involved in anaerobic lactate oxidation were selected as query protein sequences for tBLASTn searches of the prepared custom database (maximal *E*-value of 1e−1, word size set to 3, BLOSUM62 matrix, and gap open/extend cost of 11/1): lactate permease WP_014355268 (AWO_RS04425) (LldP), lactate racemase WP_014355269 (AWO_RS04430) (LarA), electron transfer flavoprotein subunit alpha WP_014355266 (AWO_RS04415) (EtfA), FAD/FMN-containing dehydrogenase WP_014355267 (AWO_RS04420) (GlcD), electron transporter RnfC WP_014356580 (AWO_RS11370) all from *Acetobacterium woodii* DSM 1030 genome NC_016894; l-lactate utilization protein LutB containing Fe–S oxidoreductase WP_028317114 (Q362_RS0100810) from *Desulfobulbus elongatus* DSM 2908 assembly ASM62114v1; [FeFe]-hydrogenase large subunit Fe, Fe_hydrog_A WP_012939287 (ACFER_RS10010) from *Acidaminococcus fermentans* DSM 20731 genome NC_013740; Ni, Fe-hydrogenase III large subunit WP_075074147 (LARV_RS13630) from *Longilinea arvoryzae* strain KOME-1 assembly ASM105023v2; and NADH-quinone oxidoreductase subunit C WP_004312112 (HMPREF1074_RS21770) from *Bacteroides xylanisolvens* CL03T12C04 assembly Bact_xyla_CL03T12C04_V1. BLAST results were always shown with at least 10,000 bp sequence context to permit the examination of neighboring genes. Thus, the NAD-dependent lactate dehydrogenase Ldh_2, Fe–S oxidoreductase GlpC, transcription factor GntR, EtfB, and LutC were identified in the vicinity of the analyzed genes and annotated. The domain/function annotations were assigned according to NCBI Conserved Domains search tool results [26].

### Searching for genes of the Wood–Ljungdahl pathway

Genera present in both the M1A and M1B communities with corresponding read numbers were retrieved (Additional file 1) and used as the input at the Vikodak Local Mapper server [27]. Sample size normalization was used with central tendency set to mean. Different pathways were assayed with the focus on energy metabolism with carbon fixation pathways in prokaryotes. The effective enzyme abundance of the sample is computed as follows, according to [27]:

$$E = \mathop \sum \limits_{i = 1}^{n} Ec_{i}\,x\,Ab_{i} ,$$

where: *E* = effective abundance value of an enzyme; Ec_*i*_ = enzyme copy number value in *i*th taxon of a sample; Ab_*i*_ = 16S and sample size normalized abundance value of *i*th taxon in the given sample; *n* = number of taxa expressing the enzyme in the sample.

The output files representing the effective enzyme abundance profiles were combined in one file (Additional file 2) and EC numbers used as a query in the KEGG Mapper—Search Module [28, 29]. The corresponding functions ascribed to EC numbers identified for the Wood–Ljungdahl pathway were retrieved from the EC database (KEGG) using a custom Python script. All the EC numbers were identified by Vikodak server according to the KEGG database.

### Analyses of stable carbon isotope composition of fermentation gas and substrates

Samples of the fermentation gas were collected from the M1B bioreactor using a sterile syringe and injected into 20-mL glass ampoules with teflon cap filled with a saturated NaCl water solution. The presence of NaCl water solution decreased the solubility of carbon dioxide and consequently allows to avoid the stable carbon isotope fractionation of carbon dioxide while storing. Analyses of stable carbon isotope composition of carbon dioxide and methane were carried out with an on-line method on a Delta V Advantage Mass Spectrometer coupled with a Trace GC Ultra gas chromatograph with a GC Isolink device (Thermo Scientific). The GC column used for gas analyses was an HP-PLOT/Q (Agilent Technologies, dimensions: 30 m × 0.32 mm × 20 µm). Helium was used as the carrier gas. The GC oven was initially held at 30 °C for 4 min, then heated at a rate of 10 °C/min to 210 °C, and held for 4 min. A CO_2_ certified gas standard (δ^13^C_VPDB_ = − 36.2‰, Air Liquide Deutschland, GmbH) was used for calibration. A gas with known carbon isotopic composition was analyzed regularly to check the accuracy of the measurement with ± 0.2‰ precision.

Stable isotope analyses of substrates were carried out using an off-line preparative system. About 2–5 mg or µL of pure substrates were combusted using a CuO wire in a sealed quartz tube, under vacuum at 900 °C [30]. The CO_2_ gas produced was cryogenically purified by off-line technique (liquid nitrogen and dry ice/ethanol mixture). The purified gas was introduced into an isotope ratio mass spectrometer (IRMS; Delta V Advantage/dual inlet, Thermo Scientific) for an analysis of the stable carbon isotope ratio. For the normalization of the δ^13^C values, international standards (NBS22 and USGS24 distributed by the International Atomic Energy Agency, Vienna) were used, and then, the values were reported relative the Vienna Pee Dee Belemnite (VPDB) scale with ± 0.1‰ precision.

## Results

### Performance of the microbial communities processing lactate-rich media

To examine lactate transformation to methane and carbon dioxide, two methane-producing microbial communities continuously processing lactate-rich artificial media in UASB reactors were studied. Composition of the growth media was designed to imitate the acidic products cocktail of microbial communities in environments where lactate fermentation dominates in the acidogenesis.

The data in Table 2 describe the representative performance of the studied methane-yielding microbial communities near the point in time when samples were taken for 16S rRNA profiling. As shown in part A of Table 2, methane was produced by communities M1A and M1B at the respective rates of 18.9 L/L-reactor/day (2.5 L/g COD of the medium) and 25.1 L/L-reactor/day (3.6 L/g COD of the medium). The overall COD removal efficiency was 85 and 97%, for M1A and M1B, respectively. This indicates the efficient utilization of components of the artificial medium by the methane-yielding microbial communities (Table 2, part B). The analysis of short-chain fatty acids revealed almost complete utilization of lactate by both methane-producing communities. Acetate, butyrate, and propionate were also detected in the effluent from the UASB bioreactors.

**Table 2:** Performance of the methane-yielding microbial communities processing a lactate-rich artificial medium

	M1A	M1B
A. Characteristics of the biogas		
Total biogas production		
L/working volume of the bioreactor/day	28.6 ± 0.45	34.9 ± 4.0
Composition of biogas (%)		
Methane	66.0 ± 0.02	71.8 ± 1.2
Carbon dioxide	33.3 ± 0.02	28.1 ± 1.3
Hydrogen	0.66 ± 0.01	0.02 ± 0.03
Hydrogen sulfide	0.01 ± 0.01	0.01 ± 0.01
Methane production		
l-CH_4_/working volume of the bioreactor/day	18.9 ± 0.3	25.10 ± 2.85
l-CH_4_/g COD	2.53 ± 0.04	3.64 ± 0.41

B. Characteristics of the substrate and effluent after the methanogenic process				
	Substrate	Effluent	Substrate	Effluent
COD (g O_2_/L)	15.5 ± 1.2	2.3 ± 0.4	12.5 ± 1.8	0.4 ± 0.1
Concentration of	See Table 1		See Table 1	
Acetic acid (mg/L)		443 ± 236		68 ± 41
Butyric acid (mg/L)		126 ± 43		< 1.0^a^
Lactic acid (mg/L)		22.0 ± 36		4.3 ± 5.8
Propionic acid (mg/L)		518 ± 145		38 ± 22
Sulfide (mg/L)	< 0.05^a^	0.03 ± 0.03	< 0.05^a^	0.09 ± 0.04
pH	5.69 ± 0.17	6.95 ± 0.15	4.91 ± 0.12	7.41 ± 0.05
Redox potential in the UASB bioreactor (mV)^b^		(− 277) to (− 290)		(− 273) to (− 307)

The data come from the analyses done in the 33rd—40th week of cultivation and 35th—40th week of cultivation for M1A and M1B, respectively

^a^The limit of quantification

^b^According to hydrogen electrode

The obtained results for both M1A and M1B show the same tendency of lactate domination as a key factor determining the performance of the bioreactor and the composition of the microbial community as described below.

### Biodiversity of the microbial communities processing artificial lactate-rich medium

The 16S rRNA gene fragment libraries amplified from DNA isolated from the methane-yielding microbial communities have been sequenced. In the case of the community M1A, the total number of reads was 115,296 and 100% of them passed the quality filtering, while for M1B, there were 114,490 reads and 92.8% passed the quality filtering.

Diversity indices were calculated for both analyzed microbial communities. The respective Shannon–Wiener index values for M1A and M1B were 2.79 and 2.09 at the species level, and 3.13 and 3.05 at the genus level. The respective True Diversity index values for M1A and M1B were 16.21 and 8.06 at the species level, and 22.79 and 21.02 at the genus level. These diversity indices for the two communities are comparable and indicate that both are moderately rich in species.

**Fig. 1:**
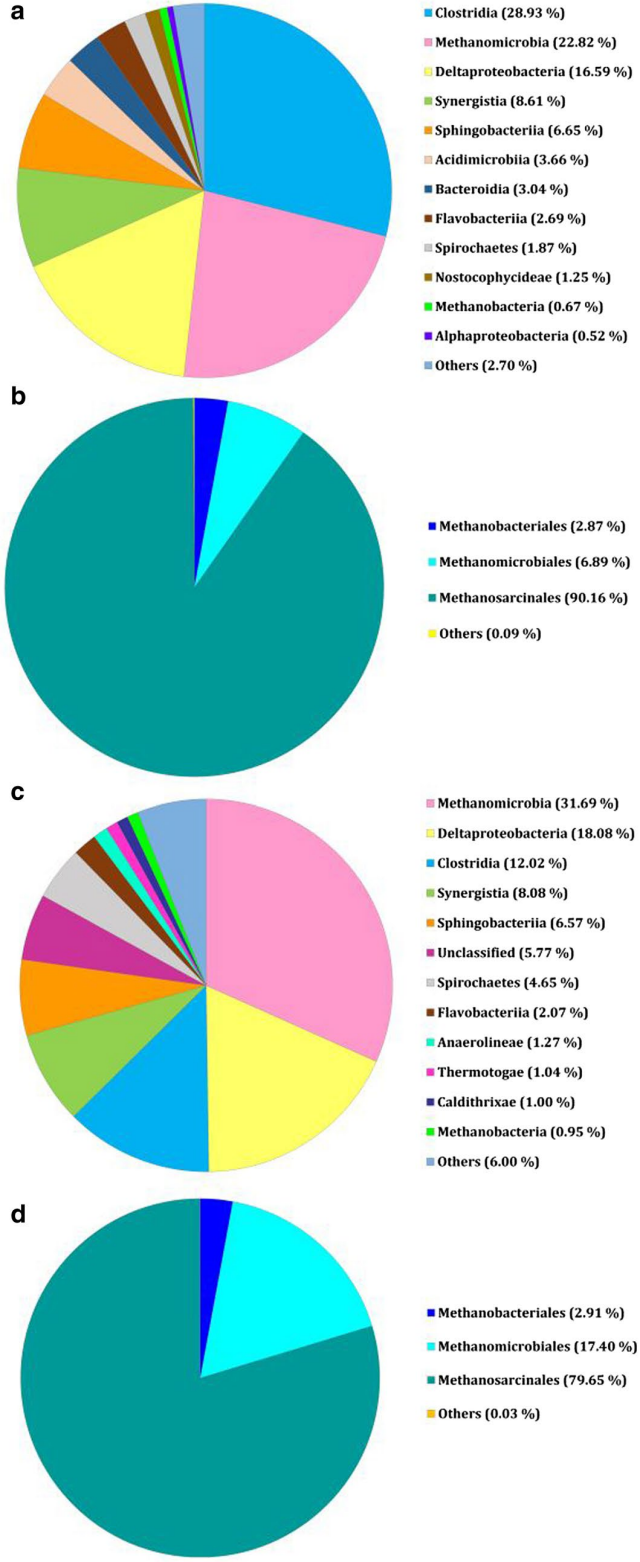
Composition of methane-yielding communities based on the 16S rRNA gene fragment sequences: **a** total microbial community M1A, reads assigned to class level; **b** reads assigned to the *Archaea* domain in community M1A; **c** total microbial community M1B, reads assigned to class level; **d** reads assigned to the *Archaea* domain in community M1B

In the case of M1A, 89,358 reads were assigned to *Bacteria*, 25,744 to *Archaea*, while 194 remained unclassified at the kingdom level, whereas for M1B, these values were 71,102, 34,875, and 236, respectively. For a summary of the taxonomic assignments, see Fig. 1 (detailed assignments are shown in Additional file 3, Additional file 4).

Analysis of the 16S rRNA gene sequences derived from the methanogenic communities formed in the bioreactor revealed that the order *Methanosarcinales* predominated among the *Archaea* (Fig. 1b, d). The most abundant genus within this order was *Methanosaeta*, represented by *M. concilii. Archaea* conducting the hydrogenotrophic pathway of methane production were in the minority, and included *Methanomicrobiales* such as *Methanocorpusculum*, *Methanoculleus*, *Methanospirillum,* and *Methanofollis*.

In the two microbial communities, the domain *Bacteria* was represented by *Bacteroidetes*, *Firmicutes*, *Proteobacteria*, *Synergistetes*, *Actinobacteria*, *Spirochaetes* as well as *Tenericutes*, *Caldithrix*, *Verrucomicrobia*, *Thermotogae*, *Chloroflexi*, *Nitrospirae,* and *Cyanobacteria.* All these phyla are commonly found in anaerobic digesters/biogas plants. The predominant *Bacteroidetes* belonged to the classes *Sphingobacteriia* (order *Sphingobacteriales*, family *Sphingobacteriaceae*), *Flavobacteriia* (order *Flavobacteriales*, family *Flavobacteriaceae*), and *Bacteroidia* (order *Bacteroidiales*, family *Bacteroidaceae* and *Porphyromonadaceae*). The predominant *Firmicutes* belonged to the class *Clostridia*. The order *Clostridiales* was represented by the following families: *Veillonellaceae*, *Syntrophomonadaceae*, *Clostridiaceae*, *Lachnospiraceae,* and *Sulfobacillaceae*. The order *Thermoanaerobacterales* was represented by the *Thermovenabulum* and *Caldicellulosiruptoraceae* families. The predominant *Proteobacteria* belonged to the class *Deltaproteobacteria*. The order *Syntrophobacterales* was represented mainly by *Syntrophaceae* and *Desulfobacteraceae*. The order *Desulfovibrionales* was represented by *Desulfovibrionaceae* and *Desulfohalobiaceae.* The predominant *Synergistetes* belonged to the class *Synergistia* represented by the order *Synergistales*, with the families *Synergistaceae*, *Aminiphilaceae* and *Dethiosulfovibrionaceae*. The phylum *Actinobacteria* was represented by the class *Acidimicrobiia*, order *Acidimicrobiales*, family *Acidimicrobiaceae.* Finally, the phylum *Spirochaetes* was represented by *Spirochaetales* (family *Spirochaetaceae*) and *Sphaerochaetales* (family *Sphaerochaetaceae*).

### Intersection analysis of the microbial communities M1A and M1B

**Fig. 2:**
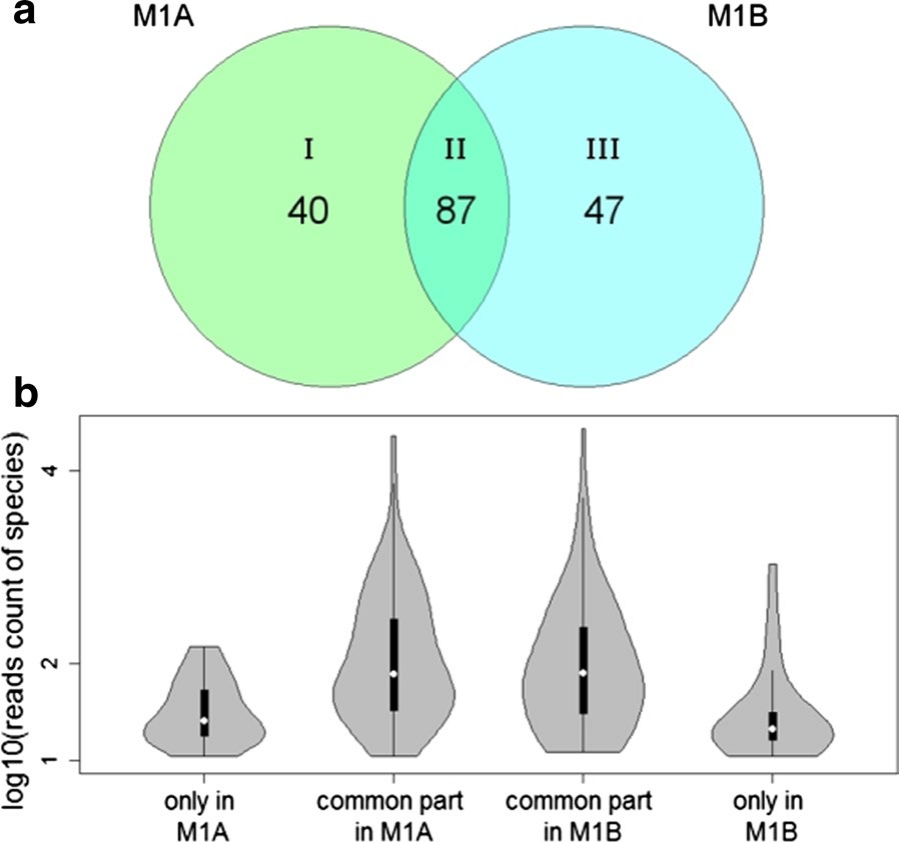
Venn diagram **a** showing the number of species common to both communities, M1A and M1B (group II, 87 species), or characteristic only to M1A (group I, 40 species) or M1B (group III, 47 species). Only species identified by > 10 reads were selected for the analysis. Violin and box plots **b** showing the statistical distribution of read counts for species only present in community M1A (first column), present in both communities (second and third columns), and only present in community M1B (fourth column)

Totally, 127 and 134 species were found in M1A and M1B communities, with more than 10 reads, respectively. Intersection analysis of the two lactate-rich media-processing microbial communities revealed that 87 species were common to both M1A and M1B (designated group II) (Fig. 2a). They constituted 68 and 65% species identified in M1A and M1B, respectively. This common group consisted of species with 580 and 600 mean reads, respectively (corresponding to 79 and 80 median reads). The respective values for species present only in M1A (group I, 40 species) or in M1B (group III, 47 species) were 42 and 75 mean reads (corresponding to 26 and 21 medians). In addition, the read numbers in the common group II showed relatively equal distribution, which contrasts with the species present in groups I and III (Fig. 2b). Consequently, the common species were selected for further genomic analysis, where available.

### The identification of genes potentially responsible for anaerobic lactate oxidation

**Fig. 3:**
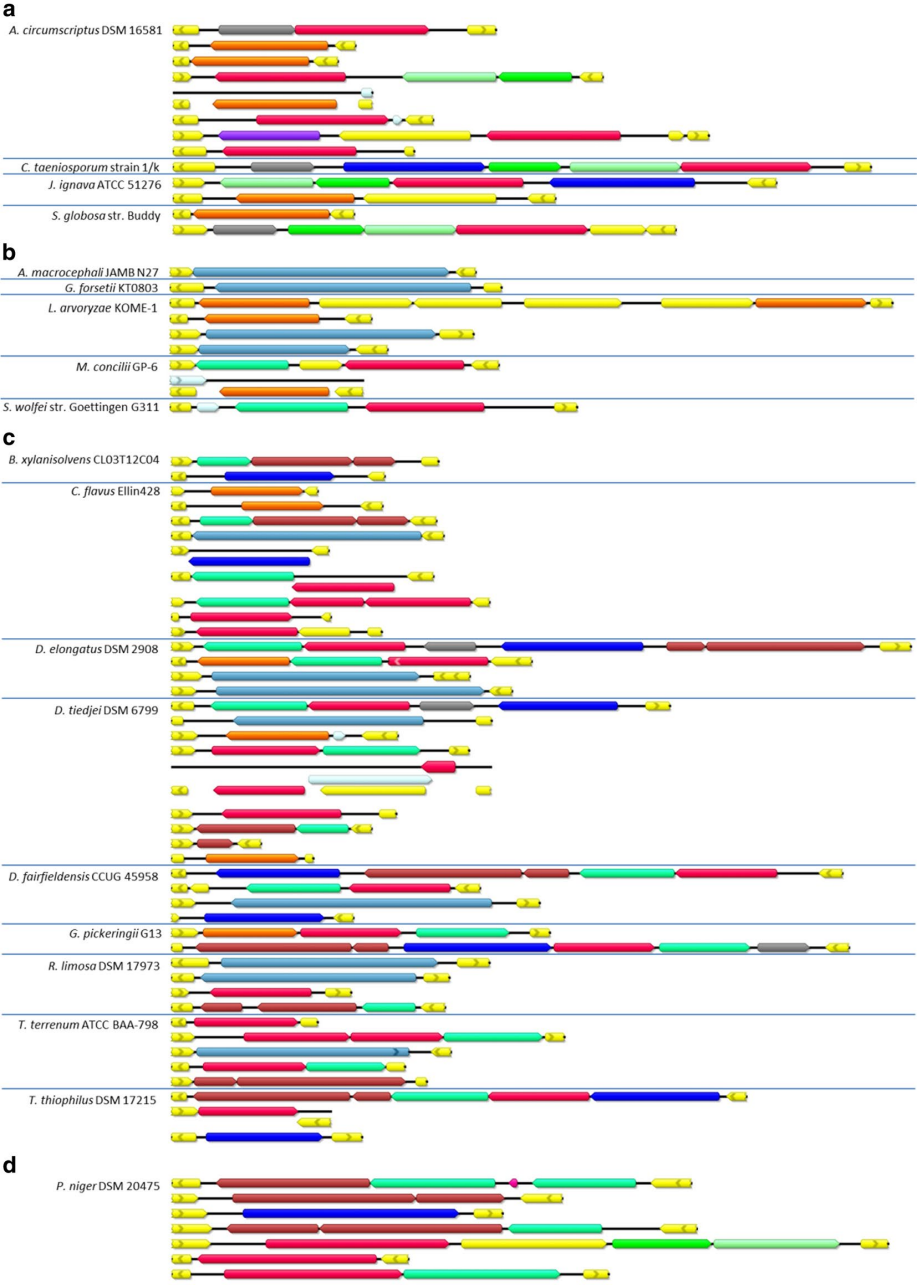
Lactate utilization genes in identified species of known genome sequences with corresponding protein domains: FAD-dependent lactate dehydrogenase (GlcD domain) (purple), (Fe–S)-binding protein GlpC (turquoise), lactate permease (LldP domain) (dark blue), lactate permease (LarA domain) (orange), electron transfer flavoproteins EtfA/B (light/deep green), fused GlcD/GlpC protein (light blue), lactate utilization LutB and LutC proteins (brown), NAD-dependent lactate dehydrogenase Ldh_2 (violet), transcriptional regulator GntR (gray), and other genes (yellow). Groups of genes coding for proteins with the GlcD and EtfA/B exclusively in one operon (**a**), coding the GlcD and GlpC only in a fusion or as probable operon (**b**), or possessing the lut operon and genes encoding GlcD and GlpC (**c**) are shown. *Peptococcus niger* DSM 20475 is shown separately (**d**) as an example of a bacterium possessing genes coding for both GlcD and EtfA/B as well as lut operons, plus additional copies of genes encoding GlcD and GlpC

A database comprised of genome sequences of 34 species classified to group II (i.e., present in both communities, M1A and M1B) was searched for genes encoding proteins potentially involved in anaerobic lactate utilization (Additional file 5 and Fig. 3).

The genomes of *Clostridium taeniosporum* 1/k and *Johnsonella ignava* ATCC51276, both belonging to the *Firmicutes* (*Clostridiales*), and *Sphaerochaeta globosa* str. Buddy (*Spirochaetes*, *Spirochaetales*), contain genes belonging to an operon of *Acetobacter woodii*, previously described by Weghoff et al. [16], although they lack the gene for lactate racemase LarA. In addition, the Rnf complex and at least one Fe-dependent hydrogenase FeFe_hydrog_A are encoded by these genomes, except that of *Clostridium taeniosporum* 1/k. The genome of *Aminiphilus circumscriptus* DSM 16581 (*Synergistetes*, *Synergistales*) contains the operon encoding GlcD and Ldh_2, EtfA/B, and three LarA proteins, FeFe_hydrog_A and Ni, Fe-hydrogenase III large subunits, but it lacks the gene for lactate permease LldP. Genomes of two *Acidaminococcales* (*Firmicutes*) species, *Acidaminococcus fermentans* DSM 20731 and *Phascolarctobacterium succinatutens* YIT 12067, encode at least 3 GlcD proteins and at least one EtfA/B complex, but no lactate permease genes are present. In addition, they encode at least two FeFe_hydrog_A proteins. One of the LarA genes of *P. succinatutens* YIT 12067 is present in an operon encoding an NADH-dependent HicDH-like protein with C-terminal domain of lactate/malate dehydrogenases Ldh_1_C capable of converting l-lactate to pyruvate. The bacterial genomes encoding the GlcD domain and the EtfA/B in one operon are shown in Fig. 3a.

In several species, like *Gramella forsetii* KT0802 (*Flavobacteriales*, *Bacteroidetes*), *Longilinea arvoryzae* KOME-1 (*Chloroflexi*, *Anaerolineales*), *Syntrophomonas palmitatica* JCM 14374, *S. wolfei* subsp. *methylbutyrica,* and *S. wolfei* subsp. *wolfei* str. Goettingen G311 *(Firmicutes*, *Clostridiales*), the gene encoding the EtfA/B complex is not located in an operon with the GlcD gene (Fig. 3b). The GlcD domain is either fused to or adjacent to Fe-S oxidoreductase GlpC. The two *S. wolfei* subspecies *methylbutyrica* and *wolfei* str. Goettingen G311 also possess genes for at least 3 Ni-dependent hydrogenases containing Ni, Fe-hydrogenase III large subunit or FeFe_hydrog_A proteins, respectively. *S. palmitatica* JCM 14374 also possesses genes encoding an Rnf complex, and *S. wolfei* subsp. *methylbutyrica*, a Ni, Fe-hydrogenase I large subunit. Similarly, *Aquimarina macrocephali* JAMB N27 (*Bacteroidetes*, *Flavobacteriales*) encodes a GlcD/GlpC fusion and EtfA/B complex, but no LldP or LarA genes were found. All these bacterial species apparently lack a lactate permease (LldP).

Interestingly, the genome of euryarchaeotan acetotrophic methanogen *Methanosaeta concilii* GP-6 contains an operon coding for GlcD, GlpC and LarA; however, it too lacks a lactate permease gene (Fig. 3b). The same genes were identified in *M. harundinacea* 6Ac. *M. pelagica* was also detected in the studied microbial communities, but its genome sequence is not available.

Among the bacteria growing on lactate in the bioreactor, many species were found to possess genes coding for LutC and LutB proteins (Fig. 3c). The genome of *Bacteroides xylanisolvens* CL03T12C04 (*Bacteroidetes*, *Bacteroidales*) contains an operon composed of genes for C-GlpC, LutB, and LutC, and an LldP gene in different parts of the genome. It also encodes an Rnf complex and FeFe_hydrog_A. Similarly, the genome of *Runella limosa* DSM 17973 (*Bacteroidetes*, *Cytophagales*) contains two gene fusions encoding GlcD/GlpC and one operon comprised of the C-GlpC, LutB, and LutC genes, plus genes coding for GlcD and EtfA/B spread throughout the genome. One gene encoding a Ni, Fe-hydrogenase I large subunit is also present. However, *R. limosa* DSM 17973 lacks a lactate permease gene. An even more complex machinery of lactate utilization is encoded by the genomes of *Chthoniobacter flavus* Ellin428 (*Verrucomicrobia*, *Chthoniobacterales*) and *Thermobaculum terrenum* ATCC BAA-798 (unclassified *Terrabacteria* group). In the *Chthoniobacter* species, genes coding for the following proteins were found: lactate permease LldP, two copies of LarA, at least three GlcD copies, two of which are in an operon with GlpC, with one containing an additional GlcD protein, a GlcD/GlpC fusion (with a DUF3390 domain), plus GlpC, LutB, and LutC genes in an operon. The genes for lactate utilization under anaerobic conditions are similar in the genome of the *Thermobaculum* species except that the encoded LutB protein is in a fusion with GlpC, and lactate permease LldP and LarA genes are not present.

The most diverse collections of lactate utilization genes were found in the representatives of the *δ*-*Proteobacteria* such as the sulfate-reducing bacteria: *Desulfobulbus elongatus* DSM 2908 (*Desulfobacterales*), *Desulfomonile tiedjei* DSM 6799 (*Syntrophobacterales*), *Desulfovibrio fairfieldensis* CCUG 45958 (*Desulfovibrionales*), as well as *Geobacter pickeringii* G13 (*Desulfuromonadales*) and *Thermodesulfovibrio thiphilus* DSM 17215 (*Nitrospirae*, *Nitrospirales*) (Fig. 3c). All except *D. tiedjei* DSM 6799 contain an operon encoding LldP, GlcD, GlpC, LutC, and a LutB/GlpC fusion. In addition, the genomes of the first three species code for at least one GlcD/GlpC fusion, and all apart from *T. thiphilus* DSM 17215 possess an additional operon composed of genes encoding GlcD and GlpC, and possibly LarA. All species except *D. fairfieldensis* CCUG 45958 code for an EtfA/B complex, but not in the vicinity of the aforementioned genes. The LldP, GlcD, and GlpC operon, and other genes of anaerobic lactate utilization, although dispersed in the genome are present in *D. tiedjei* DSM 6799. All the species code for at least one Ni, Fe-hydrogenase III large subunit and, with the exception of *D. elongates* DSM 2908, a Ni, Fe-hydrogenase I large subunit, as well. The first three species also possess genes for Fe, Fe_hydrog_A, and Rnf complex genes are present in the genomes of *D. tiedjei* DSM 6799 and *D. fairfieldensis* CCUG 45958.

*Peptococcus niger* DSM 20475 (*Firmicutes*, *Clostridiales*) seems to combine two pathways of lactate utilization under anaerobic conditions. The operon comprised of GlcD and EtfA/B genes is present, and it also possesses a C-GlpC, LutB and LutC operon. In addition, it has a GlcD/GlpC fusion gene, an *N*-GlpC, C-GlpC, and LutB operon, an additional copy of the LutB gene, and two additional copies of genes encoding EtfA/B. Lactate permease LldP and Fe, Fe_hydrog_A genes are also present (Fig. 3d).

The *Aminobacterium colombiense* DSM 12261 (*Synergistetes*, *Synergistales*) genome codes only for one LldP and three LarA proteins, with one of the latter genes probably in an operon with NADH-dependent l-lactate dehydrogenase Ldh_2, capable of oxidizing lactate to pyruvate. There are also genes encoding Rnf and two Ni, Fe-hydrogenase III large subunits.

The genomes of *Bellilinea caldifistulae* GOMI-1 (*Chloroflexi*, *Anaerolineales*), *Caloramator mitchellensis* VF08 (*Firmicutes*, *Clostridiales),* and *Negativicoccus succinicivorans* DORA_17_25 (*Firmicutes*, *Veillonellales*) lack genes for FAD-dependent dehydrogenase, but all encode an EtfA/B complex. In addition, *B. caldifistulae* GOMI-1 possesses genes coding for two LldP proteins, one LarA, and one Ni, Fe-hydrogenase III large subunit; *C. mitchellensis* VF08 has three FeFe_hydrog_A genes and one Ni, Fe-hydrogenase I large subunit gene; and *N. succinicivorans* DORA_17_25 has a LarA gene. *Peptonophilus coxii* DNF00729 (*Firmicutes*, *Tissierellales*) lacks genes encoding FAD-dependent lactate dehydrogenases, LldP, and the Lut operon, but it contains at least two EtfA/B complex genes, one of which is in the vicinity of the genes encoding HicDH-like dehydrogenase, Rnf, and the hydrogenase FeFe_hydrog_B1.

Several species common to the microbial communities M1A and M1B lack complete or scaffold genome sequences. In addition, many reads in these communities were identified only at the genus level. To further elucidate the likely nature of the lactate metabolism in microorganisms present in the bioreactor, we also searched for relevant genes in the genomes of species related to those identified in the microbial communities. A detailed analysis is presented in Additional file 5.

### Searching for Wood–Ljungdahl pathway genes

**Fig. 4:**
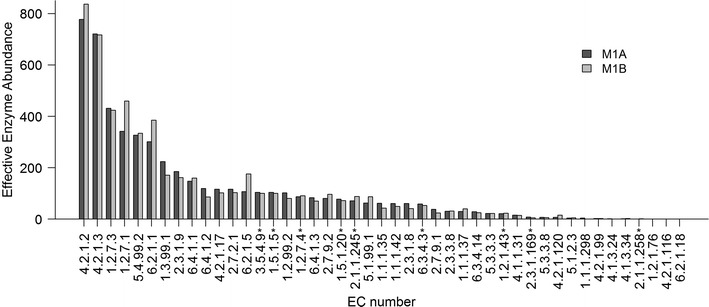
Relative abundance of enzymes, identified by EC number, (for details, see “Methods”) of prokaryotic carbon fixation pathways in bioreactor microbial communities M1A and M1B. Enzymes comprising the Wood–Ljungdahl pathway are marked with an asterisk. EC 4.2.1.2—fumarase; EC 4.2.1.3—aconitase; EC 1.2.7.3 2—oxoglutarate synthase; EC 1.2.7.1—pyruvate synthase; EC 5.4.99.2—methylmalonyl-CoA mutase; EC 6.2.1.1—acetate-CoA ligase; EC 1.3.99.1—succinate dehydrogenase; EC 2.3.1.9—acetyl-CoA C-acetyltransferase; EC 6.4.1.1—pyruvate carboxylase; EC 6.4.1.2—acetyl-CoA carboxylase; EC 4.2.1.17—enoyl-CoA hydratase; EC 2.7.2.1—acetate kinase; EC 6.2.1.5—succinate-CoA ligase (ADP-forming); EC 3.5.4.9—methenyltetrahydrofolate cyclohydrolase; EC 1.5.1.5—methylenetetrahydrofolate dehydrogenase (NADP^+^); EC 1.2.99.2—carbon-monoxide dehydrogenase (acceptor); EC 1.2.7.4—anaerobic carbon-monoxide dehydrogenase; EC 6.4.1.3—propionyl-CoA carboxylase; EC 2.7.9.2—phosphoenolpyruvate synthase; EC 1.5.1.20—methylenetetrahydrofolate reductase [NAD(P)H]; EC 2.1.1.245—5-methyltetrahydrosarcinapterin:corrinoid/iron-sulfur protein Co-methyltransferase; EC 5.1.99.1—methylmalonyl-CoA racemase; EC 1.1.1.35—3-hydroxyacyl-CoA dehydrogenase; EC 1.1.1.42—isocitrate dehydrogenase (NADP^+^); EC 2.3.1.8—phosphate acetyltransferase; phosphotransacetylase; EC 6.3.4.3—formate-tetrahydrofolate ligase; EC 2.7.9.1—pyruvate-phosphate dikinase; EC 2.3.3.8—ATP citrate synthase; EC 1.1.1.37—malate dehydrogenase; EC 6.3.4.14—biotin carboxylase; EC 1.2.1.43—formate dehydrogenase (NADP^+^); EC 4.1.1.31—phosphoenolpyruvate carboxylase; EC 2.3.1.169—CO-methylating acetyl-CoA synthase; EC 5.3.3.8—dodecenoyl-CoA isomerase; EC 4.2.1.120—4-hydroxybutanoyl-CoA dehydratase; EC 5.1.2.3—3-hydroxybutyryl-CoA epimerase; EC 1.1.1.298—3-hydroxypropionate dehydrogenase (NADP^+^); EC 4.2.1.99—2-methylisocitrate dehydratase; EC 4.1.3.24—malyl-CoA lyase; EC 4.1.3.34—citryl-CoA lyase; EC 2.1.1.258—5-methyltetrahydrofolate:corrinoid/iron-sulfur protein Co-methyltransferase; EC 1.2.1.76—succinate-semialdehyde dehydrogenase (acylating); EC 4.2.1.116—3-hydroxypropionyl-CoA dehydratase; EC 6.2.1.18—citrate-CoA ligase

Genes encoding all nine proteins of the reductive acetyl-CoA (Wood–Ljungdahl) pathway (KEGG Module: M00377) (Additional file 6) were found in both microbial communities, M1A and M1B, with similar frequency (Additional file 2). The gene for carbon-monoxide dehydrogenase (catalytic subunit) (EC: 1.2.7.4), the first enzyme in the pathway, occurs with an abundance of 87–91 (Fig. 4). Genes for the other proteins are present with frequencies ranging from 0.4 to 104 (Fig. 4).

### Stable carbon isotope composition of substrates and fermentation gas

**Fig. 5:**
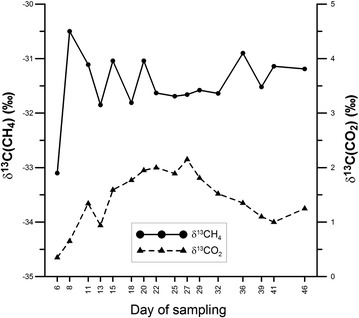
Variation of δ^13^CO_2_ (‰) and δ^13^CH_4_ (‰) in time

Fermentation substrates (yeast extract, sodium lactate, sodium butyrate, propionic acid, and acetic acid) as well methane and carbon dioxide in 16 fermentation gas samples were analyzed to determine their stable carbon isotopic composition. All the results are presented in the Additional file 7 and on Fig. 5. The δ^13^C values of fermentation substrates ranged from − 44.9 to − 23.0‰. The results of stable carbon isotope analyses in carbon dioxide varied from 0.4 to 2.2‰ with an average of 1.4‰. The results of stable carbon isotope analyses in methane ranged from − 33.1 to − 31.0‰ with an average of − 31.5‰. It is worth mentioning that the lowest values of both δ^13^C(CO_2_) and δ^13^C(CH_4_) were noted for the same, first gas sample.

According to Jędrysek [31] and Vinson et al. [32] in a regular, closed methanogenic system, CH_4_ is depleted of ^13^C compared to the source of organic carbon, whereas the CO_2_ is enriched in ^13^C compared to the source of organic carbon. In that experiment, the dominant substrate was the sodium lactate, for which the δ^13^C value is − 23.0‰. The δ^13^C(CO_2_) and δ^13^C(CH_4_) values obtained during the experiment are − 31.5 and 1.4‰, respectively. This is in accordance with the literature predictions.

The dominant pathway of methanogenesis can be established by means of stable carbon isotopic composition of methane and the difference between δ^13^C(CO_2_) and δ^13^C(CH_4_). According to Sugimoto and Wada [33], the values of δ^13^C(CH_4_) more positive than − 33.0‰ indicate that the acetoclastic pathway is the dominant pathway during the methanogenesis. The δ^13^C(CH_4_) values lower than − 60.0‰ are indicative of a purely hydrogenotrophic pathway. The results of δ^13^C(CH_4_) in this work have an average value of − 31.5‰. The isotope fractionation factor between carbon dioxide and methane [α(CO_2_–CH_4_)] ranged from 1.032 to 1.035 with an average of 1.034. These results come close with the range for the acetoclastic methanogenesis (1.039–1.058) estimated by Whiticar [34] whereas α(CO_2_–CH_4_) values of 1.049–1.095 are characteristics of the hydrogenotrophic methanogenesis. The α(CO_2_–CH_4_) as low as 1.034 is an evidence that in the experiment only the acetoclastic pathway took place. The lower limit of the range estimated by Whiticar [34] for such a fermentation scenario could, perhaps, be shifted from 1.039 down to 1.034.

This is in agreement with the fact that methanogenesis with the dominant acetoclastic pathway experiences mutual positive or negative variations in both δ^13^C(CO_2_) and δ^13^C(CH_4_). Figure 5 shows shifts in both δ^13^C(CO_2_) and δ^13^C(CH_4_) in the same direction, especially on the 11–13, 13–15, and 36−39 days of sampling. Note that the days of sampling are numbered from the beginning of the 44th week of cultivation, i.e., the 6th day of sampling belongs to the 44th week of cultivation, etc. On the other hand, in methanogenesis with the dominant hydrogenotrophic pathway, an opposite relationship is observed (increasing amounts of stable carbon isotopes in carbon dioxide is accompanied by decreasing amounts of stable carbon isotopes in methane). Such CO_2_–CH_4_ isotopic picture has been described for numerous natural conditions [31, 35]. Our other studies (data not shown) with a replicate of the M1A culture also indicated the acetoclastic pathway of methane synthesis.

The performance of the M1B methane-yielding microbial community at the time samples of the fermentation gas were taken for analysis of the stable carbon isotope composition (Additional file 8) was comparable to that presented in Table 2.

## Discussion

### Lactate as a key intermediate in anaerobic digestion and a factor that determines the type of methanogenic pathway

Lactate, a product of acidic fermentation, is an important intermediate in anaerobic digestion of organic matter. Lactic acid bacteria are widespread and universal microbes in terrestrial and aquatic environments. They are found in plants and animals, where they constitute a significant component of the microbial flora of the gastrointestinal and genitourinary tracts as well as skin and mucosa. Lactic acid bacteria are responsible for food fermentation and this process has been used to preserve some foodstuffs [13, 36].

Various types of silage are common feedstocks for commercial biogas plants. Silage technology has been applied to biomass storage in biogas production and ensilaging is regarded as a means of increasing methane yield from anaerobic digestion. Lactic acid is a common product of the acidogenic step in two-stage anaerobic digesters [14, 37–40].

Many studies clearly show that lactate is effectively utilized by methane-producing microbial communities. Analyses of the acidogenic fraction subjected to methanogenesis and the end products of the process in two-stage biogas digesters showed that lactate is the best utilized component, irrespective of its initial concentration [38, 41–43].

In this view, a relatively small number of studies have been published on anaerobic lactate oxidation (in comparison to butyrate oxidation and propionate oxidation) during the acetogenic step of AD. Studies done on pure cultures of *Acetobacter woodii* and *Desulfovibrio vulgaris* have constituted milestones in the research on the anaerobic lactate oxidation. However, they show the metabolism of single species only and not the functioning of whole microbial communities.

Considering the above issues, in the present study, methane-yielding microbial communities instead of pure cultures of microorganisms were used to process lactate-rich artificial media to collect data allowing the description of the metabolic transformation of lactate during the acetogenic and methanogenic steps of AD in methane-yielding bioreactors. The artificial media were intended to imitate a mixture of acidic products in the anaerobic environments/anaerobic digesters where lactate fermentation dominates. Both the acetogenic and methanogenic steps took place in the bioreactors and the effective utilization of lactate was observed. Our system is closer to the natural environments and biogas digesters where microbial communities and not pure cultures exist.

**Fig. 6:**
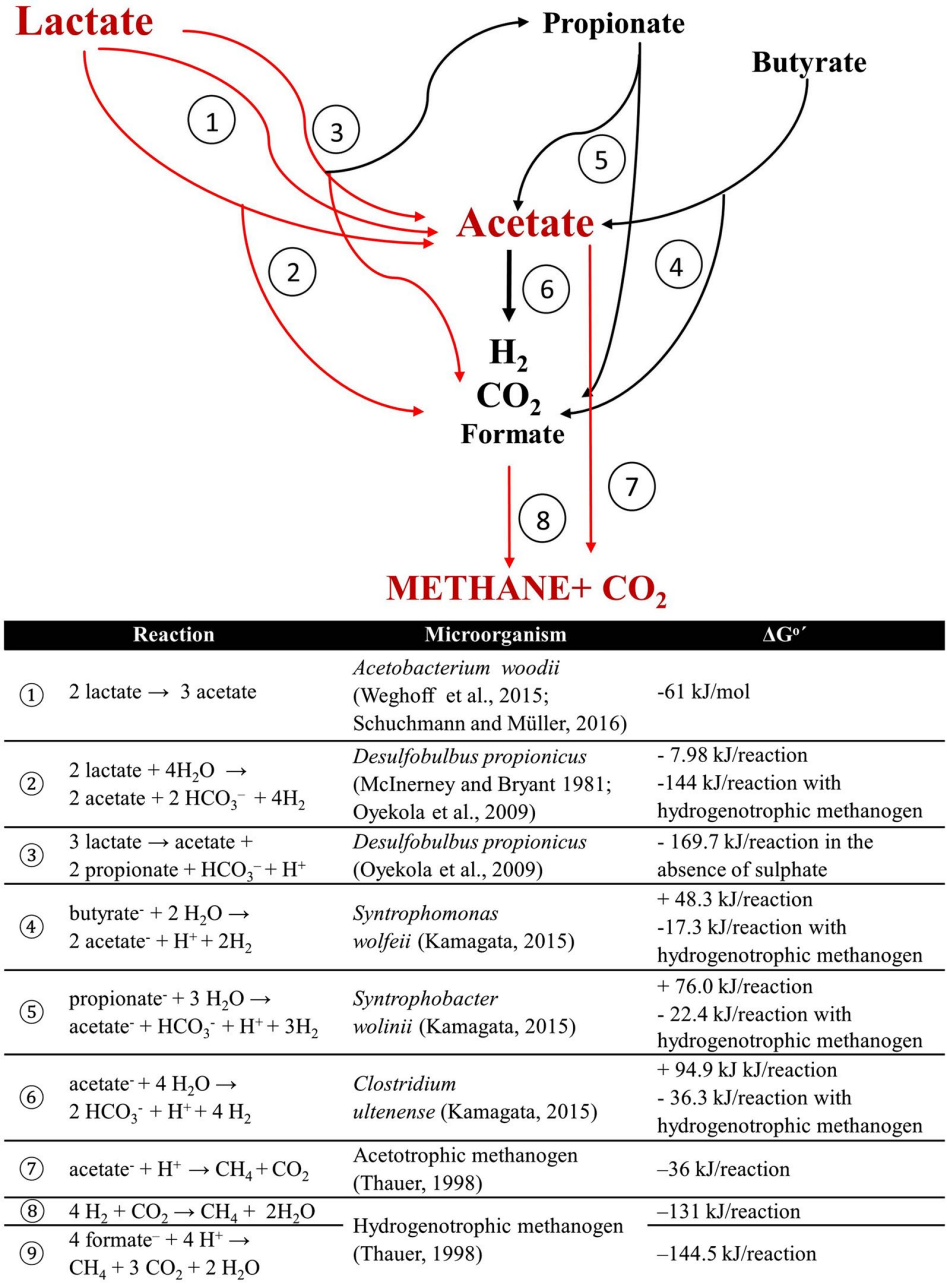
Processing of lactate-rich media to methane and carbon dioxide by microbial communities in the UASB bioreactor (acetogenic and methanogenic steps)

The respective ΔG^0′^ values/reaction for the oxidation of acetate, butyrate, propionate, ethanol, and lactate clearly shows that lactate degradation requires the lowest energy input and provides the highest energy gain for acetic acid-producing bacteria [4, 5, 44, 45]. This determines the attractiveness of lactate as an intermediate during anaerobic digestion (Fig. 6). Furthermore, the contribution of hydrogenotrophic methanogens to the process is not required [16, 17]. Anaerobic oxidation of lactate to acetate creates excellent selective conditions for acetotrophic methanogens.

Culture-independent molecular analyses of methanogenic communities based on next-generation sequencing have revealed that the contribution of methanogens performing the acetoclastic, methanotrophic, or hydrogenotrophic pathways in anaerobic digesters depends on the nature of the substrate and the process conditions [42, 46–49]. We previously discussed this issue and presented arguments in favor of the dominance of the hydrogenotrophic pathway of methane generation in bioreactors processing the acidic effluent from molasses fermentation [3, 42].

Our data on the processing of lactate-rich medium by the microbial communities in the bioreactor clearly show that the acetoclastic pathway of methanogenesis is dominant and further support the thesis that the substrate determines the operative methanogenic pathway.

As it has been shown above, stable carbon isotope analysis of carbon dioxide and methane in the fermentation gas points to the acetoclastic pathway of methanogenesis in the bioreactor. It is convincing evidence that shows the enzymatic activity in the microbial community. It is worth adding that the isotope distribution factor between carbon dioxide and methane stays within the range for hydrogenotrophic methanogenesis when butyrate or propionate-rich media are processed to methane (unpublished data).

The most abundant methanogens were *Methanosarcinales* represented by *Methanosaeta. Methanosarcina* and *Methanosaeta*, members of the order *Methanosarcinales*, are capable of methane production from acetate. Moreover, only *Methanosaeta* is strictly acetoclastic, whereas *Methanosarcina* is able to produce methane from acetate, CO_2_ and H_2_, and from methylated compounds [50, 51].

The obtained results are in accordance with those of a recent study on the anaerobic digestion of molasses wastewater in a UASB reactor, which revealed the significant contribution of *Lactococcus* and *Methanosaeta* [52]. These authors analyzed cDNA obtained by reverse transcription of RNA isolated from methane-yielding sludge samples. They proposed lactate as the major fermentation product being subsequently oxidized to acetate, a substrate for *Methanosaeta*. As a lactic acid bacterium, *Lactococcus* is a lactate producer, whereas *Methanosaeta* utilizes acetate generated by lactate oxidation. However, the identification of lactate oxidizers was ambiguous. In another study, analysis of the microbial community during corn stalk silage digestion revealed an abundance of *Lactobacillus* and *Acetobacter* species as well as a high concentration of lactic acid. During processing of the corn stalk silage to methane, *Methanosaeta* species were the predominant methanogens [38]. Wu et al. [43] used a two-stage system for the anaerobic digestion of fruit and vegetable waste. Lactate was a dominant product and *Lactobacillus* was the predominant microorganism during the first acidogenic phase, whereas *Methanosaeta* was the predominant methanogen in the second methane-producing phase. The scenario that lactose can be readily converted to lactate by homolactic bacteria, e.g., *Streptococcus lactis*, lactate to acetate by *Clostridium formicoaceticum* and, finally, acetate to methane by *Methanosarcina mazei* was shown before using pure cultures of microorganisms [53]. From the available data, it may be concluded that an abundance of lactate in the acetogenic stage of AD favours the acetoclastic pathway of methanogenesis.

The oxidation of lactate to acetate also yields hydrogen and carbon dioxide as well as other products such as propionate [4, 15, 45]. Furthermore, anaerobic oxidation of butyrate or propionate (present in the processed medium at low concentrations) requires syntrophic metabolic processes that generate hydrogen, formate, and carbon dioxide used directly by partner hydrogenotrophic methanogens [9]. This may explain the minor contribution of hydrogenotrophic methanogens in the methane-yielding microbial communities processing a lactate-rich substrate. The fate of lactate during the acetogenic step of AD is summarized in Fig. 6 and the associated table.

The results of this and other recent studies (cited above) have contributed to solving the anaerobic digestion puzzle by shedding light on the processing of lactate during the acetogenic step of AD.

### Genes for lactate utilization under anaerobic conditions

The above considerations suggest that the lactate oxidisers constitute a physiological group in methane-yielding microbial communities.

Using next-generation DNA sequencing of 16S rRNA gene fragment libraries, the microbial composition of the communities processing artificial lactate-rich media to methane was determined. The identified phyla match those that are usually found in anaerobic digesters/biogas plants. We next selected species and genera found in both bioreactors, whose genomes are available in databases, and searched these sequences for genes encoding proteins involved in anaerobic lactate oxidation. The majority of species identified in the microbial communities are potentially able to use lactate as an energy source and they can be divided into several groups (Fig. 3). The first group (representatives of *Firmicutes*, *Tissierellia*, *Synergistetes*, *Spirochaetes*, and *Actinobacteria*) uses the enzymatic machinery recently described in *Acetobacterium woodii*, with the FAD-dependent lactate dehydrogenase GlcD and EtfA/B electron transfer complex that converts lactate to acetate, carbon dioxide, and hydrogen as intermediates [16]. In this group, a minimal GlcD-EtfA/B operon was identified. In the second group (representatives of *Bacteroidetes*, *Firmicutes*, *δ*-*Proteobacteria*, and *Chloroflexi*), the GlcD dehydrogenase can occur with Fe-S oxidoreductase GlpC instead of EtfA/B, either as a fusion protein, two separate units, or both. GlpC is an iron–sulfur cluster-binding protein domain found in the FAD-dependent D-lactate dehydrogenase subunit of *Desulfovibrio vulgaris* Hildenborough [54].

Many representatives of *Bacteroidetes*, *δ*-*Proteobacteria*, *Verrucomicrobia*, *Firmicutes*, *Terrabacteria,* and *Nitrospirae* possess the LutB, LutC protein domains found in the l-lactate dehydrogenase subunit as demonstrated for *Desulfovibrio vulgaris* Hildenborough [54]. Some representatives of the *Firmicutes* as well as *δ*-*Proteobacteria* possess genes encoding both the GlcD-EtfA/B proteins and the GlcD/GlpC and Lut proteins.

The genes encoding both d-lactate and l-lactate dehydrogenases in *Desulfovibrio vulgaris* are part of the *luo* operon (lactate utilization operon). This operon is conserved in other genera of sulfate-reducing bacteria. Furthermore, a high degree of redundancy is observed in the lactate utilization machinery of *D. vulgaris.* Members of the genus *Desulfovibrio* are capable of syntrophic growth on lactate and ethanol with hydrogenotrophic methane-producing partners in the absence of sulfate. Since sulfate reduction is thermodynamically more favourable than methanogenesis, such a syntrophic metabolism is possible only when electron acceptors such as sulfate are absent [1, 9].

It is noteworthy that the presence of LldP permease and LarA racemase might not be obligatory for these potential lactate oxidizers, which would suggest, especially in the case of the former, either the passive diffusion of lactate or another mechanism of transport across the cell envelope.

Summing up, the genes encoding enzymes involved in anaerobic lactate metabolism are widespread in the domain *Bacteria.*

Notably, the genes encoding all nine proteins of the reductive acetyl-CoA (Wood–Ljungdahl) pathway were detected in the analyzed genomes. This indicates the capacity of the microbial communities to form acetate from carbon dioxide and hydrogen.

Although the bioinformatics analysis presented above is detailed and informative, it is based only on 16S rDNA amplicon verification and the information concerning the whole genomes has been retrieved from databases. Further studies using biochemical analysis and meta-omics approaches are warranted.

## Conclusions

Studies on methane-yielding microbial communities processing lactate-rich artificial media with a fixed composition revealed that (i) lactate oxidisers constitute a physiological group in the bacterial communities and the genes for lactate utilization under anaerobic conditions are widespread in the domain *Bacteria*; (ii) among *Archaea* present in the bioreactors the order *Methano sarcinales* predominated. The acetoclastic pathway of methane formation was confirmed by analysis of the stable carbon isotope composition of methane and carbon dioxide. The energy output on lactate degradation to the substrates for methanogenesis is the lowest in comparison to oxidation of acetate, butyrate, and propionate; therefore, attractiveness of lactate as an intermediate during anaerobic digestion is the highest. We postulate that lactate is oxidized mainly to acetate during acetogenesis and this comprises the acetotrophic pathway of methanogenesis. The results contribute to the knowledge of metabolic pathways of anaerobic digestion. They can also help to understand and improve the operation of biogas plants.

## Additional files

**Additional file 1.** Genera present in both the M1A and M1B communities with corresponding read numbers used in searching for genes of the Wood–Ljungdahl pathway.

**Additional file 2.** Enzymes (identified by EC number) of prokaryotic carbon fixation pathways in bioreactor microbial communities M1A and M1B, including the enzymes comprising the Wood–Ljungdahl pathway.

**Additional file 3.** Number of reads assigned to respective taxonomic branches, M1A microbial community.

**Additional file 4.** Number of reads assigned to respective taxonomic branches, M1 B microbial community.

**Additional file 5.** Lactate utilization genes in identified species and genera with known genome sequences.

**Additional file 6.** Genes encoding all nine proteins of the reductive acetyl-CoA (Wood–Ljungdahl) pathway (KEGG Module: M00377) found in both microbial communities, M1A and M1B.

**Additional file 7.** Stable carbon isotopic composition of substrates and fermentation gas.

**Additional file 8.** Performance of the M1B methane-yielding microbial community processing a lactate-rich artificial medium between 44th and 50th week of cultivation.

## Abbreviations

AD: anaerobic digestion
COD: chemical oxygen demand
Etf: electron transfer flavoprotein
HPLC: high-performance liquid chromatography
HRT: hydraulic retention time
KEGG: Kyoto Encyclopedia of Genes and Genomes
LarA: lactate racemase
LDH: lactate dehydrogenase
LldP: lactate permease
ORP: oxidation/reduction potential
oxidative CODH/ACS: oxidative carbon-monoxide dehydrogenase/acetyl-CoA synthase pathway
SD: standard deviation
UASB bioreactor: upflow anaerobic sludge blanket bioreactor
