# Supplementary material for: Methane-yielding microbial communities processing lactate-rich substrates: a piece of the anaerobic digestion puzzle
Source: Biotechnol Biofuels. 2018 Apr 21;11:116. doi: 10.1186/s13068-018-1106-z (PMC5910564; doi:10.1186/s13068-018-1106-z)
Supplement: Supplementary file 3 — Additional file 3. Number of reads assigned to respective taxonomic branches, M1A microbial community. [file 13068_2018_1106_MOESM3_ESM.docx]

Additional file 3. Number of reads assigned to respective taxonomic branches, M1A microbial community.

| Kingdom | Phylum | Class | Order | Family | Genus | Species | num_hits | %_hits |
| --- | --- | --- | --- | --- | --- | --- | --- | --- |
| Unclassified |  |  |  |  |  |  | 194 | 0.168 |
| Archaea | Euryarchaeota | Methanomicrobia | Methanosarcinales | Methanosaetaceae | Methanosaeta | concilii | 22639 | 19.636 |
| Bacteria | Proteobacteria | Deltaproteobacteria | Syntrophobacterales | Desulfobacteraceae |  |  | 6502 | 5.639 |
| Bacteria | Bacteroidetes | Sphingobacteriia | Sphingobacteriales | Sphingobacteriaceae | Pedobacter | kwangyangensis | 6464 | 5.606 |
| Bacteria | Firmicutes | Clostridia | Clostridiales | Veillonellaceae | Anaerovibrio |  | 6359 | 5.515 |
| Bacteria | Proteobacteria | Deltaproteobacteria | Syntrophobacterales | Syntrophaceae | Syntrophus |  | 5739 | 4.978 |
| Bacteria | Synergistetes | Synergistia | Synergistales | Synergistaceae | Synergistes |  | 5349 | 4.639 |
| Bacteria | Firmicutes | Clostridia | Clostridiales | Syntrophomonadaceae | Syntrophomonas |  | 5130 | 4.449 |
| Bacteria | Actinobacteria | Acidimicrobiia | Acidimicrobiales | Acidimicrobiaceae | Acidimicrobium |  | 4000 | 3.469 |
| Bacteria | Proteobacteria | Deltaproteobacteria | Desulfovibrionales | Desulfovibrionaceae | Desulfovibrio |  | 2718 | 2.357 |
| Bacteria |  |  |  |  |  |  | 2262 | 1.962 |
| Bacteria | Firmicutes | Clostridia | Clostridiales | Veillonellaceae | Megasphaera |  | 2189 | 1.899 |
| Bacteria | Firmicutes | Clostridia | Clostridiales | Syntrophomonadaceae | Syntrophomonas | wolfei | 2079 | 1.803 |
| Bacteria | Bacteroidetes |  |  |  |  |  | 1706 | 1.48 |
| Bacteria | Synergistetes | Synergistia | Synergistales | Aminiphilaceae | Aminiphilus | circumscriptus | 1442 | 1.251 |
| Bacteria | Cyanobacteria | Nostocophycideae | Nostocales | Nostocaceae |  |  | 1333 | 1.156 |
| Bacteria | Bacteroidetes | Bacteroidia | Bacteroidales | Bacteroidaceae | Bacteroides | denticanum | 1302 | 1.129 |
| Bacteria | Firmicutes | Clostridia | Thermoanaerobacterales | Thermovenabulum | Thermovenabulum | ferriorganovorum | 1277 | 1.108 |
| Bacteria | Firmicutes | Clostridia | Clostridiales | Lachnospiraceae | Blautia |  | 1255 | 1.089 |
| Bacteria | Spirochaetes | Spirochaetes | Spirochaetales | Spirochaetaceae | Treponema |  | 1218 | 1.056 |
| Bacteria | Proteobacteria |  |  |  |  |  | 1153 | 1 |
| Bacteria | Proteobacteria | Deltaproteobacteria | Desulfovibrionales | Desulfovibrionaceae | Desulfovibrio | fairfieldensis | 1056 | 0.916 |
| Bacteria | Proteobacteria | Deltaproteobacteria | Desulfovibrionales | Desulfovibrionaceae | Desulfovibrio | simplex | 1028 | 0.892 |
| Bacteria | Synergistetes | Synergistia | Synergistales | Dethiosulfovibrionaceae | Aminobacterium |  | 993 | 0.861 |
| Bacteria | Firmicutes | Clostridia | Clostridiales | Clostridiaceae | Clostridium | termitidis | 911 | 0.79 |
| Bacteria | Firmicutes | Clostridia | Clostridiales |  |  |  | 889 | 0.771 |
| Bacteria | Firmicutes | Clostridia | Clostridiales | Veillonellaceae | Propionispora | hippei | 841 | 0.729 |
| Bacteria | Firmicutes | Clostridia | Clostridiales | Syntrophomonadaceae | Syntrophomonas | curvata | 803 | 0.696 |
| Bacteria | Bacteroidetes | Flavobacteriia | Flavobacteriales | Flavobacteriaceae | Flavobacterium |  | 778 | 0.675 |
| Bacteria | Bacteroidetes | Bacteroidia | Bacteroidales |  |  |  | 699 | 0.606 |
| Bacteria | Firmicutes | Clostridia | Clostridiales | Veillonellaceae | Megasphaera | hominis | 695 | 0.603 |
| Archaea | Euryarchaeota | Methanomicrobia | Methanomicrobiales | Methanospirillaceae | Methanospirillum |  | 693 | 0.601 |
| Bacteria | Firmicutes | Clostridia | Clostridiales | Clostridiaceae | Alkaliphilus |  | 687 | 0.596 |
| Archaea | Euryarchaeota | Methanobacteria | Methanobacteriales | Methanobacteriaceae | Methanobacterium |  | 680 | 0.59 |
| Archaea | Euryarchaeota | Methanomicrobia | Methanomicrobiales | Methanocorpusculaceae | Methanocorpusculum | labreanum | 627 | 0.544 |
| Bacteria | Firmicutes | Clostridia | Clostridiales | Ruminococcaceae | Oscillospira | eae | 555 | 0.481 |
| Bacteria | Firmicutes | Clostridia |  |  |  |  | 553 | 0.48 |
| Bacteria | Firmicutes | Clostridia | Clostridiales | Syntrophomonadaceae | Syntrophomonas | palmitatica | 543 | 0.471 |
| Bacteria | Firmicutes | Clostridia | Clostridiales | Syntrophomonadaceae | Syntrophomonas | cellicola | 508 | 0.441 |
| Bacteria | Firmicutes | Clostridia | Clostridiales | Clostridiaceae | Alkaliphilus | crotonatoxidans | 504 | 0.437 |
| Bacteria | Bacteroidetes | Flavobacteriia | Flavobacteriales | Flavobacteriaceae | Myroides |  | 493 | 0.428 |
| Archaea | Euryarchaeota | Methanomicrobia | Methanosarcinales | Methanosaetaceae | Methanosaeta |  | 463 | 0.402 |
| Bacteria | Bacteroidetes | Bacteroidia | Bacteroidales | Bacteroidaceae | Bacteroides | xylanisolvens | 447 | 0.388 |
| Bacteria | Firmicutes | Clostridia | Clostridiales | Syntrophomonadaceae | Syntrophomonas | sapovorans | 446 | 0.387 |
| Bacteria | Firmicutes | Clostridia | Clostridiales | Veillonellaceae |  |  | 428 | 0.371 |
| Bacteria | Bacteroidetes | Flavobacteriia | Flavobacteriales | Flavobacteriaceae |  |  | 427 | 0.37 |
| Bacteria | Firmicutes | Clostridia | Clostridiales | Clostridiaceae |  |  | 414 | 0.359 |
| Bacteria | Firmicutes | Clostridia | Clostridiales | Clostridiaceae | Clostridium |  | 404 | 0.35 |
| Bacteria | Caldithrix | Caldithrixae | Caldithrixales | Caldithrixaceae | Caldithrix |  | 391 | 0.339 |
| Bacteria | Firmicutes | Clostridia | Clostridiales | Clostridiaceae | Clostridium | alkalicellulosi | 387 | 0.336 |
| Bacteria | Spirochaetes | Spirochaetes | Sphaerochaetales | Sphaerochaetaceae | Sphaerochaeta |  | 385 | 0.334 |
| Bacteria | Bacteroidetes | Flavobacteriia | Flavobacteriales | Flavobacteriaceae | Chryseobacterium |  | 382 | 0.331 |
| Bacteria | Firmicutes | Clostridia | Clostridiales | Ruminococcaceae | Oscillospira |  | 355 | 0.308 |
| Bacteria | Synergistetes | Synergistia | Synergistales | Synergistaceae | Candidatus Tammella | caduceiae | 345 | 0.299 |
| Bacteria | Synergistetes | Synergistia | Synergistales | Dethiosulfovibrionaceae | Pyramidobacter |  | 318 | 0.276 |
| Bacteria | Firmicutes | Clostridia | Clostridiales | Clostridiaceae | Sedimentibacter | hydroxybenzoicus | 291 | 0.252 |
| Bacteria | Cyanobacteria |  |  |  |  |  | 288 | 0.25 |
| Bacteria | Firmicutes | Clostridia | Clostridiales | Clostridiaceae | Clostridium | taeniosporum | 281 | 0.244 |
| Bacteria | Tenericutes | Mollicutes | Acholeplasmatales | Acholeplasmataceae | Acholeplasma | cavigenitalium | 278 | 0.241 |
| Bacteria | Bacteroidetes | Flavobacteriia | Flavobacteriales | Flavobacteriaceae | Gramella | forsetii | 276 | 0.239 |
| Bacteria | Verrucomicrobia | Spartobacteria | Chthoniobacterales | Chthoniobacteraceae | Chthoniobacter | flavus | 259 | 0.225 |
| Bacteria | Synergistetes | Synergistia | Synergistales | Synergistaceae | Candidatus Tammella |  | 253 | 0.219 |
| Bacteria | Bacteroidetes | Bacteroidia | Bacteroidales | Bacteroidaceae | Bacteroides |  | 245 | 0.212 |
| Bacteria | Firmicutes | Clostridia | Clostridiales | Clostridiaceae | Sedimentibacter |  | 236 | 0.205 |
| Bacteria | Synergistetes | Synergistia | Synergistales | Dethiosulfovibrionaceae | Dethiosulfovibrio |  | 224 | 0.194 |
| Bacteria | Bacteroidetes | Sphingobacteriia | Sphingobacteriales | Sphingobacteriaceae | Sphingobacterium | shayense | 224 | 0.194 |
| Bacteria | Synergistetes | Synergistia | Synergistales | Synergistaceae | Thermococcus |  | 217 | 0.188 |
| Bacteria | Bacteroidetes | Flavobacteriia | Flavobacteriales | Flavobacteriaceae | Flavobacterium | terrigena | 212 | 0.184 |
| Bacteria | Spirochaetes | Spirochaetes | Spirochaetales | Spirochaetaceae | Treponema | brennaborense | 209 | 0.181 |
| Bacteria | Bacteroidetes | Flavobacteriia | Flavobacteriales | Flavobacteriaceae | Aquimarina | macrocephali | 192 | 0.167 |
| Bacteria | Firmicutes | Clostridia | Clostridiales | Clostridiaceae | Peptoniphilus | coxii | 192 | 0.167 |
| Bacteria | Proteobacteria | Alphaproteobacteria | Sphingomonadales | Sphingomonadaceae | Sphingobium |  | 191 | 0.166 |
| Bacteria | Firmicutes | Clostridia | Clostridiales | Clostridiaceae | Clostridium | caenicola | 190 | 0.165 |
| Bacteria | Thermotogae | Thermotogae | Thermotogales | Thermotogaceae | Kosmotoga |  | 186 | 0.161 |
| Bacteria | Firmicutes | Clostridia | Clostridiales | Clostridiaceae | Caloramator | mitchellensis | 179 | 0.155 |
| Bacteria | Cyanobacteria | Synechococcophycideae | Pseudanabaenales | Pseudanabaenaceae | Leptolyngbya | laminosa | 175 | 0.152 |
| Bacteria | Firmicutes | Bacilli | Thermicanales | Thermicanaceae | Thermicanus |  | 159 | 0.138 |
| Bacteria | Proteobacteria | Gammaproteobacteria | Chromatiales | Halothiobacillaceae | Halothiobacillus | neapolitanus | 148 | 0.128 |
| Bacteria | Bacteroidetes | Bacteroidia | Bacteroidales | Bacteroidaceae | Bacteroides | cellulosilyticus | 145 | 0.126 |
| Bacteria | Firmicutes |  |  |  |  |  | 145 | 0.126 |
| Bacteria | Spirochaetes | Spirochaetes | Sphaerochaetales | Sphaerochaetaceae | Sphaerochaeta | globus | 144 | 0.125 |
| Bacteria | Tenericutes | Mollicutes | Acholeplasmatales | Acholeplasmataceae | Acholeplasma |  | 135 | 0.117 |
| Bacteria | Synergistetes | Synergistia | Synergistales |  |  |  | 133 | 0.115 |
| Archaea | Euryarchaeota | Methanomicrobia | Methanomicrobiales | Methanospirillaceae | Methanospirillum | hungatei | 124 | 0.108 |
| Bacteria | Proteobacteria | Deltaproteobacteria | Desulfuromonadales | Geobacteraceae | Geobacter |  | 118 | 0.102 |
| Bacteria | Proteobacteria | Deltaproteobacteria | Desulfovibrionales | Desulfohalobiaceae | Desulfonauticus | autotrophicus | 114 | 0.099 |
| Bacteria | Bacteroidetes | Sphingobacteriia | Sphingobacteriales | Sphingobacteriaceae |  |  | 113 | 0.098 |
| Bacteria | Bacteroidetes | Bacteroidia | Bacteroidales | Porphyromonadaceae | Parabacteroides | goldsteinii | 113 | 0.098 |
| Bacteria | Firmicutes | Clostridia | Clostridiales | Ruminococcaceae | Oscillospira | guilliermondii | 112 | 0.097 |
| Bacteria | Bacteroidetes | Sphingobacteriia | Sphingobacteriales | Sphingobacteriaceae | Pedobacter |  | 106 | 0.092 |
| Bacteria | Bacteroidetes | Flavobacteriia | Flavobacteriales | Flavobacteriaceae | Polaribacter | butkevichii | 103 | 0.089 |
| Bacteria | Firmicutes | Clostridia | Clostridiales | Veillonellaceae | Pectinatus | cerevisiiphilus | 102 | 0.088 |
| Archaea | Euryarchaeota | Methanomicrobia | Methanomicrobiales | Methanomicrobiaceae | Methanoculleus |  | 101 | 0.088 |
| Bacteria | Firmicutes | Clostridia | Thermoanaerobacterales | Caldicellulosiruptoraceae | Caldicellulosiruptor |  | 99 | 0.086 |
| Bacteria | Proteobacteria | Deltaproteobacteria | Syntrophobacterales | Desulfobacteraceae | Desulfosarcina | ovata | 94 | 0.082 |
| Bacteria | Proteobacteria | Deltaproteobacteria | Desulfovibrionales | Desulfovibrionaceae | Desulfovibrio | alcoholivorans | 94 | 0.082 |
| Bacteria | Synergistetes | Synergistia | Synergistales | Synergistaceae |  |  | 90 | 0.078 |
| Bacteria | Proteobacteria | Deltaproteobacteria | Syntrophobacterales | Desulfobacteraceae | Desulfofrigus | oceanense | 81 | 0.07 |
| Bacteria | Bacteroidetes | Sphingobacteriia | Sphingobacteriales | Flexibacteraceae | Runella | limosa | 80 | 0.069 |
| Bacteria | Bacteroidetes | Bacteroidia | Bacteroidales | Porphyromonadaceae | Dysgonomonas | wimpennyi | 79 | 0.069 |
| Bacteria | Verrucomicrobia |  |  |  |  |  | 77 | 0.067 |
| Bacteria | Bacteroidetes | Sphingobacteriia | Sphingobacteriales | Rhodothermaceae | Rhodothermus | clarus | 77 | 0.067 |
| Bacteria | Firmicutes | Clostridia | Clostridiales | Clostridiaceae | Clostridium | acetireducens | 76 | 0.066 |
| Bacteria | Bacteroidetes | Bacteroidia | Bacteroidales | Porphyromonadaceae | Parabacteroides |  | 76 | 0.066 |
| Bacteria | Bacteroidetes | Sphingobacteriia | Sphingobacteriales | Chitinophagaceae |  |  | 76 | 0.066 |
| Bacteria | Firmicutes | Clostridia | Clostridiales | Clostridiaceae | Natronincola |  | 75 | 0.065 |
| Bacteria | Proteobacteria | Deltaproteobacteria | Desulfovibrionales | Desulfovibrionaceae | Desulfovibrio | oryzae | 75 | 0.065 |
| Bacteria | Firmicutes | Clostridia | Clostridiales | Clostridiaceae | Clostridium | tyrobutyricum | 73 | 0.063 |
| Bacteria | Firmicutes | Clostridia | Clostridiales | Veillonellaceae | Anaerovibrio | lipolyticus | 69 | 0.06 |
| Bacteria | Proteobacteria | Gammaproteobacteria | Enterobacteriales | Enterobacteriaceae | Citrobacter | freundii | 67 | 0.058 |
| Bacteria | Firmicutes | Clostridia | Clostridiales | Clostridiaceae | Caloramator | uzoniensis | 66 | 0.057 |
| Bacteria | Firmicutes | Clostridia | Clostridiales | Clostridiaceae | Clostridium | histolyticum | 63 | 0.055 |
| Bacteria | Firmicutes | Clostridia | Clostridiales | Clostridiaceae | Alkaliphilus | peptidifermentans | 63 | 0.055 |
| Bacteria | Firmicutes | Clostridia | Clostridiales | Lachnospiraceae | Johnsonella | ignava | 62 | 0.054 |
| Bacteria | Proteobacteria | Alphaproteobacteria | Rhodospirillales | Acetobacteraceae | Swaminathania |  | 62 | 0.054 |
| Archaea | Euryarchaeota | Methanomicrobia | Methanomicrobiales | Methanocorpusculaceae | Methanocorpusculum |  | 62 | 0.054 |
| Bacteria | Chloroflexi | Thermobacula | Thermobaculales | Thermobaculaceae | Thermobaculum | terrenum | 60 | 0.052 |
| Bacteria | Firmicutes | Clostridia | Clostridiales | Veillonellaceae | Acidaminococcus | fermentans | 58 | 0.05 |
| Bacteria | Firmicutes | Clostridia | Clostridiales | Heliobacteriaceae | Heliorestis | baculata | 56 | 0.049 |
| Bacteria | Firmicutes | Clostridia | Clostridiales | Veillonellaceae | Megasphaera | sueciensis | 56 | 0.049 |
| Bacteria | Nitrospirae | Nitrospira | Nitrospirales | Thermodesulfovibrionaceae | Thermodesulfovibrio | thiophilus | 53 | 0.046 |
| Bacteria | Bacteroidetes | Sphingobacteriia | Sphingobacteriales | Amoebophilaceae | Candidatus Amoebophilus | asiaticus | 52 | 0.045 |
| Bacteria | Proteobacteria | Deltaproteobacteria | Desulfovibrionales | Desulfovibrionaceae | Desulfovibrio | desulfuricans | 52 | 0.045 |
| Bacteria | Thermotogae | Thermotogae | Thermotogales | Thermotogaceae | Fervidobacterium |  | 50 | 0.043 |
| Bacteria | Bacteroidetes | Bacteroidia | Bacteroidales | Bacteroidaceae | Bacteroides | ovatus | 50 | 0.043 |
| Archaea | Euryarchaeota | Methanomicrobia | Methanomicrobiales | Methanomicrobiaceae | Methanofollis | liminatans | 50 | 0.043 |
| Bacteria | Firmicutes | Clostridia | Clostridiales | Veillonellaceae | Acidaminococcus |  | 50 | 0.043 |
| Bacteria | Proteobacteria | Deltaproteobacteria | Desulfovibrionales | Desulfovibrionaceae | Desulfovibrio | burkinensis | 49 | 0.042 |
| Bacteria | Proteobacteria | Deltaproteobacteria | Desulfovibrionales | Desulfohalobiaceae | Desulfonauticus | submarinus | 48 | 0.042 |
| Bacteria | Chloroflexi | Anaerolineae | Anaerolineales | Anaerolinaceae | Longilinea | arvoryzae | 47 | 0.041 |
| Bacteria | Bacteroidetes | Flavobacteriia | Flavobacteriales | Flavobacteriaceae | Tenacibaculum |  | 47 | 0.041 |
| Bacteria | Bacteroidetes | Bacteroidia | Bacteroidales | Porphyromonadaceae | Porphyromonas | canis | 46 | 0.04 |
| Bacteria | Firmicutes | Clostridia | Clostridiales | Peptococcaceae | Peptococcus | niger | 46 | 0.04 |
| Bacteria | Proteobacteria | Deltaproteobacteria | Syntrophobacterales |  |  |  | 46 | 0.04 |
| Bacteria | Firmicutes | Clostridia | Clostridiales | Clostridiaceae | Clostridium | thermosuccinogenes | 46 | 0.04 |
| Bacteria | Bacteroidetes | Sphingobacteriia | Sphingobacteriales |  |  |  | 46 | 0.04 |
| Bacteria | Firmicutes | Clostridia | Clostridiales | Clostridiaceae | Clostridium | straminisolvens | 45 | 0.039 |
| Bacteria | Firmicutes | Clostridia | Clostridiales | Eubacteriaceae | Acetobacterium |  | 45 | 0.039 |
| Bacteria | Firmicutes | Clostridia | Clostridiales | Syntrophomonadaceae | Syntrophomonas | bryantii | 44 | 0.038 |
| Bacteria | Firmicutes | Clostridia | Clostridiales | Lachnospiraceae | Lachnospira | pectinoschiza | 43 | 0.037 |
| Bacteria | Proteobacteria | Betaproteobacteria |  |  |  |  | 41 | 0.036 |
| Bacteria | Actinobacteria | Actinobacteria |  |  |  |  | 40 | 0.035 |
| Bacteria | Firmicutes | Clostridia | Clostridiales | Ruminococcaceae | Anaerotruncus | colihominis | 39 | 0.034 |
| Bacteria | Spirochaetes | Spirochaetes | Spirochaetales | Spirochaetaceae | Treponema | zioleckii | 39 | 0.034 |
| Bacteria | Proteobacteria | Alphaproteobacteria | Sphingomonadales | Sphingomonadaceae |  |  | 39 | 0.034 |
| Bacteria | Firmicutes | Clostridia | Clostridiales | Lachnospiraceae | Blautia | coccoides | 38 | 0.033 |
| Bacteria | Firmicutes | Clostridia | Clostridiales | Veillonellaceae | Selenomonas | infelix | 34 | 0.029 |
| Bacteria | Firmicutes | Clostridia | Clostridiales | Dehalobacteriaceae | Dehalobacterium |  | 34 | 0.029 |
| Bacteria | Proteobacteria | Alphaproteobacteria | Sphingomonadales | Sphingomonadaceae | Sphingomonas | oligophenolica | 34 | 0.029 |
| Bacteria | Proteobacteria | Deltaproteobacteria | Syntrophobacterales | Desulfobacteraceae | Desulfobacter |  | 33 | 0.029 |
| Bacteria | Firmicutes | Clostridia | Natranaerobiales | Anaerobrancaceae | Anaerobranca | zavarzinii | 32 | 0.028 |
| Archaea | Euryarchaeota | Methanomicrobia | Methanomicrobiales | Methanomicrobiaceae | Methanoculleus | receptaculi | 32 | 0.028 |
| Archaea | Euryarchaeota | Methanomicrobia | Methanomicrobiales | Methanomicrobiaceae | Methanoculleus | palmolei | 31 | 0.027 |
| Bacteria | Firmicutes | Clostridia | Clostridiales | Clostridiaceae | Clostridium | cadaveris | 31 | 0.027 |
| Bacteria | Chloroflexi | Anaerolineae | Caldilineales | Caldilineaceae | Caldilinea | tarbellica | 30 | 0.026 |
| Bacteria | Chloroflexi | Anaerolineae | Anaerolineales | Anaerolinaceae | Bellilinea | caldifistulae | 30 | 0.026 |
| Archaea | Euryarchaeota | Methanobacteria | Methanobacteriales | Methanobacteriaceae | Methanobacterium | kanagiense | 30 | 0.026 |
| Bacteria | Proteobacteria | Deltaproteobacteria | Desulfovibrionales | Desulfovibrionaceae | Desulfovibrio | intestinalis | 29 | 0.025 |
| Bacteria | Actinobacteria | Actinobacteria | Actinomycetales |  |  |  | 29 | 0.025 |
| Bacteria | Firmicutes | Clostridia | Clostridiales | Carboxydocellaceae | Carboxydocella | ferrireducens | 28 | 0.024 |
| Archaea | Euryarchaeota | Methanomicrobia | Methanomicrobiales | Methanocorpusculaceae | Methanocorpusculum | parvum | 28 | 0.024 |
| Archaea | Euryarchaeota | Methanomicrobia | Methanosarcinales |  |  |  | 28 | 0.024 |
| Bacteria | Verrucomicrobia | Methylacidiphilae | Methylacidiphilales | Methylacidiphilaceae | Candidatus Methylacidiphilum |  | 28 | 0.024 |
| Bacteria | Bacteroidetes | Bacteroidia | Bacteroidales | Bacteroidaceae | Bacteroides | graminisolvens | 28 | 0.024 |
| Bacteria | Proteobacteria | Alphaproteobacteria | Sphingomonadales | Sphingomonadaceae | Sphingomonas | insulae | 27 | 0.023 |
| Bacteria | Proteobacteria | Alphaproteobacteria | Rhodospirillales | Acetobacteraceae | Acetobacter | pasteurianus | 27 | 0.023 |
| Bacteria | Proteobacteria | Deltaproteobacteria | Syntrophobacterales | Syntrophaceae | Desulfomonile | tiedjei | 26 | 0.023 |
| Bacteria | Firmicutes | Clostridia | Coriobacteriales | Coriobacteriaceae | Slackia |  | 26 | 0.023 |
| Bacteria | Firmicutes | Erysipelotrichi | Erysipelotrichales | Erysipelotrichaceae | Erysipelothrix | muris | 25 | 0.022 |
| Bacteria | Firmicutes | Bacilli | Lactobacillales | Streptococcaceae | Streptococcus |  | 24 | 0.021 |
| Bacteria | Proteobacteria | Gammaproteobacteria | Pseudomonadales | Moraxellaceae | Acinetobacter | gerneri | 24 | 0.021 |
| Bacteria | Proteobacteria | Alphaproteobacteria | Rhodobacterales | Rhodobacteraceae | Paracoccus |  | 24 | 0.021 |
| Bacteria | Firmicutes | Clostridia | Thermoanaerobacterales | Thermoanaerobacteraceae |  |  | 24 | 0.021 |
| Bacteria | Firmicutes | Clostridia | Clostridiales | Heliobacteriaceae | Heliorestis |  | 23 | 0.02 |
| Bacteria | Bacteroidetes | Bacteroidia | Bacteroidales | Porphyromonadaceae | Dysgonomonas |  | 23 | 0.02 |
| Bacteria | Cyanobacteria | Nostocophycideae | Nostocales | Nostocaceae | Dolichospermum | curvum | 23 | 0.02 |
| Bacteria | Firmicutes | Clostridia | Clostridiales | Veillonellaceae | Veillonella | montpellierensis | 23 | 0.02 |
| Bacteria | Proteobacteria | Alphaproteobacteria | Rhodobacterales | Hyphomonadaceae | Hirschia | baltica | 22 | 0.019 |
| Bacteria | Proteobacteria | Deltaproteobacteria | Desulfobacterales | Desulfobulbaceae |  |  | 22 | 0.019 |
| Bacteria | Firmicutes | Clostridia | Clostridiales | Clostridiaceae | Sedimentibacter | saalensis | 22 | 0.019 |
| Bacteria | Chloroflexi |  |  |  |  |  | 21 | 0.018 |
| Bacteria | Proteobacteria | Deltaproteobacteria | Myxococcales | Nannocystaceae | Nannocystis |  | 21 | 0.018 |
| Bacteria | Firmicutes | Clostridia | Clostridiales | Peptococcaceae | Pelotomaculum |  | 21 | 0.018 |
| Archaea | Euryarchaeota | Methanomicrobia | Methanosarcinales | Methanosarcinaceae | Methanosarcina | mazei | 21 | 0.018 |
| Bacteria | Firmicutes | Clostridia | Clostridiales | Lachnospiraceae | Blautia | wexlerae | 21 | 0.018 |
| Bacteria | Firmicutes | Bacilli | Bacillales | Staphylococcaceae | Macrococcus |  | 21 | 0.018 |
| Bacteria | Proteobacteria | Gammaproteobacteria | Enterobacteriales | Enterobacteriaceae | Citrobacter |  | 20 | 0.017 |
| Bacteria | Firmicutes | Bacilli | Lactobacillales | Leuconostocaceae | Leuconostoc |  | 20 | 0.017 |
| Bacteria | Proteobacteria | Deltaproteobacteria | Syntrophobacterales | Desulfobacteraceae | Desulfosarcina |  | 20 | 0.017 |
| Bacteria | Proteobacteria | Deltaproteobacteria | Desulfuromonadales | Geobacteraceae | Geobacter | toluenoxydans | 20 | 0.017 |
| Bacteria | Firmicutes | Clostridia | Clostridiales | Eubacteriaceae | Acetobacterium | submarinus | 19 | 0.016 |
| Bacteria | Bacteroidetes | Bacteroidia | Bacteroidales | Bacteroidaceae | Bacteroides | paurosaccharolyticus | 19 | 0.016 |
| Bacteria | Synergistetes | Synergistia | Synergistales | Dethiosulfovibrionaceae | Aminobacterium | colombiense | 19 | 0.016 |
| Bacteria | Proteobacteria | Alphaproteobacteria | Rhodospirillales | Rhodospirillaceae |  |  | 19 | 0.016 |
| Bacteria | Bacteroidetes | Bacteroidia | Bacteroidales | Porphyromonadaceae |  |  | 18 | 0.016 |
| Bacteria | Firmicutes | Bacilli | Bacillales | Bacillaceae | Geobacillus |  | 18 | 0.016 |
| Bacteria | Spirochaetes | Spirochaetes | Sphaerochaetales | Sphaerochaetaceae | Sphaerochaeta | pleomorpha | 18 | 0.016 |
| Bacteria | Firmicutes | Clostridia | Thermoanaerobacterales | Thermoanaerobacteraceae | Moorella |  | 18 | 0.016 |
| Bacteria | Proteobacteria | Epsilonproteobacteria | Campylobacterales | Campylobacteraceae | Arcobacter | defluvii | 18 | 0.016 |
| Bacteria | Proteobacteria | Epsilonproteobacteria | Campylobacterales | Campylobacteraceae | Arcobacter | marinus | 18 | 0.016 |
| Bacteria | Tenericutes | Mollicutes | Acholeplasmatales | Acholeplasmataceae | Acholeplasma | parvum | 17 | 0.015 |
| Bacteria | Proteobacteria | Gammaproteobacteria | Enterobacteriales | Enterobacteriaceae | Klebsiella | oxytoca | 17 | 0.015 |
| Archaea |  |  |  |  |  |  | 17 | 0.015 |
| Bacteria | Proteobacteria | Deltaproteobacteria | Desulfuromonadales |  |  |  | 16 | 0.014 |
| Bacteria | Firmicutes | Clostridia | Clostridiales | Clostridiaceae | Caloramator |  | 16 | 0.014 |
| Bacteria | Proteobacteria | Deltaproteobacteria | Desulfuromonadales | Geobacteraceae | Geobacter | argillaceus | 16 | 0.014 |
| Bacteria | Proteobacteria | Alphaproteobacteria | Rhodospirillales | Acetobacteraceae | Gluconobacter |  | 16 | 0.014 |
| Bacteria | Firmicutes | Clostridia | Clostridiales | Veillonellaceae | Phascolarctobacterium | succinatutens | 16 | 0.014 |
| Bacteria | Proteobacteria | Deltaproteobacteria |  |  |  |  | 16 | 0.014 |
| Bacteria | Proteobacteria | Alphaproteobacteria | Rhodobacterales | Rhodobacteraceae | Paracoccus | sulfuroxidans | 15 | 0.013 |
| Bacteria | Firmicutes | Clostridia | Clostridiales | Clostridiaceae | Acidaminobacter | hydrogenoformans | 15 | 0.013 |
| Bacteria | Elusimicrobia | Elusimicrobia | Elusimicrobiales | Elusimicrobiaceae | Elusimicrobium |  | 15 | 0.013 |
| Bacteria | Proteobacteria | Deltaproteobacteria | Desulfovibrionales | Desulfovibrionaceae | Desulfovibrio | carbinolicus | 15 | 0.013 |
| Bacteria | Firmicutes | Clostridia | Clostridiales | Clostridiaceae | Clostridium | clariflavum | 14 | 0.012 |
| Bacteria | Synergistetes | Synergistia | Synergistales | Aminiphilaceae | Aminiphilus |  | 14 | 0.012 |
| Bacteria | Firmicutes | Clostridia | Clostridiales | Lachnospiraceae |  |  | 14 | 0.012 |
| Bacteria | Firmicutes | Clostridia | Clostridiales | Veillonellaceae | Negativicoccus | succinicivorans | 14 | 0.012 |
| Bacteria | Proteobacteria | Gammaproteobacteria |  |  |  |  | 14 | 0.012 |
| Bacteria | Cyanobacteria | Oscillatoriophycideae | Chroococcales | Phormidiaceae |  |  | 14 | 0.012 |
| Bacteria | Proteobacteria | Alphaproteobacteria |  |  |  |  | 13 | 0.011 |
| Bacteria | Proteobacteria | Deltaproteobacteria | Desulfuromonadales | Geobacteraceae | Geobacter | pickeringii | 13 | 0.011 |
| Bacteria | Bacteroidetes | Sphingobacteriia | Sphingobacteriales | Flexibacteraceae | Emticicia | oligotrophica | 13 | 0.011 |
| Bacteria | Firmicutes | Clostridia | Clostridiales | Ruminococcaceae | Anaerofilum |  | 13 | 0.011 |
| Bacteria | Proteobacteria | Deltaproteobacteria | Desulfovibrionales | Desulfovibrionaceae | Desulfovibrio | aminophilus | 13 | 0.011 |
| Bacteria | Proteobacteria | Gammaproteobacteria | Enterobacteriales | Enterobacteriaceae | Enterobacter | soli | 12 | 0.01 |
| Bacteria | Firmicutes | Bacilli | Lactobacillales |  |  |  | 12 | 0.01 |
| Bacteria | Actinobacteria | Actinobacteria | Bifidobacteriales | Bifidobacteriaceae | Bifidobacterium |  | 12 | 0.01 |
| Bacteria | Tenericutes | Mollicutes | Acholeplasmatales | Acholeplasmataceae |  |  | 12 | 0.01 |
| Archaea | Euryarchaeota | Methanomicrobia | Methanosarcinales | Methanosaetaceae | Methanosaeta | pelagica | 12 | 0.01 |
| Bacteria | Firmicutes | Bacilli | Gemellales | Gemellaceae | Gemella | cunicula | 12 | 0.01 |
| Bacteria | Firmicutes | Clostridia | Clostridiales | Eubacteriaceae | Pseudoramibacter | alactolyticus | 11 | 0.01 |
| Bacteria | Firmicutes | Clostridia | Clostridiales | Peptococcaceae | Desulfosporosinus |  | 11 | 0.01 |
| Bacteria | Proteobacteria | Deltaproteobacteria | Desulfobacterales | Desulfobulbaceae | Desulfobulbus | elongatus | 11 | 0.01 |
| Bacteria | Tenericutes | Mollicutes | Acholeplasmatales | Acholeplasmataceae | Acholeplasma | ales | 10 | 0.009 |
| Bacteria | Bacteroidetes | Flavobacteriia | Flavobacteriales | Flavobacteriaceae | Gramella |  | 10 | 0.009 |
| Bacteria | Proteobacteria | Alphaproteobacteria | Rhodospirillales |  |  |  | 10 | 0.009 |
| Bacteria | Firmicutes | Bacilli | Bacillales | Bacillaceae | Bacillus |  | 10 | 0.009 |
| Bacteria | Spirochaetes | Spirochaetes | Borreliales | Borreliaceae | Borrelia |  | 10 | 0.009 |
| Bacteria | Firmicutes | Bacilli |  |  |  |  | 10 | 0.009 |
| Bacteria | Proteobacteria | Epsilonproteobacteria | Campylobacterales | Campylobacteraceae | Sulfurospirillum |  | 10 | 0.009 |
| Bacteria | Firmicutes | Bacilli | Bacillales | Paenibacillaceae | Paenibacillus |  | 10 | 0.009 |
| Bacteria | Proteobacteria | Gammaproteobacteria | Alteromonadales | Alteromonadaceae | Marinobacter | arcticus | 10 | 0.009 |
| Bacteria | Firmicutes | Bacilli | Bacillales |  |  |  | 10 | 0.009 |
| Bacteria | Proteobacteria | Alphaproteobacteria | Sphingomonadales | Sphingomonadaceae | Sphingobium | cloacae | 10 | 0.009 |
| Archaea | Euryarchaeota | Methanobacteria | Methanobacteriales | Methanobacteriaceae | Methanobrevibacter |  | 10 | 0.009 |
| Bacteria | Actinobacteria | Actinobacteria | Actinomycetales | Microbacteriaceae |  |  | 9 | 0.008 |
| Bacteria | Proteobacteria | Deltaproteobacteria | Desulfovibrionales | Desulfovibrionaceae | Desulfovibrio | marrakechensis | 9 | 0.008 |
| Bacteria | Firmicutes | Clostridia | Clostridiales | Veillonellaceae | Acidaminococcus | intestini | 9 | 0.008 |
| Bacteria | Firmicutes | Clostridia | Clostridiales | Lachnospiraceae | Ruminococcus |  | 9 | 0.008 |
| Bacteria | Proteobacteria | Epsilonproteobacteria | Campylobacterales | Campylobacteraceae | Arcobacter | butzleri | 9 | 0.008 |
| Bacteria | Bacteroidetes | Bacteroidia | Bacteroidales | Bacteroidaceae | Bacteroides | thetaiotaomicron | 8 | 0.007 |
| Bacteria | Chloroflexi | Anaerolineae | Anaerolineales | Anaerolinaceae |  |  | 8 | 0.007 |
| Bacteria | Proteobacteria | Gammaproteobacteria | Enterobacteriales | Enterobacteriaceae | Erwinia | billingiae | 8 | 0.007 |
| Archaea | Euryarchaeota | Methanomicrobia | Methanosarcinales | Methanosarcinaceae |  |  | 8 | 0.007 |
| Bacteria | Proteobacteria | Alphaproteobacteria | Sphingomonadales | Sphingomonadaceae | Sphingobium | olei | 8 | 0.007 |
| Bacteria | Proteobacteria | Alphaproteobacteria | Rhodospirillales | Acetobacteraceae |  |  | 8 | 0.007 |
| Bacteria | Bacteroidetes | Sphingobacteriia | Sphingobacteriales | Sphingobacteriaceae | Olivibacter |  | 8 | 0.007 |
| Bacteria | Proteobacteria | Gammaproteobacteria | Enterobacteriales | Enterobacteriaceae |  |  | 7 | 0.006 |
| Archaea | Euryarchaeota | Methanomicrobia | Methanomicrobiales | Methanomicrobiaceae | Methanofollis |  | 7 | 0.006 |
| Bacteria | Synergistetes | Synergistia | Synergistales | Dethiosulfovibrionaceae | Aminobacterium | mobile | 7 | 0.006 |
| Archaea | Euryarchaeota | Halobacteria | Halobacteriales | Halobacteriaceae | Halorubrum | cibi | 7 | 0.006 |
| Bacteria | Firmicutes | Clostridia | Clostridiales | Peptococcaceae | Desulfotomaculum | indicum | 7 | 0.006 |
| Archaea | Euryarchaeota | Methanomicrobia | Methanosarcinales | Methanosarcinaceae | Methanosarcina | acetivorans | 7 | 0.006 |
| Bacteria | Synergistetes | Synergistia | Synergistales | Synergistaceae | Cloacibacillus |  | 7 | 0.006 |
| Bacteria | Proteobacteria | Deltaproteobacteria | Syntrophobacterales | Syntrophobacteraceae | Desulfacinum | subterraneum | 7 | 0.006 |
| Archaea | Euryarchaeota | Methanobacteria | Methanobacteriales | Methanobacteriaceae | Methanobacterium | beijingense | 7 | 0.006 |
| Bacteria | Firmicutes | Clostridia | Coriobacteriales | Coriobacteriaceae | Atopobium |  | 7 | 0.006 |
| Bacteria | Actinobacteria | Actinobacteria | Actinomycetales | Yaniellaceae | Yaniella |  | 7 | 0.006 |
| Archaea | Euryarchaeota | Methanomicrobia | Methanosarcinales | Methanosarcinaceae | Methanosarcina | siciliae | 7 | 0.006 |
| Bacteria | Proteobacteria | Deltaproteobacteria | Desulfovibrionales | Desulfovibrionaceae | Desulfovibrio | ferrophilus | 7 | 0.006 |
| Bacteria | Bacteroidetes | Flavobacteriia | Flavobacteriales | Flavobacteriaceae | Flavobacterium | antarcticum | 7 | 0.006 |
| Bacteria | Actinobacteria | Actinobacteria | Bifidobacteriales | Bifidobacteriaceae | Bifidobacterium | indicum | 6 | 0.005 |
| Bacteria | Proteobacteria | Gammaproteobacteria | Enterobacteriales | Enterobacteriaceae | Enterobacter |  | 6 | 0.005 |
| Bacteria | Synergistetes | Synergistia | Synergistales | Dethiosulfovibrionaceae | Pyramidobacter | piscolens | 6 | 0.005 |
| Bacteria | Cyanobacteria | Nostocophycideae | Stigonematales | Rivulariaceae | Calothrix | parietina | 6 | 0.005 |
| Archaea | Euryarchaeota | Methanobacteria | Methanobacteriales | Methanobacteriaceae | Methanobacterium | curvum | 6 | 0.005 |
| Bacteria | Thermotogae | Thermotogae | Thermotogales | Thermotogaceae | Fervidobacterium | pennivorans | 6 | 0.005 |
| Bacteria | Proteobacteria | Gammaproteobacteria | Enterobacteriales | Enterobacteriaceae | Citrobacter | werkmanii | 6 | 0.005 |
| Bacteria | Proteobacteria | Deltaproteobacteria | Desulfovibrionales |  |  |  | 6 | 0.005 |
| Bacteria | Firmicutes | Clostridia | Clostridiales | Clostridiaceae | Clostridium | homopropionicum | 6 | 0.005 |
| Bacteria | Firmicutes | Clostridia | Clostridiales | Peptococcaceae | Pelotomaculum | isophthalicicum | 6 | 0.005 |
| Bacteria | Bacteroidetes | Bacteroidia | Bacteroidales | Odoribacteraceae | Odoribacter | denticanis | 6 | 0.005 |
| Bacteria | Firmicutes | Bacilli | Bacillales | Bacillaceae | Geobacillus | thermoglucosidans | 6 | 0.005 |
| Bacteria | Proteobacteria | Gammaproteobacteria | Aeromonadales | Aeromonadaceae | Tolumonas | auensis | 5 | 0.004 |
| Archaea | Crenarchaeota | Thermoprotei | Thermoproteales | Thermoproteaceae | Thermocladium |  | 5 | 0.004 |
| Bacteria | Actinobacteria | Actinobacteria | Actinomycetales | Actinosynnemataceae |  |  | 5 | 0.004 |
| Bacteria | Proteobacteria | Alphaproteobacteria | Rickettsiales | Rickettsiaceae | Rickettsia |  | 5 | 0.004 |
| Archaea | Euryarchaeota | Methanomicrobia | Methanomicrobiales |  |  |  | 5 | 0.004 |
| Bacteria | Firmicutes | Clostridia | Clostridiales | Peptococcaceae | Desulfurispora | thermophila | 5 | 0.004 |
| Bacteria | Spirochaetes | Spirochaetes | Sphaerochaetales | Sphaerochaetaceae | Sphaerochaeta | coccoides | 5 | 0.004 |
| Bacteria | Bacteroidetes | Bacteroidia | Bacteroidales | Bacteroidaceae | Bacteroides | sartorii | 5 | 0.004 |
| Bacteria | Firmicutes | Clostridia | Clostridiales | Veillonellaceae | Megasphaera | paucivorans | 5 | 0.004 |
| Bacteria | Proteobacteria | Gammaproteobacteria | Enterobacteriales | Enterobacteriaceae | Enterobacter | nickellidurans | 5 | 0.004 |
| Bacteria | Proteobacteria | Gammaproteobacteria | Enterobacteriales | Enterobacteriaceae | Proteus | penneri | 5 | 0.004 |
| Bacteria | Bacteroidetes | Flavobacteriia | Flavobacteriales | Flavobacteriaceae | Flavobacterium | algicola | 5 | 0.004 |
| Bacteria | Thermotogae | Thermotogae | Thermotogales | Thermotogaceae | Marinitoga | okinawensis | 5 | 0.004 |
| Bacteria | Firmicutes | Clostridia | Natranaerobiales | Contubernalisaceae | Candidatus Contubernalis | alkalaceticum | 5 | 0.004 |
| Bacteria | Firmicutes | Bacilli | Lactobacillales | Carnobacteriaceae | Trichococcus |  | 5 | 0.004 |
| Bacteria | Proteobacteria | Epsilonproteobacteria | Campylobacterales | Campylobacteraceae | Arcobacter |  | 5 | 0.004 |
| Bacteria | Firmicutes | Clostridia | Thermoanaerobacterales | Thermoanaerobacteraceae | Tepidanaerobacter | syntrophicus | 5 | 0.004 |
| Bacteria | Bacteroidetes | Flavobacteriia | Flavobacteriales | Flavobacteriaceae | Polaribacter |  | 5 | 0.004 |
| Bacteria | Proteobacteria | Alphaproteobacteria | Rhodospirillales | Acetobacteraceae | Gluconobacter | kondonii | 5 | 0.004 |
| Bacteria | Firmicutes | Clostridia | Clostridiales | Peptococcaceae | Sporotomaculum | syntrophicum | 5 | 0.004 |
| Bacteria | Spirochaetes | Spirochaetes | Spirochaetales | Spirochaetaceae | Treponema | porcinum | 5 | 0.004 |
| Bacteria | Firmicutes | Bacilli | Lactobacillales | Enterococcaceae |  |  | 5 | 0.004 |
| Bacteria | Firmicutes | Bacilli | Bacillales | Bacillaceae | Lentibacillus | salinarum | 5 | 0.004 |
| Bacteria | Proteobacteria | Alphaproteobacteria | Sphingomonadales | Sphingomonadaceae | Sphingomonas |  | 4 | 0.003 |
| Bacteria | Bacteroidetes | Sphingobacteriia | Sphingobacteriales | Flexibacteraceae |  |  | 4 | 0.003 |
| Bacteria | Actinobacteria | Actinobacteria | Actinomycetales | Propionibacteriaceae |  |  | 4 | 0.003 |
| Bacteria | Chloroflexi | Dehalococcoidetes | Dehalococcoidales | Dehalococcoidaceae | Dehalogenimonas | lykanthroporepellens | 4 | 0.003 |
| Bacteria | Proteobacteria | Gammaproteobacteria | Alteromonadales | Ferrimonadaceae | Ferrimonas |  | 4 | 0.003 |
| Bacteria | Actinobacteria | Actinobacteria | Actinomycetales | Actinomycetaceae | Actinomyces |  | 4 | 0.003 |
| Bacteria | Tenericutes | Mollicutes | Acholeplasmatales | Acholeplasmataceae | Candidatus Phytoplasma | brasiliense | 4 | 0.003 |
| Bacteria | Bacteroidetes | Flavobacteriia | Flavobacteriales | Flavobacteriaceae | Salinimicrobium | terrae | 4 | 0.003 |
| Archaea | Euryarchaeota |  |  |  |  |  | 4 | 0.003 |
| Bacteria | Actinobacteria | Actinobacteria | Actinomycetales | Actinomycetaceae | Actinomyces | naturae | 4 | 0.003 |
| Bacteria | Thermotogae | Thermotogae | Thermotogales | Thermotogaceae |  |  | 4 | 0.003 |
| Bacteria | Proteobacteria | Gammaproteobacteria | Chromatiales | Chromatiaceae | Marichromatium | gracile | 4 | 0.003 |
| Bacteria | Thermotogae | Thermotogae | Thermotogales | Thermotogaceae | Fervidobacterium | islandicum | 4 | 0.003 |
| Bacteria | Proteobacteria | Deltaproteobacteria | Desulfovibrionales | Desulfovibrionaceae | Desulfovibrio | carbinoliphilus | 4 | 0.003 |
| Bacteria | Proteobacteria | Deltaproteobacteria | Desulfuromonadales | Geobacteraceae | Geobacter | uraniireducens | 4 | 0.003 |
| Bacteria | Proteobacteria | Gammaproteobacteria | Chromatiales | Ectothiorhodospiraceae | Methylonatrum | kenyense | 4 | 0.003 |
| Bacteria | Proteobacteria | Gammaproteobacteria | Alteromonadales | Shewanellaceae | Shewanella |  | 4 | 0.003 |
| Bacteria | Spirochaetes | Spirochaetes | Spirochaetales | Spirochaetaceae | Treponema | bryantii | 4 | 0.003 |
| Bacteria | Bacteroidetes | Bacteroidia | Bacteroidales | Bacteroidaceae | Bacteroides | intestinalis | 4 | 0.003 |
| Bacteria | Bacteroidetes | Sphingobacteriia | Sphingobacteriales | Sphingobacteriaceae | Sphingobacterium | bambusae | 4 | 0.003 |
| Bacteria | Cyanobacteria | Oscillatoriophycideae | Chroococcales | Microcystaceae | Microcystis | panniformis | 4 | 0.003 |
| Bacteria | Spirochaetes | Spirochaetes | Spirochaetales | Spirochaetaceae | Treponema | calligyrum | 4 | 0.003 |
| Bacteria | Firmicutes | Clostridia | Clostridiales | Peptostreptococcaceae | Clostridium | thermoalcaliphilum | 4 | 0.003 |
| Bacteria | Firmicutes | Clostridia | Clostridiales | Clostridiaceae | Caloramator | fervidus | 4 | 0.003 |
| Bacteria | Firmicutes | Clostridia | Thermoanaerobacterales |  |  |  | 4 | 0.003 |
| Bacteria | Cyanobacteria | Oscillatoriophycideae | Chroococcales |  |  |  | 4 | 0.003 |
| Bacteria | Bacteroidetes | Sphingobacteriia | Sphingobacteriales | Sphingobacteriaceae | Pedobacter | daejeonensis | 4 | 0.003 |
| Bacteria | Spirochaetes | Spirochaetes |  |  |  |  | 4 | 0.003 |
| Archaea | Euryarchaeota | Halobacteria | Halobacteriales | Halobacteriaceae | Haladaptatus |  | 3 | 0.003 |
| Bacteria | Firmicutes | Clostridia | Clostridiales | Clostridiaceae | Tindallia |  | 3 | 0.003 |
| Bacteria | Firmicutes | Clostridia | Desulfitobacterales | Desulfitobacteraceae | Desulfitobacter |  | 3 | 0.003 |
| Bacteria | Firmicutes | Clostridia | Clostridiales | Peptococcaceae | Desulfotomaculum | salinum | 3 | 0.003 |
| Bacteria | Thermodesulfobacteria | Thermodesulfobacteria | Thermodesulfobacteriales | Thermodesulfobacteriaceae | Thermodesulfatator | atlanticus | 3 | 0.003 |
| Bacteria | Actinobacteria | Actinobacteria | Actinomycetales | Microbacteriaceae | Agromyces |  | 3 | 0.003 |
| Bacteria | Actinobacteria | Actinobacteria | Actinomycetales | Pseudonocardiaceae | Saccharopolyspora |  | 3 | 0.003 |
| Bacteria | Actinobacteria | Actinobacteria | Actinomycetales | Actinomycetaceae | Actinobaculum | suis | 3 | 0.003 |
| Bacteria | Firmicutes | Clostridia | Clostridiales | Ruminococcaceae | Faecalibacterium |  | 3 | 0.003 |
| Bacteria | Firmicutes | Clostridia | Clostridiales | Lachnospiraceae | Blautia | hansenii | 3 | 0.003 |
| Bacteria | Firmicutes | Clostridia | Thermoanaerobacterales | Thermoanaerobacteraceae | Moorella | glycerini | 3 | 0.003 |
| Bacteria | Firmicutes | Clostridia | Thermoanaerobacterales | Thermoanaerobacteraceae | Thermoanaerobacter |  | 3 | 0.003 |
| Bacteria | Spirochaetes | Spirochaetes | Spirochaetales | Spirochaetaceae | Treponema | succinifaciens | 3 | 0.003 |
| Bacteria | Firmicutes | Bacilli | Bacillales | Paenibacillaceae | Cohnella |  | 3 | 0.003 |
| Bacteria | Proteobacteria | Alphaproteobacteria | Rhizobiales | Hyphomicrobiaceae | Rhodoplanes |  | 3 | 0.003 |
| Bacteria | Firmicutes | Bacilli | Lactobacillales | Leuconostocaceae | Weissella |  | 3 | 0.003 |
| Bacteria | Proteobacteria | Deltaproteobacteria | Desulfovibrionales | Desulfovibrionaceae | Desulfovibrio | butyratiphilus | 3 | 0.003 |
| Bacteria | Firmicutes | Clostridia | Coriobacteriales | Coriobacteriaceae | Eggerthella | sinensis | 3 | 0.003 |
| Bacteria | Bacteroidetes | Bacteroidia | Bacteroidales | Bacteroidaceae | Bacteroides | stercorirosoris | 3 | 0.003 |
| Bacteria | Proteobacteria | Deltaproteobacteria | Desulfovibrionales | Desulfovibrionaceae | Desulfovibrio | piger | 3 | 0.003 |
| Bacteria | Bacteroidetes | Bacteroidia | Bacteroidales | Porphyromonadaceae | Porphyromonas | macacae | 3 | 0.003 |
| Bacteria | Firmicutes | Clostridia | Clostridiales | Clostridiaceae | Peptoniphilus | gorbachii | 3 | 0.003 |
| Bacteria | Proteobacteria | Gammaproteobacteria | Pseudomonadales | Pseudomonadaceae | Pseudomonas |  | 3 | 0.003 |
| Bacteria | Firmicutes | Bacilli | Bacillales | Sporolactobacillaceae | Sporolactobacillus | putidus | 3 | 0.003 |
| Bacteria | Firmicutes | Clostridia | Clostridiales | Eubacteriaceae |  |  | 3 | 0.003 |
| Bacteria | Proteobacteria | Deltaproteobacteria | Syntrophobacterales | Desulfobacteraceae | Desulfococcus |  | 3 | 0.003 |
| Bacteria | Firmicutes | Clostridia | Clostridiales | Peptococcaceae | Dehalobacter | restrictus | 3 | 0.003 |
| Bacteria | Firmicutes | Clostridia | Clostridiales | Ruminococcaceae | Anaerofilum | pentosovorans | 3 | 0.003 |
| Bacteria | Nitrospirae | Nitrospira | Nitrospirales | Thermodesulfovibrionaceae | Thermodesulfovibrio | aggregans | 3 | 0.003 |
| Archaea | Euryarchaeota | Methanomicrobia |  |  |  |  | 3 | 0.003 |
| Bacteria | Proteobacteria | Betaproteobacteria | Burkholderiales | Oxalobacteraceae |  |  | 3 | 0.003 |
| Bacteria | Bacteroidetes | Sphingobacteriia | Sphingobacteriales | Sphingobacteriaceae | Parapedobacter | koreensis | 3 | 0.003 |
| Archaea | Euryarchaeota | Methanomicrobia | Methanomicrobiales | Methanomicrobiaceae | Methanofollis | ethanolicus | 3 | 0.003 |
| Bacteria | Firmicutes | Clostridia | Clostridiales | Symbiobacteriaceae | Symbiobacterium | toebii | 3 | 0.003 |
| Bacteria | Proteobacteria | Deltaproteobacteria | Myxococcales | Cystobacteraceae | Cystobacter |  | 3 | 0.003 |
| Archaea | Euryarchaeota | Methanomicrobia | Methanomicrobiales | Methanomicrobiaceae | Methanoculleus | submarinus | 3 | 0.003 |
| Bacteria | Firmicutes | Clostridia | Clostridiales | Peptococcaceae |  |  | 3 | 0.003 |
| Bacteria | Proteobacteria | Alphaproteobacteria | Rhodospirillales | Rhodospirillaceae | Azospirillum |  | 3 | 0.003 |
| Bacteria | Actinobacteria | Actinobacteria | Actinomycetales | Pseudonocardiaceae | Pseudonocardia |  | 3 | 0.003 |
| Bacteria | Acidobacteria | Acidobacteria | Acidobacteriales | Acidobacteriaceae |  |  | 3 | 0.003 |
| Bacteria | Firmicutes | Clostridia | Halanaerobiales | Halobacteroidaceae | Halanaerobacter | chitinivorans | 3 | 0.003 |
| Bacteria | Nitrospirae | Nitrospira | Nitrospirales | Thermodesulfovibrionaceae | Thermodesulfovibrio |  | 3 | 0.003 |
| Bacteria | Bacteroidetes | Bacteroidia | Bacteroidales | Odoribacteraceae | Butyricimonas | virosa | 3 | 0.003 |
| Archaea | Euryarchaeota | Methanomicrobia | Methanomicrobiales | Methanomicrobiaceae |  |  | 2 | 0.002 |
| Bacteria | Proteobacteria | Gammaproteobacteria | Xanthomonadales | Xanthomonadaceae |  |  | 2 | 0.002 |
| Bacteria | Proteobacteria | Gammaproteobacteria | Chromatiales |  |  |  | 2 | 0.002 |
| Bacteria | Firmicutes | Clostridia | Clostridiales | Clostridiaceae | Clostridium | acidisoli | 2 | 0.002 |
| Bacteria | Firmicutes | Clostridia | Clostridiales | Lachnospiraceae | Butyrivibrio | proteoclasticus | 2 | 0.002 |
| Bacteria | Proteobacteria | Betaproteobacteria | Neisseriales | Neisseriaceae | Chromobacterium | haemolyticum | 2 | 0.002 |
| Archaea | Euryarchaeota | Methanomicrobia | Methanomicrobiales | Methanomicrobiaceae | Methanoculleus | chikugoensis | 2 | 0.002 |
| Bacteria | Proteobacteria | Gammaproteobacteria | Enterobacteriales | Enterobacteriaceae | Enterobacter | hormaechei | 2 | 0.002 |
| Bacteria | Proteobacteria | Alphaproteobacteria | Rhodospirillales | Acetobacteraceae | Roseococcus | thiosulfatophilus | 2 | 0.002 |
| Bacteria | Firmicutes | Clostridia | Clostridiales | Peptococcaceae | Desulfotomaculum | thermoacetoxidans | 2 | 0.002 |
| Bacteria | Firmicutes | Bacilli | Lactobacillales | Lactobacillaceae | Lactobacillus | hilgardii | 2 | 0.002 |
| Bacteria | Actinobacteria | Actinobacteria | Actinomycetales | Streptomycetaceae | Streptomyces |  | 2 | 0.002 |
| Bacteria | Chlamydiae | Chlamydiia | Chlamydiales |  |  |  | 2 | 0.002 |
| Bacteria | Firmicutes | Clostridia | Thermoanaerobacterales | Thermoanaerobacteraceae | Ammonifex | thiophilus | 2 | 0.002 |
| Bacteria | Firmicutes | Clostridia | Coriobacteriales | Coriobacteriaceae | Slackia | piriformis | 2 | 0.002 |
| Bacteria | Actinobacteria | Nitriliruptoria | Euzebyales | Euzebyaceae | Euzebya | tangerina | 2 | 0.002 |
| Archaea | Euryarchaeota | Methanomicrobia | Methanosarcinales | Methanosarcinaceae | Methanosarcina |  | 2 | 0.002 |
| Archaea | Euryarchaeota | Methanobacteria | Methanobacteriales | Methanobacteriaceae | Methanobrevibacter | acididurans | 2 | 0.002 |
| Bacteria | Synergistetes | Synergistia | Synergistales | Dethiosulfovibrionaceae |  |  | 2 | 0.002 |
| Bacteria | Firmicutes | Clostridia | Clostridiales | Ruminococcaceae |  |  | 2 | 0.002 |
| Bacteria | Firmicutes | Clostridia | Clostridiales | Sulfobacillaceae | Sulfobacillus | yellowstonensis | 2 | 0.002 |
| Bacteria | Actinobacteria | Actinobacteria | Actinomycetales | Streptomycetaceae | Kitasatospora |  | 2 | 0.002 |
| Bacteria | Proteobacteria | Gammaproteobacteria | Xanthomonadales | Sinobacteraceae | Hydrocarboniphaga | daqingensis | 2 | 0.002 |
| Bacteria | Firmicutes | Clostridia | Clostridiales | Eubacteriaceae | Acetobacterium | tundrae | 2 | 0.002 |
| Bacteria | Firmicutes | Clostridia | Clostridiales | Clostridiaceae | Clostridium | malenominatum | 2 | 0.002 |
| Bacteria | Proteobacteria | Gammaproteobacteria | Enterobacteriales | Enterobacteriaceae | Proteus | hauseri | 2 | 0.002 |
| Bacteria | Proteobacteria | Deltaproteobacteria | Desulfovibrionales | Desulfonatronumaceae | Desulfonatronum | thiosulfatophilum | 2 | 0.002 |
| Bacteria | Synergistetes | Synergistia | Synergistales | Thermovirgaceae | Thermovirga | lienii | 2 | 0.002 |
| Bacteria | Proteobacteria | Deltaproteobacteria | Syntrophobacterales | Syntrophaceae |  |  | 2 | 0.002 |
| Bacteria | Cyanobacteria | Oscillatoriophycideae | Chroococcales | Gomphosphaeriaceae | Snowella | rosea | 2 | 0.002 |
| Bacteria | Bacteroidetes | Bacteroidia | Bacteroidales | Porphyromonadaceae | Parabacteroides | distasonis | 2 | 0.002 |
| Archaea | Euryarchaeota | Methanomicrobia | Methanosarcinales | Methanosarcinaceae | Methanosarcina | baltica | 2 | 0.002 |
| Bacteria | Tenericutes | Mollicutes | Mycoplasmatales | Mycoplasmataceae | Mycoplasma |  | 2 | 0.002 |
| Bacteria | Actinobacteria | Actinobacteria | Actinomycetales | Streptosporangiaceae | Streptosporangium |  | 2 | 0.002 |
| Archaea | Euryarchaeota | Methanomicrobia | Methanosarcinales | Methanosarcinaceae | Methanosalsum |  | 2 | 0.002 |
| Bacteria | Bacteroidetes | Flavobacteriia | Flavobacteriales | Flavobacteriaceae | Psychroflexus | gondwanensis | 2 | 0.002 |
| Bacteria | Proteobacteria | Gammaproteobacteria | Pseudomonadales | Moraxellaceae | Acinetobacter | tjernbergiae | 2 | 0.002 |
| Bacteria | Thermotogae | Thermotogae | Thermotogales | Thermotogaceae | Marinitoga |  | 2 | 0.002 |
| Bacteria | Firmicutes | Clostridia | Clostridiales | Clostridiaceae | Mogibacterium |  | 2 | 0.002 |
| Bacteria | Proteobacteria | Alphaproteobacteria | Rhodospirillales | Rhodospirillaceae | Rhodospirillum |  | 2 | 0.002 |
| Bacteria | Firmicutes | Bacilli | Lactobacillales | Lactobacillaceae | Lactobacillus |  | 2 | 0.002 |
| Bacteria | Bacteroidetes | Flavobacteriia | Flavobacteriales | Flavobacteriaceae | Zhouia | amylolytica | 2 | 0.002 |
| Bacteria | Actinobacteria |  |  |  |  |  | 2 | 0.002 |
| Bacteria | Proteobacteria | Deltaproteobacteria | Desulfuromonadales | Desulfuromonadaceae |  |  | 2 | 0.002 |
| Bacteria | Proteobacteria | Epsilonproteobacteria | Campylobacterales | Campylobacteraceae | Campylobacter |  | 2 | 0.002 |
| Bacteria | Proteobacteria | Alphaproteobacteria | Rhodospirillales | Acetobacteraceae | Acetobacter |  | 2 | 0.002 |
| Bacteria | Proteobacteria | Gammaproteobacteria | Chromatiales | Chromatiaceae | Thiorhodococcus | pfennigii | 2 | 0.002 |
| Bacteria | Proteobacteria | Alphaproteobacteria | Rhodobacterales | Rhodobacteraceae |  |  | 2 | 0.002 |
| Bacteria | Firmicutes | Clostridia | Clostridiales | Peptococcaceae | Desulfitobacterium | chlororespirans | 2 | 0.002 |
| Bacteria | Proteobacteria | Gammaproteobacteria | Chromatiales | Chromatiaceae |  |  | 2 | 0.002 |
| Archaea | Euryarchaeota | Methanobacteria | Methanobacteriales | Methanobacteriaceae |  |  | 2 | 0.002 |
| Archaea | Crenarchaeota | Thermoprotei | Desulfurococcales | Desulfurococcaceae | Caldisphaera | draconis | 2 | 0.002 |
| Bacteria | Firmicutes | Clostridia | Clostridiales | Veillonellaceae | Phascolarctobacterium |  | 2 | 0.002 |
| Bacteria | Actinobacteria | Actinobacteria | Actinomycetales | Micromonosporaceae | Micromonospora | rifamycinica | 2 | 0.002 |
| Bacteria | Proteobacteria | Alphaproteobacteria | Rhodospirillales | Rhodospirillaceae | Roseospira |  | 2 | 0.002 |
| Bacteria | Firmicutes | Bacilli | Lactobacillales | Lactobacillaceae | Pediococcus |  | 2 | 0.002 |
| Bacteria | Firmicutes | Bacilli | Bacillales | Planococcaceae | Planococcus | maritimus | 1 | 0.001 |
| Bacteria | Proteobacteria | Alphaproteobacteria | Rhodobacterales | Rhodobacteraceae | Anaerospora |  | 1 | 0.001 |
| Bacteria | Actinobacteria | Thermoleophilia | Solirubrobacterales |  |  |  | 1 | 0.001 |
| Bacteria | Actinobacteria | Actinobacteria | Actinomycetales | Pseudonocardiaceae | Amycolatopsis | methanolica | 1 | 0.001 |
| Bacteria | Firmicutes | Clostridia | Clostridiales | Clostridiaceae | Clostridium | caliptrosporum | 1 | 0.001 |
| Bacteria | Proteobacteria | Betaproteobacteria | Burkholderiales | Alcaligenaceae | Tetrathiobacter | kashmirensis | 1 | 0.001 |
| Bacteria | Thermotogae | Thermotogae | Thermotogales | Thermotogaceae | Marinitoga | hydrogenitolerans | 1 | 0.001 |
| Bacteria | Proteobacteria | Alphaproteobacteria | Rhizobiales | Rhizobiaceae | Candidatus Liberibacter | africanus | 1 | 0.001 |
| Bacteria | Proteobacteria | Gammaproteobacteria | Thiohalorhabdales | Thiohalorhabdaceae | Thiohalorhabdus | denitrificans | 1 | 0.001 |
| Bacteria | Proteobacteria | Gammaproteobacteria | Xanthomonadales | Xanthomonadaceae | Luteimonas |  | 1 | 0.001 |
| Bacteria | Tenericutes | Mollicutes | Acholeplasmatales | Acholeplasmataceae | Candidatus Phytoplasma | prunorum | 1 | 0.001 |
| Bacteria | Actinobacteria | Actinobacteria | Actinomycetales | Actinomycetaceae | Arcanobacterium |  | 1 | 0.001 |
| Bacteria | Firmicutes | Clostridia | Clostridiales | Clostridiaceae | Clostridium | akagii | 1 | 0.001 |
| Bacteria | Firmicutes | Clostridia | Clostridiales | Veillonellaceae | Mitsuokella |  | 1 | 0.001 |
| Bacteria | Proteobacteria | Gammaproteobacteria | Thiotrichales | Thiotrichaceae | Leucothrix | mucor | 1 | 0.001 |
| Bacteria | Firmicutes | Clostridia | Clostridiales | Peptococcaceae | Desulfotomaculum | carboxydivorans | 1 | 0.001 |
| Bacteria | Bacteroidetes | Bacteroidia | Bacteroidales | Porphyromonadaceae | Porphyromonas | circumdentaria | 1 | 0.001 |
| Bacteria | Chlorobi | Chlorobia | Chlorobiales | Chlorobiaceae | Chlorobaculum |  | 1 | 0.001 |
| Bacteria | Firmicutes | Bacilli | Bacillales | Staphylococcaceae | Staphylococcus | massiliensis | 1 | 0.001 |
| Bacteria | Actinobacteria | Actinobacteria | Actinomycetales | Nocardioidaceae | Kribbella | ginsengisoli | 1 | 0.001 |
| Bacteria | Firmicutes | Clostridia | Coriobacteriales | Coriobacteriaceae | Atopobium | minutum | 1 | 0.001 |
| Bacteria | Proteobacteria | Deltaproteobacteria | Desulfuromonadales | Geobacteraceae | Geobacter | pelophilus | 1 | 0.001 |
| Bacteria | Proteobacteria | Gammaproteobacteria | Pseudomonadales | Moraxellaceae | Acinetobacter | johnsonii | 1 | 0.001 |
| Bacteria | Proteobacteria | Alphaproteobacteria | Rhizobiales | Rhizobiaceae | Candidatus Liberibacter |  | 1 | 0.001 |
| Bacteria | Firmicutes | Clostridia | Clostridiales | Clostridiaceae | Sarcina | maxima | 1 | 0.001 |
| Archaea | Euryarchaeota | Methanomicrobia | Methanomicrobiales | Methanocorpusculaceae |  |  | 1 | 0.001 |
| Bacteria | Firmicutes | Bacilli | Lactobacillales | Streptococcaceae | Lactococcus |  | 1 | 0.001 |
| Bacteria | Verrucomicrobia | Opitutae | Puniceicoccales | Puniceicoccaceae | Coraliomargarita | akajimensis | 1 | 0.001 |
| Bacteria | Firmicutes | Bacilli | Lactobacillales | Enterococcaceae | Enterococcus | aquimarinus | 1 | 0.001 |
| Bacteria | Firmicutes | Bacilli | Bacillales | Bacillaceae | Marinococcus |  | 1 | 0.001 |
| Bacteria | Firmicutes | Bacilli | Bacillales | Paenibacillaceae | Cohnella | laeviribosi | 1 | 0.001 |
| Bacteria | Actinobacteria | Actinobacteria | Actinomycetales | Microbacteriaceae | Leucobacter |  | 1 | 0.001 |
| Bacteria | Proteobacteria | Gammaproteobacteria | Oceanospirillales | Halomonadaceae | Kushneria | indalinina | 1 | 0.001 |
| Bacteria | Proteobacteria | Epsilonproteobacteria | Campylobacterales | Helicobacteraceae | Helicobacter | suncus | 1 | 0.001 |
| Archaea | Euryarchaeota | Methanomicrobia | Methanomicrobiales | Methanomicrobiaceae | Methanogenium |  | 1 | 0.001 |
| Bacteria | Proteobacteria | Gammaproteobacteria | Pasteurellales | Pasteurellaceae |  |  | 1 | 0.001 |
| Bacteria | Proteobacteria | Alphaproteobacteria | Rhizobiales | Xanthobacteraceae | Xanthobacter |  | 1 | 0.001 |
| Bacteria | Actinobacteria | Actinobacteria | Actinomycetales | Nocardioidaceae | Actinopolymorpha |  | 1 | 0.001 |
| Bacteria | Proteobacteria | Gammaproteobacteria | Oceanospirillales | Litoricolaceae | Litoricola |  | 1 | 0.001 |
| Bacteria | Proteobacteria | Gammaproteobacteria | Pseudomonadales | Moraxellaceae | Acinetobacter |  | 1 | 0.001 |
| Bacteria | Firmicutes | Clostridia | Halanaerobiales | Halobacteroidaceae | Natroniella |  | 1 | 0.001 |
| Bacteria | Firmicutes | Bacilli | Bacillales | Paenibacillaceae | Brevibacillus | ginsengisoli | 1 | 0.001 |
| Bacteria | Proteobacteria | Gammaproteobacteria | Legionellales | Legionellaceae | Legionella |  | 1 | 0.001 |
| Bacteria | Firmicutes | Bacilli | Bacillales | Staphylococcaceae |  |  | 1 | 0.001 |
| Bacteria | Firmicutes | Clostridia | Clostridiales | Lachnospiraceae | Lachnobacterium |  | 1 | 0.001 |
| Bacteria | Proteobacteria | Deltaproteobacteria | Desulfobacterales | Desulfobulbaceae | Desulfotalea | arctica | 1 | 0.001 |
| Bacteria | Proteobacteria | Alphaproteobacteria | Sphingomonadales | Sphingomonadaceae | Sphingobium | amiense | 1 | 0.001 |
| Bacteria | Proteobacteria | Deltaproteobacteria | Desulfovibrionales | Desulfovibrionaceae | Desulfovibrio | idahonensis | 1 | 0.001 |
| Bacteria | Synergistetes | Synergistia | Synergistales | Anaerobaculaceae | Anaerobaculum | thermoterrnum | 1 | 0.001 |
| Bacteria | Proteobacteria | Epsilonproteobacteria | Campylobacterales | Campylobacteraceae | Sulfurospirillum | deleyianum | 1 | 0.001 |
| Bacteria | Proteobacteria | Deltaproteobacteria | Myxococcales |  |  |  | 1 | 0.001 |
| Bacteria | Proteobacteria | Gammaproteobacteria | Pseudomonadales | Pseudomonadaceae | Pseudomonas | guineae | 1 | 0.001 |
| Bacteria | Firmicutes | Bacilli | Bacillales | Paenibacillaceae | Paenibacillus | contaminans | 1 | 0.001 |
| Bacteria | Firmicutes | Clostridia | Clostridiales | Syntrophomonadaceae | Syntrophomonas | erecta | 1 | 0.001 |
| Bacteria | Firmicutes | Bacilli | Lactobacillales | Leuconostocaceae | Leuconostoc | mesenteroides | 1 | 0.001 |
| Bacteria | Firmicutes | Clostridia | Clostridiales | Eubacteriaceae | Pseudoramibacter |  | 1 | 0.001 |
| Bacteria | Firmicutes | Clostridia | Clostridiales | Lachnospiraceae | Coprococcus | catus | 1 | 0.001 |
| Bacteria | Cyanobacteria | Oscillatoriophycideae | Chroococcales | Phormidiaceae | Oscillatoria | corallinae | 1 | 0.001 |
| Bacteria | Firmicutes | Bacilli | Lactobacillales | Leuconostocaceae | Oenococcus |  | 1 | 0.001 |
| Bacteria | Deferribacteres | Deferribacteres | Deferribacterales | Deferribacteraceae | Deferribacter | autotrophicus | 1 | 0.001 |
| Bacteria | Proteobacteria | Alphaproteobacteria | Rhizobiales | Bartonellaceae | Bartonella |  | 1 | 0.001 |
| Bacteria | Firmicutes | Clostridia | Clostridiales | Lachnospiraceae | Oribacterium | sinus | 1 | 0.001 |
| Bacteria | Actinobacteria | Actinobacteria | Bifidobacteriales | Bifidobacteriaceae | Bifidobacterium | gallinarum | 1 | 0.001 |
| Bacteria | Firmicutes | Clostridia | Clostridiales | Clostridiaceae | Clostridium | ganghwense | 1 | 0.001 |
| Bacteria | Chlamydiae | Chlamydiia | Chlamydiales | Waddliaceae | Waddlia |  | 1 | 0.001 |
| Bacteria | Proteobacteria | Alphaproteobacteria | Rhizobiales | Hyphomicrobiaceae | Pedomicrobium | australicum | 1 | 0.001 |
| Bacteria | Proteobacteria | Gammaproteobacteria | Oceanospirillales | Halomonadaceae |  |  | 1 | 0.001 |
| Bacteria | Proteobacteria | Deltaproteobacteria | Desulfuromonadales | Pelobacteraceae | Pelobacter | carbinolicus | 1 | 0.001 |
| Bacteria | Bacteroidetes | Sphingobacteriia | Sphingobacteriales | Saprospiraceae | Lewinella | lutea | 1 | 0.001 |
| Bacteria | Actinobacteria | Actinobacteria | Actinomycetales | Pseudonocardiaceae | Amycolatopsis | helveola | 1 | 0.001 |
| Bacteria | Proteobacteria | Gammaproteobacteria | Enterobacteriales | Enterobacteriaceae | Enterobacter | aerogenes | 1 | 0.001 |
| Bacteria | Proteobacteria | Alphaproteobacteria | Rhodobacterales | Hyphomonadaceae | Maricaulis | indicus | 1 | 0.001 |
| Bacteria | Proteobacteria | Alphaproteobacteria | Rickettsiales | Rickettsiaceae | Rickettsia | marmionii | 1 | 0.001 |
| Bacteria | Cyanobacteria | Nostocophycideae | Nostocales | Nostocaceae | Nostoc | piscinale | 1 | 0.001 |
| Bacteria | Firmicutes | Clostridia | Clostridiales | Clostridiaceae | Clostridium | cavendishii | 1 | 0.001 |
| Bacteria | Bacteroidetes | Bacteroidia | Bacteroidales | Porphyromonadaceae | Porphyromonas | somerae | 1 | 0.001 |
| Bacteria | Proteobacteria | Deltaproteobacteria | Bdellovibrionales | Bdellovibrionaceae | Bdellovibrio | exovorus | 1 | 0.001 |
| Bacteria | Cyanobacteria | Nostocophycideae | Nostocales | Nostocaceae | Dolichospermum |  | 1 | 0.001 |
| Bacteria | Firmicutes | Bacilli | Bacillales | Bacillaceae | Bacillus | infantis | 1 | 0.001 |
| Bacteria | Actinobacteria | Actinobacteria | Actinomycetales | Mycobacteriaceae | Mycobacterium | coloregonium | 1 | 0.001 |
| Archaea | Euryarchaeota | Methanomicrobia | Methanosarcinales | Methanosarcinaceae | Methanosarcina | barkeri | 1 | 0.001 |
| Bacteria | Actinobacteria | Actinobacteria | Actinomycetales | Nocardiaceae | Rhodococcus | percolatus | 1 | 0.001 |
| Bacteria | Bacteroidetes | Bacteroidia | Bacteroidales | Porphyromonadaceae | Porphyromonas |  | 1 | 0.001 |
| Bacteria | Actinobacteria | Actinobacteria | Actinomycetales | Actinomycetaceae | Actinomyces | turicensis | 1 | 0.001 |
| Bacteria | Proteobacteria | Deltaproteobacteria | Desulfovibrionales | Desulfovibrionaceae | Desulfovibrio | salexigens | 1 | 0.001 |
| Bacteria | Proteobacteria | Deltaproteobacteria | Syntrophobacterales | Desulfobacteraceae | Desulfosarcina | cetonica | 1 | 0.001 |
| Bacteria | Cyanobacteria | Nostocophycideae | Stigonematales | Rivulariaceae | Calothrix |  | 1 | 0.001 |
| Archaea | Euryarchaeota | Halobacteria | Halobacteriales | Halobacteriaceae | Haloferax |  | 1 | 0.001 |
| Bacteria | Actinobacteria | Actinobacteria | Actinomycetales | Kineosporiaceae | Kineosporia | rhizophila | 1 | 0.001 |
| Bacteria | Firmicutes | Clostridia | Clostridiales | Eubacteriaceae | Anaerofustis |  | 1 | 0.001 |
| Bacteria | Bacteroidetes | Sphingobacteriia | Sphingobacteriales | Flexibacteraceae | Hymenobacter |  | 1 | 0.001 |
| Bacteria | Bacteroidetes | Bacteroidia | Bacteroidales | Prevotellaceae | Prevotella | bivia | 1 | 0.001 |
| Bacteria | Proteobacteria | Gammaproteobacteria | Vibrionales | Vibrionaceae | Vibrio | litoralis | 1 | 0.001 |
| Bacteria | Firmicutes | Clostridia | Clostridiales | Peptococcaceae | Desulfosporosinus | hippei | 1 | 0.001 |
| Bacteria | Thermi | Deinococci | Deinococcales | Deinococcaceae | Deinococcus |  | 1 | 0.001 |
| Bacteria | Proteobacteria | Gammaproteobacteria | Enterobacteriales | Enterobacteriaceae | Klebsiella |  | 1 | 0.001 |
| Bacteria | Proteobacteria | Deltaproteobacteria | Syntrophobacterales | Desulfobacteraceae | Desulfofaba |  | 1 | 0.001 |
| Bacteria | Firmicutes | Clostridia | Coriobacteriales | Coriobacteriaceae | Atopobium | fossor | 1 | 0.001 |
| Bacteria | Firmicutes | Clostridia | Coriobacteriales | Coriobacteriaceae |  |  | 1 | 0.001 |
| Bacteria | Bacteroidetes | Bacteroidia | Bacteroidales | Bacteroidaceae | Bacteroides | rodentium | 1 | 0.001 |
| Bacteria | Proteobacteria | Gammaproteobacteria | Enterobacteriales | Enterobacteriaceae | Proteus |  | 1 | 0.001 |
| Bacteria | Firmicutes | Bacilli | Bacillales | Staphylococcaceae | Salinicoccus | iranensis | 1 | 0.001 |
| Bacteria | Chloroflexi | Anaerolineae | Anaerolineales | Anaerolinaceae | Anaerolinea | thermolimosa | 1 | 0.001 |
| Bacteria | Firmicutes | Clostridia | Clostridiales | Clostridiaceae | Soehngenia | saccharolytica | 1 | 0.001 |
| Bacteria | Firmicutes | Clostridia | Clostridiales | Clostridiaceae | Clostridium | aestuarii | 1 | 0.001 |
| Bacteria | Firmicutes | Clostridia | Clostridiales | Peptococcaceae | Desulfotomaculum | halophilum | 1 | 0.001 |
| Bacteria | Actinobacteria | Rubrobacteria | Rubrobacterales | Rubrobacteraceae | Rubrobacter | xylanophilus | 1 | 0.001 |
| Bacteria | Thermotogae | Thermotogae | Thermotogales | Thermotogaceae | Thermosipho | ferriphilus | 1 | 0.001 |
| Bacteria | Cyanobacteria | Nostocophycideae | Nostocales | Nostocaceae | Nostoc |  | 1 | 0.001 |
| Bacteria | Proteobacteria | Betaproteobacteria | Burkholderiales | Comamonadaceae |  |  | 1 | 0.001 |
| Bacteria | Chloroflexi | Ktedonobacteria | Thermogemmatisporales | Thermogemmatisporaceae | Thermogemmatispora |  | 1 | 0.001 |
| Bacteria | Firmicutes | Bacilli | Bacillales | Bacillaceae | Anoxybacillus |  | 1 | 0.001 |
| Bacteria | Chlorobi | Chlorobia | Chlorobiales | Chlorobiaceae | Chlorobaculum | limnaeum | 1 | 0.001 |
| Bacteria | Proteobacteria | Deltaproteobacteria | Desulfovibrionales | Desulfovibrionaceae | Desulfovibrio | aceae | 1 | 0.001 |
| Bacteria | Bacteroidetes | Sphingobacteriia | Sphingobacteriales | Flexibacteraceae | Dyadobacter |  | 1 | 0.001 |
| Bacteria | Proteobacteria | Alphaproteobacteria | Rhodobacterales | Rhodobacteraceae | Rhodobacter |  | 1 | 0.001 |
| Bacteria | Actinobacteria | Actinobacteria | Actinomycetales | Mycobacteriaceae | Mycobacterium |  | 1 | 0.001 |
| Bacteria | Firmicutes | Erysipelotrichi | Erysipelotrichales | Erysipelotrichaceae |  |  | 1 | 0.001 |
| Bacteria | Firmicutes | Clostridia | Clostridiales | Clostridiaceae | Clostridium | magnum | 1 | 0.001 |
| Bacteria | Firmicutes | Bacilli | Turicibacterales | Turicibacteraceae | Turicibacter |  | 1 | 0.001 |
| Bacteria | Proteobacteria | Gammaproteobacteria | Thiotrichales | Thiotrichaceae | Thiothrix | nivea | 1 | 0.001 |
| Bacteria | Cyanobacteria | Synechococcophycideae | Pseudanabaenales | Pseudanabaenaceae |  |  | 1 | 0.001 |
| Bacteria | Proteobacteria | Gammaproteobacteria | Vibrionales | Vibrionaceae | Salinivibrio | budaii | 1 | 0.001 |
| Bacteria | Proteobacteria | Alphaproteobacteria | Rhizobiales | Methylocystaceae | Pleomorphomonas |  | 1 | 0.001 |
| Bacteria | Firmicutes | Clostridia | Thermoanaerobacterales | Thermoanaerobacteraceae | Thermoanaerobacter | kivui | 1 | 0.001 |
| Bacteria | Proteobacteria | Alphaproteobacteria | Rhodobacterales | Rhodobacteraceae | Paracoccus | homiensis | 1 | 0.001 |
| Bacteria | Firmicutes | Clostridia | Clostridiales | Clostridiaceae | Peptoniphilus | methioninivorax | 1 | 0.001 |
| Bacteria | Firmicutes | Erysipelotrichi | Erysipelotrichales | Erysipelotrichaceae | Holdemania |  | 1 | 0.001 |
| Bacteria | Tenericutes | Mollicutes | Mycoplasmatales | Mycoplasmataceae | Mycoplasma | edwardii | 1 | 0.001 |
| Bacteria | Firmicutes | Bacilli | Bacillales | Planococcaceae | Lysinibacillus | parviboronicapiens | 1 | 0.001 |
| Bacteria | Proteobacteria | Gammaproteobacteria | Oceanospirillales | Litoricolaceae | Litoricola | lipolytica | 1 | 0.001 |
| Bacteria | Proteobacteria | Alphaproteobacteria | Rhodospirillales | Rhodospirillaceae | Telmatospirillum |  | 1 | 0.001 |
| Bacteria | Proteobacteria | Alphaproteobacteria | Rhodospirillales | Acetobacteraceae | Swaminathania | salitolerans | 1 | 0.001 |
| Bacteria | Proteobacteria | Alphaproteobacteria | Rhodospirillales | Acetobacteraceae | Roseomonas |  | 1 | 0.001 |
| Bacteria | Proteobacteria | Gammaproteobacteria | Oceanospirillales | Halomonadaceae | Halomonas |  | 1 | 0.001 |
| Bacteria | Actinobacteria | Actinobacteria | Actinomycetales | Glycomycetaceae | Glycomyces |  | 1 | 0.001 |
| Bacteria | Firmicutes | Bacilli | Lactobacillales | Enterococcaceae | Enterococcus |  | 1 | 0.001 |
| Bacteria | Proteobacteria | Alphaproteobacteria | Rickettsiales | Anaplasmataceae | Ehrlichia | ovina | 1 | 0.001 |
| Archaea | Euryarchaeota | Thermococci | Thermococcales | Thermococcaceae | Pyrococcus | yayanosii | 1 | 0.001 |
| Bacteria | Proteobacteria | Deltaproteobacteria | Desulfovibrionales | Desulfovibrionaceae | Desulfovibrio | tunisiensis | 1 | 0.001 |
| Bacteria | Proteobacteria | Gammaproteobacteria | Legionellales | Francisellaceae | Francisella | hispaniensis | 1 | 0.001 |
| Bacteria | Firmicutes | Clostridia | Clostridiales | Clostridiaceae | Caloramator | viterbiensis | 1 | 0.001 |
| Bacteria | Firmicutes | Bacilli | Bacillales | Paenibacillaceae | Cohnella | soli | 1 | 0.001 |
| Bacteria | Proteobacteria | Gammaproteobacteria | Enterobacteriales | Enterobacteriaceae | Providencia | rettgeri | 1 | 0.001 |
