# Supplementary material for: Methane-yielding microbial communities processing lactate-rich substrates: a piece of the anaerobic digestion puzzle
Source: Biotechnol Biofuels. 2018 Apr 21;11:116. doi: 10.1186/s13068-018-1106-z (PMC5910564; doi:10.1186/s13068-018-1106-z)
Supplement: Supplementary file 4 — Additional file 4. Number of reads assigned to respective taxonomic branches, M1 B microbial community. [file 13068_2018_1106_MOESM4_ESM.docx]

Additional file 4. Number of reads assigned to respective taxonomic branches, M1 B microbial community.

| Kingdom | Phylum | Class | Order | Family | Genus | Species | num_hits | %_hits |
| --- | --- | --- | --- | --- | --- | --- | --- | --- |
| Unclassified |  |  |  |  |  |  | 5469 | 5.149 |
| Archaea | Euryarchaeota | Methanomicrobia | Methanosarcinales | Methanosaetaceae | Methanosaeta | concilii | 27017 | 25.437 |
| Bacteria | Proteobacteria | Deltaproteobacteria | Syntrophobacterales | Syntrophaceae | Syntrophus |  | 14483 | 13.636 |
| Bacteria | Bacteroidetes | Sphingobacteriia | Sphingobacteriales | Sphingobacteriaceae | Pedobacter | kwangyangensis | 5597 | 5.27 |
| Bacteria | Spirochaetes | Spirochaetes | Spirochaetales | Spirochaetaceae | Treponema |  | 4300 | 4.048 |
| Bacteria |  |  |  |  |  |  | 3403 | 3.204 |
| Bacteria | Synergistetes | Synergistia | Synergistales | Synergistaceae | Candidatus Tammella | caduceiae | 3121 | 2.938 |
| Archaea | Euryarchaeota | Methanomicrobia | Methanomicrobiales | Methanomicrobiaceae | Methanoculleus |  | 3082 | 2.902 |
| Bacteria | Synergistetes | Synergistia | Synergistales | Aminiphilaceae | Aminiphilus | circumscriptus | 2145 | 2.02 |
| Bacteria | Firmicutes | Clostridia | Clostridiales | Syntrophomonadaceae | Syntrophomonas |  | 1920 | 1.808 |
| Archaea | Euryarchaeota | Methanomicrobia | Methanomicrobiales | Methanocorpusculaceae | Methanocorpusculum | labreanum | 1513 | 1.424 |
| Bacteria | Firmicutes | Clostridia | Thermoanaerobacterales | Thermovenabulum | Thermovenabulum | ferriorganovorum | 1320 | 1.243 |
| Bacteria | Proteobacteria | Deltaproteobacteria | Syntrophobacterales | Desulfobacteraceae |  |  | 1106 | 1.041 |
| Bacteria | Firmicutes | Clostridia | Clostridiales | Sulfobacillaceae | Sulfobacillus | yellowstonensis | 1075 | 1.012 |
| Bacteria | Caldithrix | Caldithrixae | Caldithrixales | Caldithrixaceae | Caldithrix |  | 1061 | 0.999 |
| Bacteria | Proteobacteria |  |  |  |  |  | 1001 | 0.942 |
| Bacteria | Synergistetes | Synergistia | Synergistales | Synergistaceae | Synergistes |  | 967 | 0.91 |
| Bacteria | Bacteroidetes | Flavobacteriia | Flavobacteriales | Flavobacteriaceae |  |  | 946 | 0.891 |
| Archaea | Euryarchaeota | Methanobacteria | Methanobacteriales | Methanobacteriaceae | Methanobacterium |  | 873 | 0.822 |
| Bacteria | Firmicutes | Clostridia | Clostridiales | Clostridiaceae | Clostridium |  | 861 | 0.811 |
| Bacteria | Cyanobacteria | Nostocophycideae | Nostocales | Nostocaceae |  |  | 802 | 0.755 |
| Bacteria | Verrucomicrobia | Spartobacteria | Chthoniobacterales | Chthoniobacteraceae | Chthoniobacter | flavus | 791 | 0.745 |
| Archaea | Euryarchaeota | Methanomicrobia | Methanomicrobiales | Methanomicrobiaceae | Methanoculleus | receptaculi | 707 | 0.666 |
| Bacteria | Firmicutes | Clostridia | Clostridiales | Veillonellaceae | Megasphaera | paucivorans | 707 | 0.666 |
| Bacteria | Chloroflexi | Anaerolineae | Anaerolineales | Anaerolinaceae | Longilinea | arvoryzae | 704 | 0.663 |
| Bacteria | Synergistetes | Synergistia | Synergistales | Dethiosulfovibrionaceae | Dethiosulfovibrio |  | 664 | 0.625 |
| Bacteria | Bacteroidetes | Sphingobacteriia | Sphingobacteriales | Sphingobacteriaceae | Sphingobacterium | shayense | 544 | 0.512 |
| Bacteria | Thermotogae | Thermotogae | Thermotogales | Thermotogaceae | Kosmotoga |  | 535 | 0.504 |
| Bacteria | Bacteroidetes | Flavobacteriia | Flavobacteriales | Flavobacteriaceae | Myroides |  | 532 | 0.501 |
| Bacteria | Firmicutes | Clostridia | Clostridiales |  |  |  | 523 | 0.492 |
| Archaea | Euryarchaeota | Methanomicrobia | Methanosarcinales | Methanosaetaceae | Methanosaeta |  | 519 | 0.489 |
| Bacteria | Chloroflexi | Anaerolineae | Anaerolineales | Anaerolinaceae | Bellilinea | caldifistulae | 499 | 0.47 |
| Bacteria | Bacteroidetes |  |  |  |  |  | 498 | 0.469 |
| Bacteria | Bacteroidetes | Bacteroidia | Bacteroidales | Bacteroidaceae | Bacteroides | denticanum | 481 | 0.453 |
| Bacteria | Bacteroidetes | Sphingobacteriia | Sphingobacteriales | Sphingobacteriaceae | Pedobacter |  | 478 | 0.45 |
| Bacteria | Firmicutes | Clostridia | Clostridiales | Syntrophomonadaceae | Syntrophomonas | cellicola | 451 | 0.425 |
| Bacteria | Firmicutes | Clostridia | Thermoanaerobacterales | Caldicellulosiruptoraceae | Caldicellulosiruptor |  | 445 | 0.419 |
| Bacteria | Firmicutes | Clostridia | Clostridiales | Clostridiaceae | Clostridium | caenicola | 444 | 0.418 |
| Bacteria | Firmicutes | Clostridia | Clostridiales | Syntrophomonadaceae | Syntrophomonas | wolfei | 436 | 0.41 |
| Bacteria | Verrucomicrobia |  |  |  |  |  | 434 | 0.409 |
| Bacteria | Proteobacteria | Deltaproteobacteria | Desulfovibrionales | Desulfovibrionaceae | Desulfovibrio | simplex | 400 | 0.377 |
| Bacteria | Synergistetes | Synergistia | Synergistales | Dethiosulfovibrionaceae | Pyramidobacter |  | 373 | 0.351 |
| Bacteria | Firmicutes | Clostridia |  |  |  |  | 369 | 0.347 |
| Bacteria | Thermotogae | Thermotogae | Thermotogales | Thermotogaceae | Marinitoga |  | 365 | 0.344 |
| Bacteria | Firmicutes | Bacilli | Thermicanales | Thermicanaceae | Thermicanus |  | 358 | 0.337 |
| Bacteria | Proteobacteria | Deltaproteobacteria | Syntrophobacterales | Syntrophaceae | Desulfomonile | tiedjei | 353 | 0.332 |
| Bacteria | Proteobacteria | Alphaproteobacteria | Rhodobacterales |  |  |  | 348 | 0.328 |
| Bacteria | Firmicutes | Clostridia | Clostridiales | Clostridiaceae | Alkaliphilus |  | 322 | 0.303 |
| Bacteria | Proteobacteria | Deltaproteobacteria | Desulfovibrionales | Desulfohalobiaceae | Desulfonauticus | submarinus | 309 | 0.291 |
| Bacteria | Proteobacteria | Deltaproteobacteria | Desulfovibrionales | Desulfovibrionaceae | Desulfovibrio | intestinalis | 300 | 0.282 |
| Bacteria | Firmicutes | Clostridia | Clostridiales | Peptococcaceae | Desulfurispora | thermophila | 299 | 0.282 |
| Bacteria | Firmicutes | Clostridia | Clostridiales | Clostridiaceae | Caloramator | mitchellensis | 295 | 0.278 |
| Bacteria | Bacteroidetes | Flavobacteriia | Flavobacteriales | Flavobacteriaceae | Chryseobacterium |  | 288 | 0.271 |
| Bacteria | Proteobacteria | Deltaproteobacteria | Desulfovibrionales | Desulfovibrionaceae | Desulfovibrio |  | 283 | 0.266 |
| Bacteria | Cyanobacteria |  |  |  |  |  | 280 | 0.264 |
| Bacteria | Bacteroidetes | Bacteroidia | Bacteroidales |  |  |  | 277 | 0.261 |
| Bacteria | Firmicutes | Clostridia | Clostridiales | Syntrophomonadaceae | Syntrophomonas | palmitatica | 273 | 0.257 |
| Bacteria | Spirochaetes | Spirochaetes | Spirochaetales | Spirochaetaceae | Treponema | brennaborense | 258 | 0.243 |
| Bacteria | Firmicutes | Clostridia | Clostridiales | Veillonellaceae | Megasphaera |  | 247 | 0.233 |
| Bacteria | Synergistetes | Synergistia | Synergistales | Dethiosulfovibrionaceae | Aminobacterium |  | 234 | 0.22 |
| Bacteria | Firmicutes | Clostridia | Clostridiales | Veillonellaceae | Anaeromusa | acidaminophila | 232 | 0.218 |
| Bacteria | Synergistetes | Synergistia | Synergistales |  |  |  | 226 | 0.213 |
| Bacteria | Firmicutes | Clostridia | Clostridiales | Carboxydocellaceae | Carboxydocella | ferrireducens | 219 | 0.206 |
| Bacteria | Cyanobacteria | Synechococcophycideae | Pseudanabaenales | Pseudanabaenaceae | Leptolyngbya | laminosa | 215 | 0.202 |
| Archaea | Euryarchaeota | Methanomicrobia | Methanomicrobiales | Methanomicrobiaceae | Methanoculleus | palmolei | 211 | 0.199 |
| Bacteria | Firmicutes | Clostridia | Clostridiales | Veillonellaceae | Megasphaera | hominis | 210 | 0.198 |
| Bacteria | Firmicutes |  |  |  |  |  | 209 | 0.197 |
| Bacteria | Synergistetes | Synergistia | Synergistales | Synergistaceae | Thermococcus |  | 199 | 0.187 |
| Bacteria | Synergistetes | Synergistia | Synergistales | Synergistaceae | Cloacibacillus |  | 199 | 0.187 |
| Bacteria | Synergistetes | Synergistia | Synergistales | Synergistaceae | Candidatus Tammella |  | 194 | 0.183 |
| Bacteria | Proteobacteria | Deltaproteobacteria | Desulfuromonadales |  |  |  | 187 | 0.176 |
| Bacteria | Proteobacteria | Deltaproteobacteria | Syntrophobacterales | Desulfobacteraceae | Desulfosarcina | ovata | 182 | 0.171 |
| Bacteria | Proteobacteria | Deltaproteobacteria | Desulfuromonadales | Geobacteraceae | Geobacter |  | 178 | 0.168 |
| Bacteria | Verrucomicrobia | Methylacidiphilae | Methylacidiphilales | Methylacidiphilaceae | Candidatus Methylacidiphilum |  | 167 | 0.157 |
| Archaea | Euryarchaeota | Methanomicrobia | Methanomicrobiales | Methanomicrobiaceae | Methanofollis | liminatans | 163 | 0.153 |
| Bacteria | Acidobacteria | Acidobacteria | Acidobacteriales | Acidobacteriaceae | Granulicella | tundricola | 148 | 0.139 |
| Bacteria | Actinobacteria | Actinobacteria | Actinomycetales | Actinosynnemataceae |  |  | 146 | 0.137 |
| Bacteria | Synergistetes | Synergistia | Synergistales | Synergistaceae |  |  | 142 | 0.134 |
| Bacteria | Spirochaetes | Spirochaetes | Sphaerochaetales | Sphaerochaetaceae | Sphaerochaeta | globus | 138 | 0.13 |
| Bacteria | Proteobacteria | Deltaproteobacteria | Syntrophobacterales |  |  |  | 138 | 0.13 |
| Bacteria | Bacteroidetes | Flavobacteriia | Flavobacteriales | Flavobacteriaceae | Flavobacterium | terrigena | 136 | 0.128 |
| Bacteria | Firmicutes | Clostridia | Clostridiales | Clostridiaceae | Caloramator | uzoniensis | 136 | 0.128 |
| Bacteria | Bacteroidetes | Sphingobacteriia | Sphingobacteriales | Sphingobacteriaceae |  |  | 134 | 0.126 |
| Bacteria | Actinobacteria | Acidimicrobiia | Acidimicrobiales | Acidimicrobiaceae | Acidimicrobium |  | 128 | 0.121 |
| Bacteria | Proteobacteria | Deltaproteobacteria | Desulfobacterales | Desulfobulbaceae |  |  | 126 | 0.119 |
| Bacteria | Firmicutes | Clostridia | Clostridiales | Clostridiaceae |  |  | 122 | 0.115 |
| Bacteria | Firmicutes | Clostridia | Clostridiales | Veillonellaceae | Anaeromusa |  | 122 | 0.115 |
| Archaea | Euryarchaeota | Halobacteria | Halobacteriales | Halobacteriaceae | Halorubrum | cibi | 120 | 0.113 |
| Bacteria | Firmicutes | Clostridia | Natranaerobiales | Anaerobrancaceae | Anaerobranca | zavarzinii | 117 | 0.11 |
| Bacteria | Spirochaetes | Spirochaetes | Sphaerochaetales | Sphaerochaetaceae | Sphaerochaeta |  | 113 | 0.106 |
| Bacteria | Proteobacteria | Deltaproteobacteria | Myxococcales | Nannocystaceae | Nannocystis |  | 111 | 0.105 |
| Bacteria | Firmicutes | Clostridia | Clostridiales | Eubacteriaceae | Acetobacterium |  | 107 | 0.101 |
| Archaea | Euryarchaeota | Methanomicrobia | Methanomicrobiales | Methanocorpusculaceae | Methanocorpusculum | parvum | 107 | 0.101 |
| Bacteria | Thermotogae | Thermotogae | Thermotogales | Thermotogaceae | Fervidobacterium |  | 107 | 0.101 |
| Bacteria | Proteobacteria | Deltaproteobacteria | Desulfovibrionales | Desulfovibrionaceae | Desulfovibrio | fairfieldensis | 107 | 0.101 |
| Bacteria | Firmicutes | Clostridia | Clostridiales | Clostridiaceae | Sedimentibacter | hydroxybenzoicus | 106 | 0.1 |
| Bacteria | Firmicutes | Erysipelotrichi | Erysipelotrichales | Erysipelotrichaceae | Erysipelothrix | muris | 106 | 0.1 |
| Bacteria | Chloroflexi | Thermobacula | Thermobaculales | Thermobaculaceae | Thermobaculum | terrenum | 99 | 0.093 |
| Bacteria | Bacteroidetes | Flavobacteriia | Flavobacteriales | Flavobacteriaceae | Flavobacterium |  | 98 | 0.092 |
| Bacteria | Firmicutes | Clostridia | Clostridiales | Syntrophomonadaceae | Syntrophomonas | sapovorans | 96 | 0.09 |
| Bacteria | Chloroflexi | Anaerolineae | Anaerolineales | Anaerolinaceae |  |  | 92 | 0.087 |
| Bacteria | Firmicutes | Clostridia | Clostridiales | Lachnospiraceae | Johnsonella | ignava | 92 | 0.087 |
| Bacteria | Actinobacteria | Actinobacteria | Actinomycetales | Propionibacteriaceae | Tessaracoccus |  | 90 | 0.085 |
| Bacteria | Proteobacteria | Deltaproteobacteria |  |  |  |  | 90 | 0.085 |
| Bacteria | Firmicutes | Clostridia | Clostridiales | Clostridiaceae | Clostridium | alkalicellulosi | 86 | 0.081 |
| Archaea | Euryarchaeota | Methanomicrobia | Methanomicrobiales | Methanospirillaceae | Methanospirillum |  | 86 | 0.081 |
| Bacteria | Firmicutes | Clostridia | Clostridiales | Clostridiaceae | Peptoniphilus | coxii | 85 | 0.08 |
| Bacteria | Firmicutes | Clostridia | Clostridiales | Peptococcaceae | Pelotomaculum |  | 85 | 0.08 |
| Bacteria | Proteobacteria | Deltaproteobacteria | Desulfovibrionales | Desulfohalobiaceae | Desulfonauticus | autotrophicus | 82 | 0.077 |
| Bacteria | Bacteroidetes | Flavobacteriia | Flavobacteriales | Flavobacteriaceae | Polaribacter | butkevichii | 81 | 0.076 |
| Bacteria | Bacteroidetes | Bacteroidia | Bacteroidales | Porphyromonadaceae | Dysgonomonas | wimpennyi | 80 | 0.075 |
| Bacteria | Proteobacteria | Deltaproteobacteria | Desulfuromonadales | Desulfuromonadaceae |  |  | 78 | 0.073 |
| Bacteria | Firmicutes | Clostridia | Clostridiales | Veillonellaceae | Phascolarctobacterium |  | 77 | 0.072 |
| Bacteria | Bacteroidetes | Sphingobacteriia | Sphingobacteriales | Chitinophagaceae |  |  | 76 | 0.072 |
| Bacteria | Firmicutes | Bacilli | Bacillales | Bacillaceae | Geobacillus |  | 72 | 0.068 |
| Bacteria | Firmicutes | Clostridia | Coriobacteriales | Coriobacteriaceae | Slackia |  | 70 | 0.066 |
| Bacteria | Firmicutes | Clostridia | Clostridiales | Heliobacteriaceae | Heliorestis | baculata | 69 | 0.065 |
| Bacteria | Synergistetes | Synergistia | Synergistales | Dethiosulfovibrionaceae | Aminobacterium | colombiense | 69 | 0.065 |
| Bacteria | Proteobacteria | Deltaproteobacteria | Syntrophobacterales | Desulfobacteraceae | Desulfobacter |  | 67 | 0.063 |
