# Supplementary material for: Methane-yielding microbial communities processing lactate-rich substrates: a piece of the anaerobic digestion puzzle
Source: Biotechnol Biofuels. 2018 Apr 21;11:116. doi: 10.1186/s13068-018-1106-z (PMC5910564; doi:10.1186/s13068-018-1106-z)
Supplement: Supplementary file 5 — Additional file 5. Lactate utilization genes in identified species and genera with known genome sequences. [file 13068_2018_1106_MOESM5_ESM.docx]

Additional file 5. Lactate utilization genes in identified species and genera with known genome sequences.

|  |  | LldP | DH | EtfA | EtfB | LarA |  | Rnf | Hydrogenase |
| --- | --- | --- | --- | --- | --- | --- | --- | --- | --- |
| Firmicutes, Acidaminococcales | Acidaminococcus fermentans DSM 20731 |  | GlcD |  |  |  |  | Rnf | 2 FeFe_hydrog_A |
|  |  |  | GlcD |  |  |  |  |  |  |
|  |  |  | GlcD |  |  |  |  |  |  |
|  |  |  |  | EtfA | EtfB |  |  |  |  |
|  |  |  |  | EtfA | EtfB |  |  |  |  |
| Synergistetes, Synergistales | Aminiphilus circumscriptus DSM 16581 |  | GlcD | EtfA | EtfB, |  |  |  | FeFe_hydrog_A, Ni,Fe-hydrogenase III large subunit |
|  |  |  | GlcD, |  |  |  | Ldh_2 |  |  |
|  |  |  | GlcD |  |  |  |  |  |  |
|  |  |  | GlcD |  |  |  |  |  |  |
|  |  |  |  |  |  | LarA (3 pr) |  |  |  |
| Synergistetes, Synergistales | Aminobacterium colombiense DSM 12261 | LldP, |  |  |  | LarA |  | Rnf | 2 Ni,Fe-hydrogenase III large subunit |
|  |  |  |  |  |  | LarA | Ldh_2, |  |  |
|  |  |  |  |  |  | LarA |  |  |  |
| Bacteroidetes, Flavobacteriales | Aquimarina macrocephali JAMB N27 |  |  |  |  |  | GlcD+N-GlpC |  |  |
|  |  |  |  | EtfA | EtfB |  |  |  |  |
| Bacteroidetes, Bacteroidales | Bacteroides xylanisolvens CL03T12C04 | LldP |  |  |  |  |  | Rnf | FeFe_hydrog_A |
|  |  |  |  |  |  |  | C-GlpC,LutB,LutC |  |  |
|  |  |  |  | EtfA | EtfB |  |  |  |  |
| Chloroflexi, Anaerolineales | Bellilinea caldifistulae GOMI-1 | LldP (2 pr) |  |  |  |  |  |  | Ni,Fe-hydrogenase III large subunit |
|  |  |  |  | EtfA | EtfB |  |  |  |  |
|  |  |  |  |  |  | LarA |  |  |  |
| Firmicutes, Clostridiales | Caloramator mitchellensis VF08 |  |  | EtfA | EtfB |  |  |  | 3 FeFe_hydrog_A, Ni,Fe-hydrogenase I large subunit |
|  | Candidatus Amoebophilus asiaticus 5a2 |  |  |  |  |  |  |  |  |
| Verrucomicrobia, Chthoniobacterales | Chthoniobacter flavus Ellin428 |  |  |  |  |  | GlcD+N-GlpC (&DUF3390) |  |  |
|  |  |  | GlcD, |  |  |  | GlpC |  |  |
|  |  |  | GlcD, |  |  |  | N-GlcD,GlpC |  |  |
|  |  |  | GlcD (tr) |  |  |  |  |  |  |
|  |  |  | GlcD |  |  |  |  |  |  |
|  |  |  |  |  |  |  | GlpC (tr at both ends),LutB,LutC |  |  |
|  |  | LldP |  |  |  |  |  |  |  |
|  |  |  |  |  |  | LarA |  |  |  |
|  |  |  |  |  |  | LarA |  |  |  |
| Firmicutes, Clostridiales | Clostridium taeniosporum 1/k | LldP, | GlcD | EtfA | EtfB |  |  |  | 4 FeFe_hydrog_A |
|  |  |  |  | EtfA | EtfB |  |  |  |  |
| -Proteobacteria, Desulfobacterales | Desulfobulbus elongatus DSM 2908 | LldP | GlcD |  |  |  | GlpC,LutC,LutB+GlpC |  | 3 Ni,Fe-hydrogenase III large subunit, 2 FeFe_hydrog_A |
|  |  |  | GlcD, |  |  | LarA | GlpC |  |  |
|  |  |  |  |  |  |  | GlcD+N-GlpC |  |  |
|  |  |  |  |  |  |  | GlcD+GlpC |  |  |
|  |  |  |  | EtfA | EtfB |  | empty(nitrate)+GlpC |  |  |
|  |  |  |  | EtfA | EtfB |  |  |  |  |
| -Proteobacteria, Syntrophobacterales | Desulfomonile tiedjei DSM 6799 | LldP, | GlcD |  |  |  | GlpC | Rnf | Ni,Fe-hydrogenase III large subunit, Ni,Fe-hydrogenase I large subunit , FeFe_hydrog_A |
|  |  |  |  |  |  |  | GlcD+GlpC |  |  |
|  |  |  |  |  |  |  | N-GlcD,GlpC |  |  |
|  |  |  |  |  |  |  | C-GlcD,N-GlcD(repeat region/transposase) |  |  |
|  |  |  |  |  |  |  | N-GlcD |  |  |
|  |  |  |  |  |  |  | C-GlpC,LutB |  |  |
|  |  |  |  | EtfA | EtfB |  | empty+GlpC |  |  |
|  |  |  |  |  |  |  | LutC |  |  |
|  |  |  |  | EtfA | EtfB |  |  |  |  |
|  |  |  |  | EtfA | EtfB |  |  |  |  |
|  |  |  |  |  |  | LarA (2 pr) |  |  |  |
| -Proteobacteria, Desulfovibrionales | Desulfovibrio fairfieldensis CCUG 45958 |  | GlcD, |  |  |  | GlpC | Rnf | Ni,Fe-hydrogenase III large subunit, Ni,Fe-hydrogenase I large subunit, FeFe_hydrog_A |
|  |  | LldP | GlcD, |  |  |  | GlpC,LutC,LutB+GlpC |  |  |
|  |  |  |  |  |  |  | GlcD+N-GlpC |  |  |
|  |  | LldP |  |  |  |  |  |  |  |
| Firmicutes, Bacillales incertae sedis | Gemella cuniculi DSM 15828 | LldP (2 pr) |  |  |  |  |  |  |  |
| -Proteobacteria, Desulfuromonadales | Geobacter pickeringii G13 | LldP, | GlcD |  |  |  | GlpC, LutB+GlpC,LutC |  | Ni,Fe-hydrogenase III large subunit, 2 Ni,Fe-hydrogenase I large subunit |
|  |  |  | GlcD |  |  | LarA, | GlpC |  |  |
|  |  |  |  | EtfA | EtfB |  | empty(nitrate)+GlpC |  |  |
|  |  |  |  | EtfA | EtfB |  | empty+GlpC |  |  |
|  |  |  |  | EtfA | EtfB |  | empty+GlpC |  |  |
|  |  |  |  |  |  |  | empty(nitrate)+GlpC |  |  |
|  |  |  |  | EtfA | EtfB |  | empty+GlpC |  |  |
|  |  |  |  | EtfA | EtfB |  |  |  |  |
|  |  |  |  | EtfA | EtfB |  |  |  |  |
| Bacteroidetes, Flavobacteriales | Gramella forsetii KT0803 |  |  |  |  |  | GlcD+N-GlpC |  |  |
|  |  |  |  | EtfA | EtfB |  |  |  |  |
| Firmicutes, Clostridiales | Johnsonella ignava ATCC 51276 | LldP, | GlcD | EtfA | EtfB |  |  | Rnf | FeFe_hydrog_A |
|  |  |  |  | EtfA | EtfB |  |  |  |  |
| Firmicutes, Clostridiales | Lachnospira pectinoschiza M83 |  |  |  |  |  |  |  |  |
| Chloroflexi, Anaerolineales | Longilinea arvoryzae KOME-1 |  |  |  |  |  | GlcD+GlpC |  | 3 Ni,Fe-hydrogenase III large subunit |
|  |  |  | GlcD |  |  |  |  |  |  |
|  |  |  |  |  |  | LarA (3 pr) |  |  |  |
| Euryarchaeota, Methanomicrobiales | Methanocorpusculum labreanum Z |  |  |  |  |  |  |  | Ni,Fe-hydrogenase III large subunit |
| Euryarchaeota, Methanomicrobiales | Methanofollis liminatans DSM 4140 |  |  |  |  |  |  |  | 3 Ni,Fe-hydrogenase III large subunit |
| Euryarchaeota, Methanosarcinales | Methanosaeta concilii GP-6 |  | GlcD,., |  |  |  | GlpC |  |  |
|  |  |  |  |  |  | LarA |  |  |  |
| Firmicutes, Veillonellales | Negativicoccus succinicivorans DORA_17_25 Q612_NSC00001 |  |  |  |  | LarA |  |  |  |
|  |  |  |  | EtfA | EtfB |  |  |  |  |
| Firmicutes, Clostridiales | Peptococcus niger DSM 20475 |  | GlcD, | EtfA | EtfB |  |  |  | FeFe_hydrog_A |
|  |  |  | GlcD |  |  |  |  |  |  |
|  |  |  |  |  |  |  | GlcD,GlpC |  |  |
|  |  |  |  |  |  |  | N-GlpC(tr),C-GlpC(tr),LutB |  |  |
|  |  |  |  |  |  |  | LutB,LutC |  |  |
|  |  |  |  |  |  |  | C-GlpC,LutB,LutC |  |  |
|  |  | LldP |  |  |  |  |  |  |  |
|  |  |  |  | EtfA | EtfB |  |  |  |  |
|  |  |  |  | EtfA | EtfB |  |  |  |  |
| Firmicutes, Tissierellales | Peptoniphilus coxii DNF00729 |  |  | EtfA | EtfB |  | HicDH_like (ldh) | Rnf | FeFe_hydrog_B1 |
|  |  |  |  | EtfA | EtfB |  |  |  |  |
|  |  |  |  | EtfA (tr) |  |  |  |  |  |
| Firmicutes, Acidaminococcales | Phascolarctobacterium succinatutens YIT 12067 |  | GlcD |  |  |  |  |  | 3 FeFe_hydrog_A (1 with Rubrerythrin domain) |
|  |  |  | GlcD |  |  |  |  |  |  |
|  |  |  | GlcD |  |  |  |  |  |  |
|  |  |  | GlcD |  |  |  |  |  |  |
|  |  |  |  | EtfA | EtfB |  |  |  |  |
|  |  |  |  |  |  | LarA | HicDH_like (ldh) |  |  |
|  |  |  |  |  |  | LarA |  |  |  |
|  |  |  |  |  |  |  |  |  |  |
| Bacteroidetes, Cytophagales | Runella limosa DSM 17973 |  | GlcD |  |  |  |  |  | Ni,Fe-hydrogenase I large subunit |
|  |  |  |  |  |  |  | GlcD+N-GlpC |  |  |
|  |  |  |  |  |  |  | GlcD+N-GlpC |  |  |
|  |  |  |  |  |  |  | C-GlpC,LutB,LutC |  |  |
|  |  |  |  | EtfA | EtfB |  |  |  |  |
| Firmicutes, Selenomonadales | Selenomonas infelix ATCC 43532 |  |  |  |  |  |  |  | Ni,Fe-hydrogenase I large subunit |
| Spirochaetes, Spirochaetales | Sphaerochaeta globosa str. Buddy |  | GlcD | EtfA | EtfB, |  |  | Rnf | 2 FeFe_hydrog_A |
| Firmicutes, Clostridiales | Syntrophomonas palmitatica JCM 14374 |  | GlcD, |  |  |  | GlpC | Rnf | 3 FeFe_hydrog_A |
|  |  |  |  | EtfA | EtfB, |  | empty(nitrate)+GlpC |  |  |
|  |  |  |  | EtfA | EtfB |  |  |  |  |
|  |  |  |  | EtfA | EtfB |  |  |  |  |
| Firmicutes, Clostridiales | Syntrophomonas wolfei subsp. wolfei str. Goettingen G311 |  | GlcD, |  |  |  | GlpC |  | 3 FeFe_hydrog_A |
|  |  |  |  | EtfA | EtfB |  | empty(nitrate)+GlpC |  |  |
|  |  |  |  | EtfA | EtfB |  |  |  |  |
|  |  |  |  | EtfA | EtfB |  |  |  |  |
| Firmicutes, Clostridiales | Syntrophomonas wolfei subsp. Methylbutyratica |  | GlcD, |  |  |  | GlpC |  | 3 FeFe_hydrog_A, Ni,Fe-hydrogenase I large subunit |
|  |  |  |  | EtfA | EtfB, |  | empty(nitrate)+GlpC |  |  |
|  |  |  |  | EtfA | EtfB |  |  |  |  |
|  |  |  |  | EtfA | EtfB |  |  |  |  |
| unclassified Terrabacteria group | Thermobaculum terrenum ATCC BAA-798 |  | GlcD |  |  |  |  |  |  |
|  |  |  | GlcD |  |  |  | N-GlcD,GlpC |  |  |
|  |  |  |  |  |  |  | GlcD+N-GlpC |  |  |
|  |  |  | GlcD |  |  |  | C-GlpC |  |  |
|  |  |  |  |  |  |  | LutC,LutB+GlpC |  |  |
|  |  |  |  | EtfA | EtfB |  |  |  |  |
| Nitrospirae, Nitrospirales | Thermodesulfovibrio thiophilus DSM 17215 | LldP, | GlcD |  |  |  | GlpC,LutC,LutB+GlpC |  | 2 Ni,Fe-hydrogenase III large subunit, Ni,Fe-hydrogenase I large subunit |
|  |  |  | GlcD |  |  |  |  |  |  |
|  |  |  |  | EtfA | EtfB |  |  |  |  |
|  |  | LldP |  |  |  |  |  |  |  |
| Spirochaetes, Spirochaetales | Treponema brennaborense DSM 12168 |  |  |  |  |  |  | Rnf | FeFe_hydrog_A |
|  |  |  |  |  |  |  |  |  |  |
|  |  | LldP | DH | EtfA | EtfB | LarA |  | Rnf | Hydrogenase |
| Tenericutes, Acholeplasmatales | Acholeplasma |  | GlcD (5 sp/9 st) | EtfA (5 st, 1 sp/5 st) | EtfB (5 st, 1 sp/5 st) |  |  | Rnf (11) |  |
| Actinobacteria, Acidimicrobiales | Acidimicrobium ferrooxidans DSM 10331 |  | GlcD |  |  |  |  | Rnf |  |
|  |  |  | GlcD, | EtfA | EtfB |  | N-GlcD,GlpC |  |  |
|  |  |  |  |  |  |  | GlpC,GlcD |  |  |
|  |  |  | GlcD |  |  |  |  |  |  |
|  |  |  |  | EtfA | EtfB |  |  |  |  |
|  |  |  |  |  |  | N-LarA |  |  |  |
| Firmicutes, Clostridiales | Alkaliphilus metalliredigens QYMF | LldP | GlcD | EtfA | EtfB | LarA |  | Rnf | 2 FeFe_hydrog_A |
|  |  | LldP | GlcD | EtfA | EtfB |  |  |  |  |
|  |  |  | GlcD |  |  |  |  |  |  |
|  |  |  | GlcD | EtfA | EtfB |  |  |  |  |
|  |  |  |  | EtfA | EtfB |  |  |  |  |
|  |  |  |  |  |  | LarA (3 pr) |  |  |  |
| Firmicutes, Clostridiales | Alkaliphilus transvaalensis ATCC 700919 | LldP | GlcD | EtfA | EtfB | LarA |  | Rnf | 2 FeFe_hydrog_A |
|  |  |  |  |  |  | LarA |  |  |  |
| Firmicutes, Clostridiales | Anaerobranca californiensis DSM 14826 | LldP | GlcD | EtfA | EtfB | LarA |  | Rnf | 2 FeFe_hydrog_A |
|  |  |  |  | EtfA | EtfB |  |  |  |  |
| Firmicutes, Selenomonadales | Anaeromusa acidaminophila DSM 3853 |  | GlcD | EtfA | EtfB, |  | GlpC,GlcD |  | 2 FeFe_hydrog_A, Ni,Fe-hydrogenase I large subunit |
|  |  |  |  |  |  |  | GlcD+(N)-GlpC |  |  |
|  |  |  |  |  |  |  | LutC,LutB+GlpC |  |  |
|  |  | LldP |  |  |  |  |  |  |  |
|  |  | LldP |  |  |  |  |  |  |  |
|  |  | LldP (2 pr) |  |  |  |  |  |  |  |
|  |  |  |  |  |  | LarA (4 pr) |  |  |  |
| Firmicutes, Selenomonadales | Anaerovibrio | LldP | GlcD | EtfA | EtfB |  | LutC,LutB+GlpC |  | FeFe_hydrog_A |
|  |  |  | GlcD, |  |  |  | GlpC |  |  |
|  |  |  |  |  |  | LarA |  |  |  |
| Chloroflexi, Anaerolineales | Bellilinea | LldP (2 pr) |  |  |  |  |  |  | Ni,Fe-hydrogenase III large subunit |
|  |  |  |  | EtfA | EtfB |  |  |  |  |
|  |  |  |  |  |  | LarA |  |  |  |
| Firmicutes, Clostridiales | Blautia hydrogenotrophica DSM 10507 |  | GlcD | N-EtfA(tr),gap,C-EtfA(tr) | EtfB |  |  | Rnf | 3 FeFe_hydrog_A |
|  |  |  | GlcD | EtfA | EtfB |  |  |  |  |
|  |  |  |  | EtfA | EtfB, |  | N-GlcD(tr),gap,C-GlcD(tr) |  |  |
|  |  |  | GlcD |  |  |  |  |  |  |
|  |  |  |  |  |  | LarA (2 pr) |  |  |  |
| Firmicutes, Clostridiales | Blautia hydrogenotrophica 2789STDY5608857 |  | GlcD | EtfA | EtfB |  |  | Rnf | 3 FeFe_hydrog_A |
|  |  |  | GlcD | C-EtfA | EtfB |  |  |  |  |
|  |  |  | GlcD | (C)-EtfA | EtfB |  |  |  |  |
|  |  |  | GlcD | EtfA | EtfB |  |  |  |  |
|  |  |  | GlcD |  |  |  |  |  |  |
|  |  |  |  |  |  | LarA (2 pr) |  |  |  |
| Firmicutes, Clostridiales | Blautia sp. GD8 |  | GlcD | EtfA | EtfB |  |  |  | FeFe_hydrog_A |
| Firmicutes, Clostridiales | Blautia sp. KLE 1732 |  | GlcD | EtfA | EtfB |  |  |  | FeFe_hydrog_A |
| Bacteroidetes, Bacteroidales | Bacteroides | LldP |  |  |  |  |  | Rnf | FeFe_hydrog_A, Ni,Fe-hydrogenase I large subunit (2 st) |
|  |  |  |  |  |  |  | GlcD+GlpC (84 str, 2 for 10 st) |  |  |
|  |  |  |  |  |  |  | GlpC,LutB,LutC |  |  |
|  |  |  |  | EtfA | EtfB |  |  |  |  |
| Firmicutes, Thermoanaerobacterales | Caldicellulosiruptor | LldP |  |  |  |  |  |  | FeFe_hydrog_A |
|  |  |  |  | EtfA | EtfB |  |  |  |  |
| Chloroflexi, Caldilineales | Caldilinea aerophila DSM 14535 |  |  |  |  |  | GlcD+N-GlcD |  | 2 Ni,Fe-hydrogenase III large subunit |
|  |  |  | GlcD |  |  |  | N-GlcD,GlpC |  |  |
|  |  |  | GlcD |  |  |  |  |  |  |
|  |  |  |  |  |  |  | GlcD+N-GlpC |  |  |
|  |  |  |  | EtfA | EtfB |  |  |  |  |
| Firmicutes, Clostridiales | Caloramator |  |  | EtfA | EtfB |  |  |  | 2 FeFe_hydrog_A (3 for C.m. VF08), Ni,Fe-hydrogenase I large subunit |
| Bacteroidetes, Cytophagales | Candidatus Amoebophilus asiaticus |  |  |  |  |  |  |  |  |
| Bacteroidetes, Flavobacteriales | Chryseobacterium |  |  |  |  |  | N-GlcD |  |  |
|  |  |  |  | EtfA | EtfB |  |  |  |  |
| Synergistetes, Synergistales | Cloacibacillus |  | GlcD | EtfA | EtfB, |  |  | Rnf | 2/3 FeFe_hydrog_A |
|  |  |  | GlcD | EtfA | EtfB, |  |  |  |  |
|  |  |  | GlcD |  |  |  |  |  |  |
|  |  |  | GlcD |  |  | LarA (for C.e. DSM 19522) |  |  |  |
|  |  |  |  | EtfA (for C.e. DSM 19522) | EtfB (for C.e. DSM 19522) |  |  |  |  |
|  |  | LldP (for C.e. DSM 19522) |  |  |  |  |  |  |  |
|  |  |  |  |  |  | LarA |  |  |  |
| -Proteobacteria, Desulfobacterales | Desulfobacter curvatus DSM 3379 |  | GlcD, |  |  |  | GlpC | Rnf | 3 Ni,Fe-hydrogenase I large subunit |
|  |  | LldP, | GlcD |  |  |  | GlpC |  |  |
|  |  |  |  |  |  |  | GlcD+GlpC |  |  |
|  |  |  | GlcD (N-term+C-term) |  |  |  |  |  |  |
|  |  |  | GlcD |  |  | LarA |  |  |  |
|  |  |  |  | EtfA | EtfB |  | empty+GlpC |  |  |
|  |  |  |  | EtfA | EtfB |  |  |  |  |
|  | Desulfobacter postgatei 2ac9 |  | GlcD |  |  |  |  | Rnf | 2 Ni,Fe-hydrogenase I large subunit |
|  |  |  | GlcD |  |  | LarA |  |  |  |
|  |  |  |  |  |  |  | GlcD+GlpC |  |  |
|  |  |  |  | EtfA | EtfB |  | empty+GlpC |  |  |
|  |  | LldP |  |  |  |  |  |  |  |
|  | Desulfobacter vibrioformis DSM 8776 |  | GlcD, |  |  |  | GlpC | Rnf | 2 Ni,Fe-hydrogenase I large subunit |
|  |  |  |  |  |  |  | GlcD+GlpC |  |  |
|  |  |  | GlcD, |  |  |  | GlpC |  |  |
|  |  |  | GlcD |  |  | LarA |  |  |  |
|  |  |  |  |  |  |  | C-GlpC,LutB,LutC |  |  |
|  |  |  |  | EtfA | EtfB |  | empty+GlpC |  |  |
|  |  |  |  | EtfA | EtfB |  |  |  |  |
| -Proteobacteria, Desulfovibrionales | Desulfonauticus sp. 38_4375 PW |  | GlcD |  |  |  |  |  | Ni,Fe-hydrogenase III large subunit, Ni,Fe-hydrogenase I large subunit |
|  |  |  |  |  |  |  | GlcD+GlpC |  |  |
|  |  |  |  |  |  |  | GlcD,N-GlpC(tr,scaffold) |  |  |
|  |  |  |  | EtfA | EtfB |  |  |  |  |
| -Proteobacteria, Desulfobacterales | Desulfosarcina cetonica JCM 12296 |  |  |  |  |  | N-GlcD,C-GlpC | Rnf | Ni,Fe-hydrogenase III large subunit, 3 Ni,Fe-hydrogenase I large subunit |
|  |  |  |  |  |  |  | N-GlcD(tr),C-GlcD(tr),N-GlpC(tr),C-GlpC1(tr),C-GlpC2(tr) |  |  |
|  |  |  |  |  |  |  | GlcD+N-GlpC (tr,contig) |  |  |
|  |  |  | GlcD | EtfA | EtfB |  | GlpC(&CIMS_C_terminal_like),empty(nitrate)+GlpC |  |  |
|  |  |  | PLN02806 |  |  |  | N-GlpC&C-GlpC(overlaid) |  |  |
|  |  |  |  |  |  |  | C-GlcD(at the edge of contig),GlpC,LutC,LutB,C-GlpC |  |  |
|  |  | LldP, |  |  |  |  | N-GlcD(tr,contig) |  |  |
|  |  |  |  |  |  |  | N-GlcD(tr),C-GlcD(tr),GlpC |  |  |
|  |  |  |  | EtfA | EtfB |  |  |  |  |
|  |  |  |  | EtfA | EtfB |  | empty+GlpC, |  |  |
|  |  |  |  | EtfA | EtfB |  |  |  |  |
|  |  |  |  | EtfA | EtfB |  |  |  |  |
|  |  |  |  | EtfA | EtfB |  |  |  |  |
|  |  |  |  |  |  |  | LutC |  |  |
|  |  |  |  |  |  |  | N-LutB,C-LutB |  |  |
| -Proteobacteria, Desulfovibrionales | Desulfovibrio |  |  |  |  |  |  | up to 3 | up to 2 FeFe_hydrog_A, up to 3 Ni,Fe-hydrogenase I large subunit, Ni,Fe-hydrogenase III large subunit |
|  | Desulfovibrio aespoeensis Aspo-2, Desulfovibrio gigas DSM 1382, Desulfovibrio inopinatus DSM 10711 | LldP | GlcD |  |  |  | GlpC,LutC,LutB+GlpC |  | (+) Ni,Fe-hydrogenase III large subunit (only D.i. DSM 10711) |
|  |  |  |  |  |  |  | GlcD+GlpC |  |  |
|  |  |  |  |  |  |  | GlcD,GlpC |  |  |
|  | Desulfovibrio hydrothermalis AM13 | LldP | GlcD |  |  |  | GlpC,LutC,LutB+GlpC |  |  |
|  |  |  |  |  |  |  | GlcD+GlpC |  |  |
|  |  |  |  |  |  |  | GlcD,N-GlpC |  |  |
|  | Desulfovibrio africanus, Desulfovibrio magneticus RS-1 (only 1 LlpD) | LldP | GlcD |  |  |  | GlpC,LutC,LutB+GlpC |  | (+) 2 Ni,Fe-hydrogenase III large subunit (only 1st st, D.a. PCS only 1 pr) |
|  |  | LldP |  |  |  |  |  |  |  |
|  |  |  |  |  |  |  | GlcD+GlpC |  |  |
|  |  |  |  |  |  |  | GlcD,GlpC |  |  |
|  |  |  | GlcD |  |  |  |  |  |  |
|  | Desulfovibrio alaskensis | LldP | GlcD |  |  |  | GlpC,LutC,LutB+GlpC |  |  |
|  |  |  |  |  |  |  | GlcD+N-GlpC |  |  |
|  |  |  |  |  |  |  | GlcD+N-GlpC |  |  |
|  |  |  | GlcD |  |  |  | ,.,N-GlpC |  |  |
|  |  |  | GlcD |  |  |  |  |  |  |
|  | Desulfovibrio alcoholivorans DSM 5433, Desulfovibrio bastinii DSM 16055, Desulfovibrio fructosivorans JJ (+GlcD), Desulfovibrio longus DSM 6739, Desulfovibrio magneticus RS-1 | LldP | GlcD |  |  |  | GlpC,LutC,LutB+GlpC |  |  |
|  |  |  |  |  |  |  | GlcD+(N)-GlpC |  |  |
|  |  |  |  |  |  |  | GlcD+N-GlpC |  |  |
|  |  |  |  |  |  |  | GlcD,GlpC |  |  |
|  | Desulfovibrio alkalitolerans DSM 16529, Desulfovibrio frigidus DSM 17176 | LldP | GlcD |  |  |  | GlpC,LutC,LutB+GlpC |  |  |
|  |  |  |  |  |  |  | GlcD+N-GlpC |  |  |
|  |  |  |  |  |  |  | GlcD,GlpC |  |  |
|  | Desulfovibrio fairfieldensis CCUG 45958 | LldP | GlcD |  |  |  | GlpC,LutC,LutB+GlpC |  |  |
|  |  |  |  |  |  |  | GlcD+(N)-GlpC |  |  |
|  |  |  |  |  |  |  | GlcD,GlpC |  |  |
|  |  | LldP |  |  |  |  |  |  |  |
|  | Desulfovibrio aminophilus DSM 12254, Desulfovibrio oxyclinae DSM 11498 | LldP | GlcD |  |  |  | GlpC,LutC,LutB+GlpC |  | (+) Ni,Fe-hydrogenase III large subunit |
|  |  |  |  |  |  |  | GlcD+GlpC |  |  |
|  |  |  | GlcD |  |  |  |  |  |  |
|  |  |  |  |  |  | LarA (only 1 st) |  |  |  |
|  | Desulfovibrio brasiliensis JCM 12178 | N-LldP,C-LldP(tr) |  |  |  |  | N-GlcD,C-GlcD(tr,contig) |  | (+) Ni,Fe-hydrogenase III large subunit |
|  |  |  |  |  |  |  | GlcD+GlpC |  |  |
|  |  |  |  |  |  |  | C-GlcD(tr,contig),GlpC,LutC,LutB,C-GlpC |  |  |
|  |  |  | GlcD |  |  |  |  |  |  |
|  | Desulfovibrio cuneatus DSM 11391 | LldP |  |  |  |  |  |  | (+) Ni,Fe-hydrogenase III large subunit |
|  |  | LldP |  |  |  |  |  |  |  |
|  |  |  | GlcD |  |  |  | GlpC,LutC,LutB+GlpC |  |  |
|  |  |  |  |  |  |  | GlcD+(N)-GlpC |  |  |
|  |  |  |  |  |  |  | GlcD+N-GlpC |  |  |
|  |  |  |  |  |  |  | GlcD,GlpC |  |  |
|  |  |  |  |  |  | LarA |  |  |  |
|  | Desulfovibrio desulfuricans ND132 | LldP | GlcD |  |  |  | GlpC,LutC,LutB+GlpC |  |  |
|  |  |  |  |  |  |  | GlcD,GlpC |  |  |
|  |  |  |  |  |  |  | GlcD+N-GlpC |  |  |
|  |  |  |  |  |  |  | GlcD+N-GlpC |  |  |
|  |  |  |  |  |  |  | GlcD+GlpC |  |  |
|  | Desulfovibrio desulfuricans DSM 7057 (+LldP), Desulfovibrio desulfuricans subsp. desulfuricans str. ATCC 27774 | LldP |  |  |  |  |  |  |  |
|  |  | LldP |  |  |  |  |  |  |  |
|  |  |  | GlcD |  |  |  | GlpC,LutC,LutB+GlpC |  |  |
|  |  |  |  |  |  |  | GlcD,GlpC |  |  |
|  |  |  |  |  |  |  | GlcD+N-GlpC |  |  |
|  |  |  |  |  |  |  | GlcD+GlpC |  |  |
|  |  |  |  |  |  | LarA |  |  |  |
|  | Desulfovibrio desulfuricans DSM 17919 | LldP | GlcD |  |  |  | GlpC,LutC,LutB+GlpC |  |  |
|  |  |  |  |  |  |  | GlcD,(N)-GlpC |  |  |
|  |  |  |  |  |  |  | GlcD+(N)-GlpC |  |  |
|  |  |  |  |  |  |  | GlcD+N-GlpC |  |  |
|  |  |  |  |  |  |  | GlcD+N-GlpC |  |  |
|  |  | LldP |  |  |  |  |  |  |  |
|  | Desulfovibrio desulfuricans subsp. desulfuricans DSM 642 | LldP | GlcD |  |  |  | GlpC,LutC,LutB+GlpC |  |  |
|  |  |  |  |  |  |  | GlcD,GlpC |  |  |
|  |  |  |  |  |  |  | GlcD,GlpC |  |  |
|  |  |  |  |  |  |  | GlcD+(N)-GlpC |  |  |
|  |  |  |  |  |  |  | GlcD+N-GlpC |  |  |
|  |  | LldP |  |  |  |  |  |  |  |
|  |  |  |  |  |  | LarA |  |  |  |
|  | Desulfovibrio litoralis DSM 11393 | LldP |  |  |  |  |  |  |  |
|  |  | LldP |  |  |  |  |  |  |  |
|  |  |  | GlcD |  |  |  | GlpC |  |  |
|  |  |  |  |  |  |  | GlcD+GlpC |  |  |
|  |  |  |  |  |  |  | LutC,LutB+GlpC |  |  |
|  |  |  |  |  |  | LarA |  |  |  |
|  | Desulfovibrio piezophilus C1TLV30 | LldP | GlcD |  |  |  | GlpC,LutC,LutB+GlpC |  |  |
|  |  |  |  |  |  |  | GlcD+GlpC |  |  |
|  |  |  |  |  |  |  | GlcD+GlpC |  |  |
|  |  |  |  |  |  |  | GlcD,(N)-GlpC |  |  |
|  | Desulfovibrio piger ATCC 29098 (+LlpD), Desulfovibrio piger isolate DESPIGER1 | LldP | GlcD |  |  |  | GlpC,LutC,LutB+GlpC |  |  |
|  |  |  |  |  |  |  | GlcD+(N)-GlpC |  |  |
|  |  | LldP |  |  |  |  |  |  |  |
|  |  |  |  |  |  | LarA |  |  |  |
|  | Desulfovibrio putealis DSM 16056 | LldP | GlcD |  |  |  | GlpC,LutC,LutB+GlpC |  | (+) Ni,Fe-hydrogenase III large subunit |
|  |  |  |  |  |  |  | GlcD+(N)-GlpC |  |  |
|  |  |  |  |  |  |  | GlcD+N-GlpC |  |  |
|  |  |  |  |  |  |  | GlcD,(N)-GlpC |  |  |
|  |  | LldP |  |  |  |  | LutC,LutB+GlpC |  |  |
|  | Desulfovibrio salexigens DSM 2638, Desulfovibrio sp. L21-Syr-AB | LldP | GlcD |  |  |  | GlpC,LutC,LutB+GlpC |  | (+) Ni,Fe-hydrogenase III large subunit (only Desulfovibrio sp. L21-Syr-AB) |
|  |  |  |  |  |  |  | GlcD+(N)-GlpC |  |  |
|  |  |  |  |  |  |  | GlcD,(N)-GlpC |  |  |
|  | Desulfovibrio sp. 3_1_syn3, Desulfovibrio sp. 6_1_46AFAA | LldP | GlcD |  |  |  | GlpC,LutC,LutB+GlpC |  |  |
|  |  |  |  |  |  |  | GlcD+(N)-GlpC |  |  |
|  |  |  |  |  |  |  | GlcD,GlpC |  |  |
|  |  | LldP |  |  |  |  |  |  |  |
|  |  |  |  |  |  | LarA |  |  |  |
|  | Desulfovibrio sp. A2, Desulfovibrio termitidis HI1, Desulfovibrio vulgaris str. 'Miyazaki F' (-LldP) | LldP | GlcD |  |  |  | GlpC,LutC,LutB+GlpC |  |  |
|  |  |  |  |  |  |  | GlcD+GlpC |  |  |
|  |  |  |  |  |  |  | GlcD+N-GlpC |  |  |
|  |  |  |  |  |  |  | GlcD,(N)-GlpC |  |  |
|  |  |  | GlcD |  |  |  |  |  |  |
|  |  | LldP (4 pr) |  |  |  |  |  |  |  |
|  |  |  |  |  |  | LarA |  |  |  |
|  | Desulfovibrio sp. J2 | LldP | GlcD |  |  |  | GlpC,LutC,LutB+GlpC |  |  |
|  |  |  |  |  |  |  | GlcD+N-GlpC |  |  |
|  |  |  |  |  |  |  | GlcD+GlpC |  |  |
|  |  |  | GlcD |  |  |  |  |  |  |
|  | Desulfovibrio sp. TomC | LldP | GlcD |  |  |  | GlpC,LutC,LutB+GlpC |  |  |
|  |  |  |  |  |  |  | GlcD+N-GlpC |  |  |
|  |  |  |  |  |  |  | GlcD+(N)-GlpC |  |  |
|  |  |  |  |  |  |  | GlcD,GlpC |  |  |
|  |  |  |  |  |  |  | GlcD,GlpC |  |  |
|  |  |  | GlcD |  |  |  |  |  |  |
|  |  |  |  |  |  | LarA |  |  |  |
|  | Desulfovibrio sp. DV, Desulfovibrio sp. FW1012W, Desulfovibrio sp. U5L | LldP | GlcD |  |  |  | GlpC,LutC,LutB+GlpC |  |  |
|  |  |  |  |  |  |  | GlcD+N-GlpC |  |  |
|  |  |  |  |  |  |  | GlcD+(N)-GlpC |  |  |
|  |  |  |  |  |  |  | GlcD,GlpC |  |  |
|  |  |  | GlcD |  |  |  |  |  |  |
|  |  |  |  |  |  |  | N-GlcD |  |  |
|  | Desulfovibrio vulgaris DP4, Desulfovibrio vulgaris RCH1, Desulfovibrio vulgaris str. Hildenborough | LldP | GlcD |  |  |  | GlpC,LutC,LutB+GlpC |  |  |
|  |  |  |  |  |  |  | GlcD+(N)-GlpC |  |  |
|  |  |  |  |  |  |  | GlcD+(N)-GlpC |  |  |
|  |  |  |  |  |  |  | GlcD,(N)-GlpC |  |  |
|  |  |  | GlcD |  |  |  |  |  |  |
|  |  | LldP (5 pr) |  |  |  |  |  |  |  |
|  | Desulfovibrio sp. X2 | LldP | GlcD |  |  |  | GlpC,LutC,LutB+GlpC |  | (+) Ni,Fe-hydrogenase III large subunit |
|  |  | LldP |  |  |  |  |  |  |  |
|  |  |  |  |  |  |  | GlcD+(N)-GlpC |  |  |
|  |  |  |  |  |  |  | GlcD,(N)-GlpC |  |  |
|  |  | LldP |  |  |  |  |  |  |  |
|  | Desulfovibrio zosterae DSM 11974 | LldP | GlcD |  |  |  | GlpC,LutC,LutB+GlpC |  |  |
|  |  |  |  |  |  |  | GlcD,N-GlpC |  |  |
|  |  |  |  |  |  |  | GlcD+GlpC |  |  |
|  |  |  |  |  |  |  | GlcD+(N)-GlpC |  |  |
|  |  |  |  |  |  |  | GlcD+(N)-GlpC |  |  |
| Synergistetes, Synergistales | Dethiosulfovibrio peptidovorans DSM 11002 |  |  |  |  | LarA | Ldh_2, |  | 2 FeFe_hydrog_B1, Ni,Fe-hydrogenase III small subunit |
| Cyanobacteria, Nostocales | Dolichospermum |  | GlcD |  |  |  |  |  | Ni,Fe-hydrogenase III large subunit, 3 Ni,Fe-hydrogenase I large subunit |
|  |  |  |  |  |  |  | N-GlcD,GlpC |  |  |
| Bacteroidetes, Bacteroidales | Dysgonomonas |  |  |  |  |  | GlcD+N-GlpC | RnfC,RnfD |  |
|  |  | LldP, |  |  |  |  | C-GlpC,LutB,LutC (for D.c . DSM 22835, D.m. DSM 27370, D.m. JCM 19375, D. HGC4) |  |  |
|  |  |  | ALDH_LactADH_AldA (for D.c . DSM 22835, D.m. DSM 27370, D.m. JCM 19375, D. HGC4) |  |  |  |  |  |  |
|  | Dysgonomonas gadei ATCC BAA-286 |  |  |  |  |  | GlcD+N-GlpC |  |  |
|  |  |  | ALDH_LactADH_AldA |  |  |  | C-GlpC,LutB,LutC |  |  |
| Bacteroidetes, Flavobacteriales | Flavobacterium |  |  | EtfA | EtfB |  |  |  | Ni,Fe-hydrogenase I large subunit (7 st) |
|  | Flavobacterium akiainvivens |  | GlcD |  |  |  |  | RnfC,RnfD (only 1st st) |  |
|  | Flavobacterium antarcticum DSM 19726 |  |  |  |  |  |  |  |  |
|  | Flavobacterium aquatile LMG 4008 |  |  |  |  |  |  |  |  |
|  | Flavobacterium beibuense F44-8 |  |  |  |  |  |  |  |  |
|  | Flavobacterium cucumis DSM 18830 |  |  |  |  |  |  |  |  |
|  | Flavobacterium filum DSM 17961 |  |  |  |  |  |  |  |  |
|  | Flavobacterium fontis DSM 25660 |  |  |  |  |  |  |  |  |
|  | Flavobacterium gelidilacus DSM 15343 |  |  |  |  |  |  |  |  |
|  | Flavobacterium haoranii DSM 22807 |  |  |  |  |  |  |  |  |
|  | Flavobacterium indicum GPTSA100-9 |  |  |  |  |  |  |  |  |
|  | Flavobacterium limnosediminis JC2902 |  |  |  |  |  |  |  |  |
|  | Flavobacterium psychrophilum |  |  |  |  |  |  |  |  |
|  | Flavobacterium rivuli |  |  |  |  |  | C-GlpC,LutB,LutC |  |  |
|  | Flavobacterium saliperosum |  |  |  |  |  |  |  |  |
|  | Flavobacterium sasangense DSM 21067 |  |  |  |  |  |  |  |  |
|  | Flavobacterium soli DSM 19725 |  |  |  |  |  |  |  |  |
|  | Flavobacterium sp. 316 |  |  |  |  |  |  |  |  |
|  | Flavobacterium sp. Leaf359 |  |  |  |  |  |  |  |  |
|  | Flavobacterium suncheonense DSM 17707 |  |  |  |  |  |  |  |  |
|  | Flavobacterium tegetincola DSM 22377 |  |  |  |  |  |  |  |  |
|  | Flavobacterium terrae DSM 18829 |  |  |  |  |  |  |  |  |
|  |  |  |  |  |  |  |  |  |  |
|  | Flavobacterium aquidurense DSM 18293 |  |  |  |  |  | GlcD+N-GlpC |  |  |
|  | Flavobacterium denitrificans DSM 15936 |  |  |  |  |  | GlcD+N-GlpC |  |  |
|  | Flavobacterium frigidimaris DSM 15937 |  |  |  |  |  | C-GlpC,.,LutB,LutC (only 1st,2nd st) |  |  |
|  |  |  |  |  |  |  | C-GlpC,LutB,LutC |  |  |
|  | Flavobacterium frigoris DSM 15719 |  |  |  |  |  |  |  |  |
|  | Flavobacterium fryxellicola DSM 16209 |  |  |  |  |  |  |  |  |
|  | Flavobacterium johnsoniae DSM 2064 |  |  |  |  |  | C-GlpC,LutB,LutC |  | Ni,Fe-hydrogenase I large subunit |
|  | Flavobacterium johnsoniae DSM 6792 |  |  |  |  |  | C-GlpC,LutB,LutC |  |  |
|  | Flavobacterium johnsoniae UW101 |  |  |  |  |  | C-GlpC,LutB,LutC |  | Ni,Fe-hydrogenase I large subunit |
|  | Flavobacterium saccharophilum DSM 1811 |  |  |  |  |  | 2 C-GlpC,LutB,LutC |  |  |
|  | Flavobacterium segetis DSM 19741 |  |  |  |  |  |  |  |  |
|  | Flavobacterium sp. ABG |  |  |  |  |  |  |  |  |
|  | Flavobacterium sp. ACAM 123 ACAM123 |  |  |  |  |  |  |  |  |
|  | Flavobacterium sp. Fl |  |  |  |  |  | C-GlpC,LutB,LutC |  |  |
|  | Flavobacterium sp. Root420 |  |  |  |  |  | C-GlpC,LutB,LutC |  |  |
|  | Flavobacterium subsaxonicum WB 4.1-42 = DSM 21790 |  |  |  |  |  | C-GlpC,LutB,LutC |  |  |
|  | Flavobacterium xinjiangense CGMCC 1.2749 |  |  |  |  |  |  |  |  |
|  |  |  |  |  |  |  |  |  |  |
|  | Flavobacterium aquidurense RC62 |  |  |  |  |  | GlcD+N-GlpC |  |  |
|  | Flavobacterium branchiophilum FL-15 |  |  |  |  |  | C-GlpC,.,LutB,LutC (only 1st,3rd,19th,21th st) |  |  |
|  | Flavobacterium chungangense LMG 26729 |  |  |  |  |  |  |  |  |
|  | Flavobacterium daejeonense |  |  |  |  |  | C-GlpC,LutB,LutC |  |  |
|  | Flavobacterium defluvii DSM 17963 |  |  |  |  |  |  |  |  |
|  | Flavobacterium flevense DSM 1076 |  |  |  |  |  | C-GlpC,LutB,LutC |  |  |
|  | Flavobacterium frigidarium DSM 17623 |  |  |  |  |  |  |  |  |
|  | Flavobacterium frigoris PS1 66_18 |  |  |  |  |  |  |  |  |
|  | Flavobacterium glycines |  |  |  |  |  | C-GlpC,LutB,LutC |  |  |
|  | Flavobacterium granuli DSM 19729 |  |  |  |  |  | C-GlpC,LutB,LutC |  |  |
|  | Flavobacterium hydatis DSM 2063 |  |  |  |  |  |  |  | Ni,Fe-hydrogenase I large subunit |
|  | Flavobacterium pectinovorum DSM 6368 |  |  |  |  |  | C-GlpC,LutB,LutC |  |  |
|  | Flavobacterium piscis CCUG 60099 FLP35 |  |  |  |  |  | C-GlpC,LutB,LutC |  | Ni,Fe-hydrogenase I large subunit |
|  | Flavobacterium reichenbachii LMG 25512 |  |  |  |  |  | C-GlpC,LutB,LutC |  |  |
|  | Flavobacterium seoulense EM1321 |  |  |  |  |  | C-GlpC,LutB,LutC |  |  |
|  | Flavobacterium sp. 83 |  |  |  |  |  | C-GlpC,LutB,LutC |  |  |
|  | Flavobacterium sp. AED |  |  |  |  |  | C-GlpC,LutB,LutC |  |  |
|  | Flavobacterium sp. CF108 |  |  |  |  |  | C-GlpC,LutB,LutC |  |  |
|  | Flavobacterium sp. CF136 |  |  |  |  |  |  |  |  |
|  | Flavobacterium sp. JRM |  |  |  |  |  |  |  | Ni,Fe-hydrogenase I large subunit |
|  | Flavobacterium sp. KJJ |  |  |  |  |  |  |  |  |
|  | Flavobacterium sp. KMS |  |  |  |  |  |  |  | Ni,Fe-hydrogenase I large subunit |
|  | Flavobacterium sp. Leaf82 |  |  |  |  |  | C-GlpC,LutB,LutC |  |  |
|  | Flavobacterium sp. LPB0076 |  |  |  |  |  |  |  |  |
|  | Flavobacterium sp. MEB061 |  |  |  |  |  | C-GlpC,LutB,LutC |  |  |
|  | Flavobacterium sp. PK15 |  |  |  |  |  | C-GlpC,LutB,LutC |  |  |
|  | Flavobacterium sp. Root186 |  |  |  |  |  | C-GlpC,LutB,LutC |  |  |
|  | Flavobacterium sp. Root901 |  |  |  |  |  | C-GlpC,LutB,LutC |  |  |
|  | Flavobacterium sp. Root935 |  |  |  |  |  | C-GlpC,LutB,LutC |  |  |
|  | Flavobacterium sp. TAB 87 |  |  |  |  |  | C-GlpC,LutB,LutC |  |  |
|  | Flavobacterium sp. VMW |  |  |  |  |  | C-GlpC,LutB,LutC |  |  |
|  | Flavobacterium xanthum DSM 3661 |  |  |  |  |  |  |  |  |
|  |  |  |  |  |  |  |  |  |  |
|  | Flavobacterium cauense R2A-7 |  | GlcD |  |  |  |  |  |  |
|  | Flavobacterium enshiense DK69 |  | PLN02806 |  |  |  |  |  |  |
|  |  |  |  |  |  |  |  |  |  |
|  | Flavobacterium chilense |  |  |  |  |  | GlcD+N-GlpC |  |  |
|  | Flavobacterium hibernum DSM 12611 |  |  |  |  |  | GlcD+N-GlpC |  |  |
|  | Flavobacterium johnsoniae CI04 |  |  |  |  |  | GlcD+N-GlpC |  | Ni,Fe-hydrogenase I large subunit |
|  | Flavobacterium johnsoniae GSE09 |  |  |  |  |  | C-GlpC,.,LutB,LutC (only 1st st) |  |  |
|  | Flavobacterium sp. F52 |  |  |  |  |  | C-GlpC,LutB,LutC (2 for 2nd st, no for 1st st) |  |  |
|  |  |  |  |  |  |  |  |  |  |
|  | Flavobacterium columnare |  | PLN02806 |  |  |  |  |  |  |
|  |  |  |  |  |  |  | GlcD+N-GlpC |  |  |
|  |  |  |  |  |  |  |  |  |  |
|  | Flavobacterium fluvii DSM 19978 | LldP |  |  |  |  | GlcD+N-GlpC |  |  |
|  |  |  |  |  |  |  | C-GlpC,.,LutB,LutC |  |  |
|  | Flavobacterium gilvum EM1308 | LldP |  |  |  |  | GlcD+N-GlpC(tr),C-GlpC |  |  |
|  |  |  |  |  |  |  | GlcD+N-GlpC |  |  |
|  |  |  |  |  |  |  | C-GlpC,.,LutB,LutC |  |  |
|  | Flavobacterium micromati DSM 17659 | LldP |  |  |  |  |  |  |  |
|  |  |  |  |  |  |  | GlcD+N-GlpC |  |  |
|  | Flavobacterium sp. URHB0058 | LldP |  |  |  |  | C-GlpC,LutB,LutC,GlcD+N-GlpC |  |  |
|  |  |  |  |  |  |  | C-GlpC,.,LutB,LutC |  |  |
|  |  |  |  |  |  |  | GlcD+N-GlpC |  |  |
|  |  |  |  |  |  |  | GlcD+N-GlpC |  |  |
|  | Flavobacterium sp. WG21 |  | PLN02806 |  |  |  | GlcD+N-GlpC |  |  |
|  | Flavobacterium spartansii MSU |  |  |  |  |  | GlcD+N-GlpC |  |  |
|  |  |  |  |  |  |  |  |  |  |
|  | Flavobacterium succinicans LMG |  |  |  |  |  | GlcD+N-GlpC(tr),C-GlpC |  |  |
|  | Flavobacterium succinicans DSM 4002 |  |  |  |  |  |  |  |  |
|  |  |  |  |  |  |  |  |  |  |
|  | Flavobacterium succinicans DD5b |  |  |  |  |  | GlcD+N-GlpC(tr),C-GlpC |  |  |
|  |  |  |  |  |  |  | GlcD+N-GlpC |  |  |
| -Proteobacteria, Desulfuromonadales | Geobacter anodireducens SD-1 |  | GlcD |  |  | LarA, | GlpC |  | 2 Ni,Fe-hydrogenase III large subunit (1 tr), 3 Ni,Fe-hydrogenase I large subunit (1 tr) |
|  | Geobacter metallireducens GS-15 |  | GlcD |  |  | LarA, | GlpC |  | 2 Ni,Fe-hydrogenase III large subunit, 1 Ni,Fe-hydrogenase I large subunit |
|  |  |  |  |  |  |  | (N)-GlcD (4-cresol dehydrogenase) |  |  |
|  |  |  |  |  |  |  | GlcD (4-cresol dehydrogenase) |  |  |
|  |  |  |  | EtfA | EtfB |  | empty+GlpC,empty+GlpC |  |  |
|  |  |  |  | EtfA | EtfB |  | empty+GlpC, |  |  |
|  |  |  |  | EtfA | EtfB |  | empty+GlpC, |  |  |
|  |  |  |  | EtfA | EtfB |  | empty+GlpC, |  |  |
|  |  |  |  | EtfA | EtfB |  | empty+GlpC, |  |  |
|  |  |  |  | EtfA | EtfB |  | empty(TM_PBP1)+GlpC, |  |  |
|  | Geobacter metallireducens RCH3 |  | GlcD |  |  | LarA, | GlpC |  | 2 Ni,Fe-hydrogenase III large subunit, 1 Ni,Fe-hydrogenase I large subunit |
|  |  |  |  |  |  |  | (N)-GlcD (4-cresol dehydrogenase) |  |  |
|  |  |  |  |  |  |  | GlcD (4-cresol dehydrogenase) |  |  |
|  |  |  |  | EtfA | EtfB |  | empty+GlpC,empty+GlpC |  |  |
|  |  |  |  | EtfA | EtfB |  | empty(nitrate)+GlpC |  |  |
|  |  |  |  | EtfA | EtfB |  | empty+GlpC, |  |  |
|  |  |  |  | EtfA | EtfB |  | empty(ABC-2)+GlpC, |  |  |
|  | Geobacter soli GSS01 |  | GlcD |  |  | LarA, | GlpC |  | 2 Ni,Fe-hydrogenase III large subunit, 3 Ni,Fe-hydrogenase I large subunit |
|  |  |  |  | EtfA | EtfB |  | empty(nitrate)+GlpC, |  |  |
|  |  |  |  | EtfA | EtfB |  | empty+GlpC, |  |  |
|  | Geobacter sp. OR-1 |  | GlcD |  |  | LarA, | GlpC |  | 3 Ni,Fe-hydrogenase III large subunit, 3 Ni,Fe-hydrogenase I large subunit |
|  |  |  |  | EtfA | EtfB |  | empty+GlpC, |  |  |
|  | Geobacter bemidjiensis Bem |  | GlcD |  |  | LarA, | GlpC |  | 2 Ni,Fe-hydrogenase III large subunit, 3 Ni,Fe-hydrogenase I large subunit |
|  |  | LldP | GlcD |  |  |  | GlpC, LutC, LutB+GlpC |  |  |
|  |  |  |  |  |  |  | GlcD+N-GlpC |  |  |
|  |  |  |  | EtfA | EtfB |  | empty+GlpC, |  |  |
|  |  |  |  | EtfA | EtfB |  | empty+GlpC, |  |  |
|  |  |  |  | EtfA | EtfB |  | empty+GlpC, |  |  |
|  |  |  |  | EtfA | EtfB |  | empty+GlpC |  |  |
|  |  |  |  | EtfA | EtfB |  | empty+GlpC |  |  |
|  |  |  |  | EtfA | EtfB |  | empty+GlpC |  |  |
|  | Geobacter bremensis R1 |  | GlcD |  |  | LarA, | GlpC |  | Ni,Fe-hydrogenase III large subunit, 2 Ni,Fe-hydrogenase I large subunit |
|  |  |  |  |  |  |  | GlcD+N-GlpC |  |  |
|  |  | LldP | GlcD |  |  |  | GlpC, LutC, LutB+GlpC |  |  |
|  |  |  |  |  |  |  | (N)-GlcD (4-cresol dehydrogenase) |  |  |
|  |  |  |  |  |  |  | N-GlcD (4-cresol dehydrogenase) |  |  |
|  |  |  |  | EtfA | EtfB |  | empty+GlpC, |  |  |
|  |  |  |  | EtfA | EtfB |  | empty+GlpC, |  |  |
|  |  |  |  | EtfA | EtfB |  | empty+GlpC |  |  |
|  |  |  |  | EtfA | EtfB |  | empty+GlpC |  |  |
|  |  |  |  | EtfA | EtfB |  | empty+GlpC |  |  |
|  |  |  |  | EtfA | EtfB |  | empty+GlpC |  |  |
|  |  |  |  | EtfA | EtfB |  | empty(nitrate)+GlpC |  |  |
|  | Geobacter daltonii FRC-32 |  | GlcD |  |  | LarA, | GlpC |  | Ni,Fe-hydrogenase III large subunit |
|  |  |  |  |  |  |  | GlcD+N-GlpC |  |  |
|  |  |  |  |  |  |  | N-GlcD(C-terminal domain truncated) |  |  |
|  |  |  |  |  |  |  | (N)-GlcD (4-cresol dehydrogenase) |  |  |
|  |  |  |  |  |  |  | (N)-GlcD (4-cresol dehydrogenase) |  |  |
|  |  |  |  | EtfA | EtfB |  | empty+GlpC |  |  |
|  |  |  |  | EtfA | EtfB |  | empty+GlpC,empty+GlpC |  |  |
|  |  |  |  | EtfA | EtfB |  | empty+GlpC |  |  |
|  | Geobacter sp. M18**,** Geobacter sp. M21, Geobacter sulfurreducens (3 st) | LldP | GlcD |  |  |  | GlpC, LutC, LutB+GlpC |  | Ni,Fe-hydrogenase III large subunit (+1 for G. M21 and G.s.), 3 Ni,Fe-hydrogenase I large subunit (-1 for G. M21 and G.s.) |
|  |  |  | GlcD |  |  | LarA, | GlpC |  |  |
|  |  |  |  |  |  |  | (N)-GlcD (4-cresol dehydrogenase, for M. M21) |  |  |
|  |  |  |  |  |  |  | N-GlcD (4-cresol dehydrogenase, for M. M21) |  |  |
|  | only 1 for G.s. |  |  | EtfA | EtfB |  | empty+GlpC |  |  |
|  |  |  |  | EtfA | EtfB |  | empty(nitrate)+GlpC |  |  |
|  |  |  |  | EtfA | EtfB |  | empty+GlpC |  |  |
|  |  |  |  | EtfA | EtfB |  | empty+GlpC |  |  |
|  |  |  |  | EtfA | EtfB |  | empty+GlpC |  |  |
|  |  |  |  | EtfA | EtfB |  | empty+GlpC |  |  |
|  | Geobacter lovleyi SZ |  |  | EtfA | EtfB |  | empty+GlpC |  | 2 Ni,Fe-hydrogenase III large subunit, 2 Ni,Fe-hydrogenase I large subunit |
|  | Geobacter uraniireducens Rf4 | LldP, | GlcD |  |  |  | GlpC |  | Ni,Fe-hydrogenase III large subunit, 3 Ni,Fe-hydrogenase I large subunit |
|  |  |  | GlcD |  |  | LarA, | GlpC |  |  |
|  |  | LldP, |  |  |  |  | LutC, LutB+GlpC |  |  |
|  |  |  |  | EtfA | EtfB |  | empty(nitrate)+GlpC |  |  |
|  |  |  |  | EtfA | EtfB |  | empty+GlpC |  |  |
|  |  |  |  | EtfA | EtfB |  | empty+GlpC |  |  |
| Cyanobacteria, Synechococcales | Leptolyngbya |  | GlcD |  |  |  |  |  | Ni,Fe-hydrogenase III large subunit (for L. Heron Island J, L. KIOST-1 LS, L. boryana PCC 6306, L. PCC 7375) |
|  |  |  |  |  |  |  | N-GlcD, GlpC |  |  |
|  |  |  |  |  |  |  | N-GlcD |  |  |
|  |  | LldP (for L. Heron Island J, L. KIOST-1 LS, L. O-77, L. PCC 6406) |  |  |  |  |  |  |  |
| Firmicutes, Veillonellales | Megasphaera elsdenii 14-14 |  | GlcD |  |  |  |  | Rnf | FeFe_hydrog_A |
|  |  |  | GlcD |  |  |  |  |  |  |
|  |  |  | GlcD |  |  | LarA, |  |  |  |
|  |  | LldP, | GlcD |  |  | LarA |  |  |  |
|  |  |  | GlcD |  |  |  |  |  |  |
|  |  |  | GlcD |  |  |  |  |  |  |
|  |  |  |  |  |  |  | LutC,LutB+GlpC |  |  |
|  |  | LldP |  |  |  |  |  |  |  |
|  |  |  |  |  |  | LarA |  |  |  |
|  |  |  |  | EtfA | EtfB |  |  |  |  |
|  |  |  |  | EtfA | EtfB |  |  |  |  |
| Euryarchaeota, Methanobacteriales | Methanobacterium |  |  |  |  |  | C-GlpC(tr),LutB |  | 3 Ni,Fe-hydrogenase III large subunit |
| Euryarchaeota, Methanomicrobiales | Methanocorpusculum |  |  |  |  |  |  |  | Ni,Fe-hydrogenase III large subunit (2 st) |
| Euryarchaeota, Methanomicrobiales | Methanoculleus |  |  |  |  |  |  |  | 2 Ni,Fe-hydrogenase III large subunit |
| Euryarchaeota, Methanosarcinales | Methanosaeta |  | GlcD, |  |  |  | GlpC |  |  |
|  |  |  |  |  |  | LarA |  |  |  |
| Bacteroidetes, Flavobacteriales | Myroides |  |  |  |  |  | GlcD+N-GlpC |  |  |
|  |  | LldP (18 st) |  |  |  |  | C-GlpC,LutB,LutC (15 st) |  |  |
|  |  |  |  | EtfA | EtfB (18 st) |  |  |  |  |
| Bacteroidetes, Bacteroidales | Parabacteroides |  |  |  |  |  | GlcD+N-GlpC (20 st) | Rnf | FeFe_hydrog_A (33 st), FeFe_hydrog_B1 (33 st), Ni,Fe-hydrogenase I large subunit (30 st) |
|  |  |  |  |  |  |  | C-GlpC,LutB,LutC (34 st) |  |  |
|  |  |  |  | EtfA | EtfB |  |  |  |  |
| Bacteroidetes, Sphingobacteriales | Pedobacter antarcticus ATCC 51969 |  | GlcD |  |  |  |  |  |  |
|  |  |  |  |  |  |  | C-GlpC,LutB,LutC |  |  |
|  |  |  |  |  |  |  | LutB |  |  |
|  |  |  |  | EtfA | EtfB |  |  |  |  |
|  | Pedobacter antarcticus DSM 11725 |  | GlcD |  |  |  |  |  |  |
|  |  |  |  |  |  |  | C-GlpC,LutB,LutC |  |  |
|  |  |  |  |  |  |  | LutB |  |  |
|  |  |  |  | EtfA | EtfB |  |  |  |  |
|  | Pedobacter arcticus A12 |  |  |  |  |  | GlcD+N-GlpC |  |  |
|  |  |  |  |  |  |  | GlcD+N-GlpC |  |  |
|  |  |  |  | EtfA | EtfB |  |  |  |  |
|  |  |  |  |  |  |  | LutB |  |  |
|  |  |  |  |  |  |  | C-GlpC,LutB,LutC |  |  |
|  |  |  |  |  |  |  | LutB |  |  |
|  | Pedobacter caeni DSM 16990 |  | GlcD |  |  |  |  |  |  |
|  |  |  |  |  |  |  | C-GlpC,LutB,LutC |  |  |
|  |  |  |  |  |  |  | LutB |  |  |
|  |  |  |  | EtfA | EtfB |  |  |  |  |
|  | Pedobacter cryoconitis PAMC 27485 |  | GlcD |  |  |  |  |  |  |
|  |  |  | PLN02806 |  |  |  |  |  |  |
|  |  |  |  |  |  |  | GlcD+N-GlpC |  |  |
|  |  |  |  |  |  |  | LutB |  |  |
|  |  |  |  |  |  |  | C-GlpC,LutB,LutC |  |  |
|  |  |  |  |  |  |  |  |  |  |
|  |  |  |  | EtfA | EtfB |  |  |  |  |
|  | Pedobacter glucosidilyticus DSM 23534 |  |  |  |  |  | GlcD+N-GlpC |  |  |
|  |  |  |  |  |  |  | C-GlpC,LutB,LutC |  |  |
|  |  |  |  | EtfA | EtfB |  |  |  |  |
|  | Pedobacter heparinus DSM 2366 |  |  |  |  |  | GlcD+N-GlpC |  |  |
|  |  |  |  |  |  |  | GlcD+N-GlpC |  |  |
|  |  |  |  |  |  |  | LutB |  |  |
|  |  |  |  |  |  |  | C-GlpC,LutB,LutC |  |  |
|  |  |  |  | EtfA | EtfB |  |  |  |  |
|  | Pedobacter oryzae DSM 19973 |  | PLN02806 |  |  |  |  |  |  |
|  | Pedobacter sp. Leaf176 |  |  |  |  |  | LutB |  |  |
|  | Pedobacter sp. PACM 27299 |  |  |  |  |  | LutC (only 1st st) |  |  |
|  | Pedobacter sp. V48 |  |  |  |  |  | C-GlpC,LutB,LutC (no first 2 st) |  |  |
|  | Pedobacter steynii DX4 |  |  | EtfA | EtfB |  |  |  |  |
|  |  |  |  |  |  |  |  |  |  |
|  | Pedobacter steynii DSM 19110 |  | PLN02808 |  |  |  |  |  |  |
|  |  |  | PLN02809 |  |  |  |  |  |  |
|  |  |  |  |  |  |  | GlcD+N-GlpC |  |  |
|  |  |  |  |  |  |  | LutB |  |  |
|  |  |  |  |  |  |  | C-GlpC,LutB,LutC |  |  |
|  |  |  |  | EtfA | EtfB |  |  |  |  |
| Bacteroidetes, Flavobacteriales | Polaribacter |  |  |  |  |  | GlcD+N-GlpC | Rnf (1 st) |  |
|  |  |  |  |  |  |  | C-GlpC,LutB,LutC (2 st) |  |  |
|  |  |  |  | EtfA | EtfB |  |  |  |  |
| Bacteroidetes, Bacteroidales | Porphyromonas |  |  |  |  |  | C-GlpC,LutB,LutC |  |  |
|  |  | LldP |  |  |  |  |  |  |  |
|  |  |  |  | EtfA | EtfB |  |  |  |  |
|  |  |  |  | EtfA | EtfB (49 st) |  |  |  |  |
| Firmicutes, Selenomonadales | Propionispora sp. 2/2-37 |  |  |  |  | LarA |  |  | 2 FeFe_hydrog_A |
|  |  |  |  | EtfA | EtfB |  |  |  |  |
| Synergistetes, Synergistales | Pyramidobacter piscolens W5455 | LldP |  |  |  |  |  | Rnf | FeFe_hydrog_A |
|  |  |  |  |  |  | LarA |  |  |  |
|  |  |  |  | EtfA | EtfB |  |  |  |  |
| Firmicutes, unclassified Tissierellia | Sedimentibacter sp. B4 |  | GlcD | EtfA | EtfB, |  |  | Rnf | 2 FeFe_hydrog_A, Ni,Fe-hydrogenase I large subunit |
|  |  |  | GlcD | EtfA | EtfB, |  |  |  |  |
|  |  |  | GlcD | EtfA | EtfB, |  |  |  |  |
|  |  |  | GlcD, | EtfA | EtfB |  |  |  |  |
|  |  |  |  |  |  | LarA (3 pr) |  |  |  |
|  |  |  |  | EtfA | EtfB |  |  |  |  |
|  |  |  |  | EtfA | EtfB |  |  |  |  |
| Bacteroidetes, Sphingobacteriales | Sphingobacterium |  |  |  |  |  | GlcD+N-GlpC (2 pr for 6 st, 1 for 8) |  |  |
|  |  |  |  |  |  |  | LutB (13 st) |  |  |
|  |  |  |  |  |  |  | C-GlpC,LutB,LutC (4 st) |  |  |
|  |  |  |  |  |  |  | LutC (4 st) |  |  |
|  |  |  |  | EtfA | EtfB |  |  |  |  |
| Firmicutes, Clostridiales | Sulfobacillus thermosulfidooxidans ST | LldP | GlcD, |  |  |  | GlcD,GlpC |  | 2 Ni,Fe-hydrogenase I large subunit |
|  |  |  |  |  |  |  | GlcD+N-GlcD,GlpC |  |  |
|  |  |  |  |  |  |  | GlcD+N-GlcD |  |  |
|  |  |  |  |  |  |  | N-GlcD,GlcD |  |  |
|  |  |  |  |  |  |  | C-GlpC,LutB,LutC |  |  |
|  |  |  |  | EtfA | EtfB |  |  |  |  |
|  |  |  |  | EtfA | EtfB |  |  |  |  |
| Synergistetes, Synergistales | Synergistes |  | GlcD | EtfA | EtfB | LarA, |  | Rnf | 2 FeFe_hydrog_A |
|  |  |  | GlcD | EtfA | EtfB, |  |  |  |  |
|  |  |  | GlcD |  |  |  |  |  |  |
|  |  |  | GlcD |  |  |  |  |  |  |
|  |  |  |  |  |  | LarA (2 pr) |  |  |  |
| Firmicutes, Clostridiales | Syntrophomonas zehnderi OL-4 |  |  |  |  |  | N-GlcD (tr) |  | 3 FeFe_hydrog_A, Ni,Fe-hydrogenase I large subunit |
|  |  |  | GlcD, |  |  |  | GlpC |  |  |
|  |  |  |  |  |  |  | N-GlcD+GlpC |  |  |
|  |  |  |  | EtfA | EtfB, |  | empty(nitrate)+GlpC |  |  |
|  |  |  |  |  |  |  | C-GlcD+GlpC (tr,scaffold,repeat region) |  |  |
|  |  |  |  | EtfA | EtfB |  |  |  |  |
|  |  |  |  | EtfA | EtfB |  |  |  |  |
| -Proteobacteria, Syntrophobacterales | Syntrophus aciditrophicus SB |  | GlcD |  |  |  |  | Rnf | 3 FeFe_hydrog_A, Ni,Fe-hydrogenase III large subunit |
|  |  |  |  |  |  |  | C-GlpC,GlcD,GlpC |  |  |
|  |  |  |  | EtfA | EtfB, |  | empty(nitrate)+GlpC |  |  |
| Euryarchaeota, Thermococcales | Thermococcus |  |  |  |  |  |  |  | 2 to 4 Ni,Fe-hydrogenase III large subunit |
|  | Thermococcus gammatolerans EJ3 |  | GlcD, |  |  |  | GlpC |  | 1 Ni,Fe-hydrogenase III large subunit |
|  | Thermococcus guaymasensis DSM 11113 |  |  |  |  |  |  |  | 3 Ni,Fe-hydrogenase III large subunit |
|  | Thermococcus sibiricus MM 739 |  |  |  |  |  |  |  | 2 Ni,Fe-hydrogenase III large subunit |
|  | Thermococcus sp. 4557 |  |  |  |  |  |  |  | 3 Ni,Fe-hydrogenase III large subunit |
|  | Thermococcus sp. EP1 |  |  |  |  |  |  |  | 2 Ni,Fe-hydrogenase III large subunit |
| Nitrospirae, Nitrospirales | Thermodesulfovibrio |  | GlcD |  |  |  |  |  | 2 Ni,Fe-hydrogenase III large subunit, 1 Ni,Fe-hydrogenase I large subunit |
|  |  | LldP, | GlcD |  |  |  | GlpC,LutC,LutB+GlpC |  |  |
|  |  | LldP |  |  |  |  |  |  |  |
|  |  |  |  | EtfA | EtfB |  |  |  |  |
| Firmicutes, Thermoanaerobacterales | Thermovenabulum gondwanense R270 |  |  |  |  | LarA |  |  | 2 FeFe_hydrog_A |
|  |  |  |  | EtfA | EtfB |  |  |  |  |

The search for genes involved in lactate utilization under anoxic conditions in the sequenced genomes of microbial species related to those found in the bioreactor communities – detailed analysis.

The lactate utilization genes of *Bacteroides xylanisolvens* CL03T12C04 have been described previously. We also identified *B. denticanum* within the bioreactor communities. Analysis of *Bacteroides* species shows that all of them code for LldP and possess an operon composed of GlpC, LutB and LutC genes. Most of these species (84) also have the GlcD/GlpC fusion gene. Additionally, they have genes encoding EtfA/B, Rnf complex and Fe-dependent hydrogenase Fe,Fe_hydrog_A.

For 99 strains of *Chryseobacterium*, only genes encoding GlcD with an N-terminal FAD-binding domain and the EtfA/B complex are present, making these bacteria less likely to utilize lactate as a carbon and energy source.

We identified 6 *Desulfovibrio* species, but only one has a complete/scaffold genome available. Consequently, we analyzed 52 other sequenced species of this genus. The pattern of lactate utilization genes is similar to *D. fairfieldensis* CCUG 45958. For 46 species, an operon containing LldP, GlcD, GlpC, LutC and LutB/GlpC genes is present, with an additional copy of LldP in several of them. The other 5 species contain the aforementioned genes, but not in one operon. In addition, 46 species encode at least one GlcD/GlpC fusion protein and 40 of them possess one operon composed of the GlcD and GlpC genes. Interestingly, *D. putealis* DSM 16056 has a second copy of the operon comprised of LldP, LutC and LutB/GlpC genes. Although *Desulfovibrio* species lack genes encoding EtfA/B, they can express at least one Rnf complex and three hydrogenases: FeFe_hydrog_A, Ni,Fe-hydrogenase III large subunit and Ni,Fe-hydrogenase I large subunit. Also, many of them possess a gene encoding a probable lactate racemase LarA.

We detected one species of *Flavobacterium terrigena* but with no genome sequence data. The 130 strains of *Flavobacterium* analyzed show a large variety of lactate utilization genes. A group of 23 species only possess a GlcD gene, and two of these have an adjacent gene encoding FAD-dependent dehydrogenase. Sixty-three species code for one to three copies of the GlcD/GlpC fusion, with 40 of these having a C-GlpC, LutB and LutC operon. It should be noted that four species, *F. fluvii* DSM 19978, *F. gilvum* EM1308, *F. micromati* DSM 17659 and *Flavobacterium* sp. URHB0058, have a LldP gene, which in three of these occurs in a probable operon with a GlcD/GlpC fusion or C-GlpC, LutB, LutC and GlcD/GlpC genes. In addition, *F*. *saccharophilum* DSM 1811, *F. hibernum* DSM 12611 and *Flavobacterium* sp. URHB0058 have an additional copy of a C-GlpC, LutB and LutC operon. EtfA/B complex genes are found in the genome of every *Flavobacterium* analyzed, but only seven of them encode a Ni,Fe-hydrogenase I large subunit.

Although no genome sequence was available for *Acholeplasma cavigenitalium*, genomic data for 14 different species of this genus show only the presence of GlcD and EtfA/B genes in about half of them, spread throughout the genome, plus 11 strains also contain Rnf complex genes, making them less likely to utilize lactate.

The genome sequence of *Acidimicrobium ferrooxidans* DSM 10331 codes for three GlcD dehydrogenases, with one of these genes in a probable operon with EtfA/B, GlcD and GlpC. A second GlpC, GlcD and EtfA/B operon was also identified. Although this strain encodes a LarA protein with N-terminal domain identified plus an Rnf complex, it lacks LldP genes.

*Alkaliphilus crotonatoxidans* and *A. peptidifermentans* were found in the bioreactor communities. Genomic data for two different *Alkaliphilus* species show the presence of an operon similar to that found in *Clostridium taeniosporum* 1/k, containing a LarA gene, with at least one additional copy in another part of the genome. In addition, *A. metalliredigens* QYMF possesses two additional operons similar to *C. taeniosporum* 1/k, but one lacks the LldP gene, and it also has additional copies of genes encoding GlcD and the EtfA/B complex. Both genomes have Rnf complex and two FeFe_hydrog_A genes.

The genome of a species of the genus *Anaerobranca*, different from the one that we identified, also codes for an operon similar to that of *C. taeniosporum* 1/k, containing a LarA gene, and it possesses one additional copy of the EtfA/B and Rnf complex genes and two FeFe_hydrog_A genes.

We were able to analyze only one genome of the genus *Anaeromusa*. This shows the presence of an operon composed of two LldP, GlcD, EtfA/B and GlpC genes plus an additional GlcD gene copy, and in different parts of the genome, genes encoding four LarA proteins, a GlcD/GlpC fusion, LutC and LutB/GlpC in an operon, two FeFe_hydrog_A copies, and a Ni,Fe-hydrogenase I large subunit.

The three available genomes of the genus *Anaerovibrio* contain a probable operon composed of LldP, GlcD, EtfA/B, LutC and LutB/GlpC genes. There is also an additional small operon encoding GlcD and GlpC, plus separate LarA and FeFe_hydrog_A hydrogenase genes.

We carefully analyzed the genomes of four of 34 species of the *Blautia* genus: *B. hydrogenotrophica* DSM 10507, *B. hydrogenotrophica* 2789STDY5608857, *Blautia* sp. GD8 and *Blautia* sp. KLE 1732. These contain up to two gene clusters composed of two probable GlcD and EtfA/B operons. In addition, *B. hydrogenotrophica* strains possess two LarA and Rnf complex genes, and although all four strains have up to three Fe,Fe_hydrog_A gene copies, they do not encode lactate permease LldP.

In contrast to the *Blautia* genus, 12 strains of the genus *Caldicellulosiruptor* possess LldP, EtfA/B complex and Fe,Fe_hydrog_A genes, but we failed to find genes encoding lactate dehydrogenase.

Although we were unable to investigate the *Caldilinea tarbellica* genome, its relative *C. aerophila* DSM 14535, possesses one operon encoding two GlcD proteins, one with only the FAD-binding domain, plus a GlpC gene. It also encodes GlcD/GlpC and GlcD/GlcD fusions, an additional GlcD, EtfA/B, and two Ni,Fe-hydrogenase III large subunit proteins.

The analysis of two *Cloacibacillus* genomes shows the presence of two operons encoding GlcD and EtfA/B, and two additional copies of the GlcD gene, one of which is located in the vicinity of a LarA gene in the genome of *C. evryensis* DSM 19522 (which contrasts with its location in the *C. porcorum* CL-84 genome). Both strains encode an Rnf complex and at least two FeFe_hydrog_A copies, but a LldP permease gene is only present in *C. evryensis* DSM 19522.

Three strains of *Desulfobacter* are similar to other sulphur-reducing bacteria with regard to the lactate utilization genes they possess. Two have 2 small operons containing the GlcD and GlpC genes, which in *D. curvatus* DSM 3379 are preceded by a LldP gene. All three have a gene encoding a GlcD/GlpC fusion, an additional copy of the GlcD gene in close proximity to LarA, and at least one EtfA/B gene. *D. vibrioformis* DSM 8776 also has a C-GlpC, LutB and LutC operon, but lacks the LldP gene. As in other sulphur-reducing bacteria, these three species carry genes encoding Rnf and at least two Ni,Fe-hydrogenase I large subunits.

Although the genomes of *Desulfonauticus autotrophicus* and *D. submarinus* are not yet available, we found that *Desulfonauticus* sp. 38_4375 PW possesses a gene encoding a GlcD/GlpC fusion, a small operon comprised of GlcD and N-GlpC genes (the latter located at the edge of a scaffold) and an EtfA/B gene. It also has Ni,Fe-hydrogenase I and III large subunit genes, but it lacks genes encoding LldP or LarA.

The close relative of *Desulfosarcina ovata*, *D. cetonica* JCM 12296, was examined for the presence of lactate utilization genes, but this analysis was complicated by the considerable number of contigs. However, it was possible to identify at least three probable operons comprised of genes encoding GlcD and GlpC, one GlcD/GlpC fusion gene, and an operon containing the genes C-GlcD (truncated at the N-terminus), GlpC, LutC, LutB and C-GlpC. Also, the LldP gene was found with a N-GlcD (truncated at the C-terminus) gene, plus six genes coding for EtfA/B, one of which is in an operon with a GlcD gene. Its genome also encodes Rnf, a Ni,Fe-hydrogenase III large subunit, and three Ni,Fe-hydrogenase I large subunits.

*Dethiosulfovibrio peptidovorans* DSM 11002 is unlikely to utilize lactate since its genome only carries the genes for LarA and the previously described Ldh_2, probably in one operon. Similarly, the presence of genes encoding two FeFe_hydrog_B1 proteins and Ni,Fe-hydrogenase III small subunits does not seem to be connected with lactate utilization.

Two strains of *Dolichospermum circinale*, related to *D. curvum*, a strain detected in the bioreactor communities, possess an operon comprised of GlcD and GlpC genes, one additional copy of the GlcD gene, and genes encoding a Ni,Fe-hydrogenase III large subunit and three Ni,Fe-hydrogenase I large subunits.

All eight species of *Dysgonomonas* that we analyzed carry a GlcD/GlpC gene fusion. In addition, four of them possess an operon comprised of the genes for LldP, C-GlpC, LutB and LutC, and the probable NAD-dependent lactaldehyde dehydrogenase ALDH_LactADH_AldA. *D. gadei* ATCC BAA-286 has the *luc* operon containing this ALDH_LactADH_AldA. All of the *Dysgonomonas* species lack genes encoding the complete Rnf complex and any hydrogenases required for lactate utilization.

Although we analyzed the genome sequence of *Geobacter pickeringii* G13, the presence of two other members of this genus in the bioreactor communities prompted us to search for lactate utilization genes in another 15 *Geobacter* strains. All but one show the presence of an operon composed of LarA, GlcD and GlpC genes. Seven strains have an additional operon with genes encoding LldP, GlcD, GlpC and LutC, with LutB/GlpC genes in the opposite direction. Three of these strains possess a GlcD/GlpC gene fusion. The *G. uranireducens* Rf4 genome carries both of the aforementioned operons, except that the GlpC gene is absent from the second one. Also, in all but one strain, between one to seven clusters of genes coding for EtfA/B and GlpC with unidentified N-terminal domains are present, but they are probably not involved in lactate utilization. Although the *Geobacter* species lack Rnf complex genes, they possess one to three genes encoding Ni,Fe-hydrogenase I and III large subunits, with the exception of one species that has only one Ni,Fe-hydrogenase III large subunit gene.

Among 11 analyzed strains of *Leptolyngbya*, all have the small operon comprised of GlcD and GlpC genes, and additional but dispersed copies of the same genes. However, only four of these strains possess genes encoding LldP and Ni,Fe-hydrogenase III large subunit.

Although we could not investigate the genome of *Megasphaera hominis*, analysis of its relative *M. elsdenii* 14-14 shows strong evidence of lactate utilization. It carries two operons composed of GlcD and LarA genes, one also with a LldP gene, plus an operon containing genes for LutC and LutB/GlpC, and an additional four genes for GlcD, one for LldP and one for LarA lactate racemase. Also genes encoding the Rnf complex, FeFe_hydrog_A and two EtfA/B copies are present.

Analysis of 12 *Methanobacterium* genomes only shows the presence of an operon containing C-GlpC and LutB genes plus those encoding three Ni,Fe-hydrogenase III large subunits, which makes it unlikely that they can utilize lactate.

Searches of the genomes of 21 strains of *Myroides* reveal the presence of a GlcD/GlpC gene fusion. In addition, almost all strains possess an operon composed of LldP, C-GlpC, LutB and LutC genes, and a gene encoding EtfA/B.

Among 35 strains of the *Parabacteroides* genus, the genomes of 34 contain an operon comprised of the genes C-GlpC, LutB and LutC, 20 have a gene encoding a GlcD/GlpC fusion, and all have copies of the Rnf complex and EtfA/B genes. These strains also possess genes coding for different hydrogenases, such as FeFe_hydrog_A, FeFe_hydrog_B1 and Ni,Fe-hydrogenase I large subunit.

Eleven out of 13 strains of *Pedobacter* genus carry the *lut* operon containing genes arranged in the order C-GlpC, LutB and LutC, and ten of these have an additional LutB gene elsewhere in the genome. Also, almost all of them possess a GlcD gene, with five strains having one to two GlcD/GlpC gene fusions, so they can express FAD-dependent dehydrogenase. Although *Pedobacter* strains show the presence of EtfA/B genes, we failed to find any encoding the Rnf complex or hydrogenases required for lactate utilization.

In the genomes of 10 *Polaribacter* strains, we identified the GlcD/GlpC gene fusion as well as EtfA/B complex genes. Two of these strains carry an operon comprised of C-GlpC, LutB and LutC genes. On the other hand, these 10 strains lack genes encoding lactate permease LldP, lactate racemase LarA, any of the aforementioned hydrogenases, and they only have genes coding for a single Rnf complex.

The genomes of all 69 *Porphyromonas* strains analyzed carry the *lut* operon composed of genes encoding C-GlpC, LutB and LutC, the lactate permease LldP and the EtfA/B complex. In 49 species of this genus there is a separate copy of the EtfA/B complex genes. However, there are no genes for the Rnf complex or any hydrogenases required for lactate utilization.

We were not able to investigate the genome of *Propionispora hippei*, but that of its relative, *Propionispora* sp. 2/2-37 only contains genes encoding the EtfA/B complex, LarA protein and two FeFe_hydrog_A hydrogenases. This lack of the necessary genes probably means that *P. hippei* can be excluded from the group of lactate-utilizing bacteria; however, it is possible that strains within the same genus may differ in their complement of genes.

The only *Pyramidobacter* species with a sequenced genome, *P. piscolens* W5455, is similar to *Propionispora* sp. 2/2-37 except that it also possesses genes encoding lactate permease LldP and the Rnf complex. It is still unlikely that it can metabolize lactate.

*Sedimentibacter* sp. B4, a relative of two species identified in the bioreactor communities, *S. hydroxybenzoicus* and *S. saalensis*, has four operons composed of the genes GlcD and EtfA/B. In addition, it carries genes encoding three LarA proteins, two more EtfA/B complexes, two FeFe_hydrog_A hydrogenases, an Rnf complex and one Ni,Fe-hydrogenase I large subunit.

Being unable to analyze the genome of *Sphingobacterium shayense*, we examined 14 different species of this genus. These possess genes encoding one to two GlcD/GlpC fusions and an EtfA/B complex, and all but one has a LutB gene. Additionally, four of them possess an operon containing C-GlpC, LutB and LutC genes plus a separate gene for the LutC protein. On the other hand, we did not find genes encoding lactate permease LldP, LarA nor the Rnf complex.

Like other sulphur-oxidizing bacteria, genomes of the six analyzed *Sulfobacillus* strains are well equipped with lactate utilization machinery. For example, *S. thermosulfidooxidans* ST contains an operon composed of LldP, two GlcD and GlpC genes, as well as the *lut* operon comprised of genes encoding C-GlpC, LutB and LutC. In addition, two GlcD/GlcD gene fusions are present, one of which is followed by a GlpC gene. There are also genes coding for two EtfA/B complexes and two Ni,Fe-hydrogenase I large subunits.

*Synergistes* strains are thought likely to utilize lactate as an energy source since the genomes of 6 species of this genus possess two operons comprised of the GlcD and EtfA/B genes, one of which is accompanied by a LarA gene. Moreover, they possess genes encoding two additional copies of GlcD and two more LarA proteins, an Rnf complex and two FeFe_hydrog_A hydrogenases.

Although we were able to investigate the genomes of *Syntrophomonas* *wolfei* subsp. *wolfei* Goettingen G311 and *S. palmitatica* JCM 14374, the bioreactor microbial communities included at least three more species of this genus: *S. cellicola, S. curvata* and *S. sapovorans*. Analysis of the genome of *S. wolfei* subsp. *methylbutyratica* shows that it is identical to *S. wolfei* subsp. *wolfei* Goettingen G311 with regard to its lactate utilization capability, with one copy of the gene encoding Ni,Fe-hydrogenase I large subunit. The *S. zehnderi* OL-4 genome contains two GlcD/GlpC gene fusions, with one truncated, and one Ni,Fe-hydrogenase I large subunit gene.

The *Syntrophus aciditrophicus* SB genome contains a GlcD gene, an operon comprised of genes encoding C-GlpC, GlcD and GlpC, and an EtfA/B gene. In addition, it possesses genes for an Rnf complex, three FeFe_hydrog_A and one Ni,Fe-hydrogenase III large subunit hydrogenases.

Of the 24 strains of *Thermococcus* analyzed, the genomes of only 5 carry an operon comprised of the GlcD and GlpC genes. Although all strains have genes encoding two to four Ni,Fe-hydrogenase III large subunits, it is unlikely that representatives of this genus can utilize lactate as an energy source.

The genome of *Thermovenabulum gondwanense* R270, a relative of a strain detected in the bioreactor communities, contains only genes encoding LarA protein, the EtfA/B complex and two FeFe_hydrog_A hydrogenases. It is unlikely that members of this genus can utilize lactate.
