# Supplementary material for: Methane-yielding microbial communities processing lactate-rich substrates: a piece of the anaerobic digestion puzzle
Source: Biotechnol Biofuels. 2018 Apr 21;11:116. doi: 10.1186/s13068-018-1106-z (PMC5910564; doi:10.1186/s13068-018-1106-z)
Supplement: Supplementary file 6 — Additional file 6. Genes encoding all nine proteins of the reductive acetyl-CoA (Wood–Ljungdahl) pathway (KEGG Module: M00377) found in both microbial communities, M1A and M1B. [file 13068_2018_1106_MOESM6_ESM.docx]

Additional file 6. Genes encoding all nine proteins of the reductive acetyl-CoA (Wood-Ljungdahl) pathway (KEGG Module: M00377) found in both microbial communities, M1A and M1B.

| **EC number** | **KEGG annotation** |
| --- | --- |
| ec:1.2.1.43 | formate dehydrogenase (NADP+); NADP+-dependent formate dehydrogenase; formate dehydrogenase (NADP+) |
| ec:1.2.7.4 | anaerobic carbon-monoxide dehydrogenase; Ni-CODH; carbon-monoxide dehydrogenase (ferredoxin) |
| ec:1.5.1.5 | methylenetetrahydrofolate dehydrogenase (NADP+); N5,N10-methylenetetrahydrofolate dehydrogenase; 5,10-methylenetetrahydrofolate:NADP oxidoreductase; 5,10-methylenetetrahydrofolate dehydrogenase; methylenetetrahydrofolate dehydrogenase; methylenetetrahydrofolate dehydrogenase (NADP) |
| ec:1.5.1.20 | methylenetetrahydrofolate reductase [NAD(P)H]; methylenetetrahydrofolate (reduced nicotinamide adenine dinucleotide phosphate) reductase; 5,10-methylenetetrahydrofolate reductase (NADPH); 5,10-methylenetetrahydrofolic acid reductase; 5,10-CH2-H4folate reductase; methylenetetrahydrofolate reductase (NADPH2); 5-methyltetrahydrofolate:NAD+ oxidoreductase; 5-methyltetrahydrofolate:NAD+ oxidoreductase; methylenetetrahydrofolate (reduced riboflavin adenine dinucleotide) reductase; 5,10-methylenetetrahydrofolate reductase; methylenetetrahydrofolate reductase; N5,10-methylenetetrahydrofolate reductase; 5,10-methylenetetrahydropteroylglutamate reductase; N5,N10-methylenetetrahydrofolate reductase; methylenetetrahydrofolic acid reductase; 5-methyltetrahydrofolate:(acceptor) oxidoreductase (incorrect); 5,10-methylenetetrahydrofolate reductase (FADH2); MetF; methylenetetrahydrofolate reductase (NADPH); 5-methyltetrahydrofolate:NADP+ oxidoreductase |
| ec:2.1.1.245 | 5-methyltetrahydrosarcinapterin:corrinoid/iron-sulfur protein Co-methyltransferase; cdhD (gene name); cdhE (gene name) |
| ec:2.1.1.258 | 5-methyltetrahydrofolate:corrinoid/iron-sulfur protein Co-methyltransferase; acsE (gene name) |
| ec:2.3.1.169 | CO-methylating acetyl-CoA synthase |
| ec:3.5.4.9 | methenyltetrahydrofolate cyclohydrolase; Citrovorum factor cyclodehydrase; cyclohydrolase; formyl-methenyl-methylenetetrahydrofolate synthetase (combined); 5,10-methenyltetrahydrofolate 5-hydrolase (decyclizing) |
| ec:6.3.4.3 | formate---tetrahydrofolate ligase; formyltetrahydrofolate synthetase; 10-formyltetrahydrofolate synthetase; tetrahydrofolic formylase; tetrahydrofolate formylase |
