# Supplementary material for: Methane-yielding microbial communities processing lactate-rich substrates: a piece of the anaerobic digestion puzzle
Source: Biotechnol Biofuels. 2018 Apr 21;11:116. doi: 10.1186/s13068-018-1106-z (PMC5910564; doi:10.1186/s13068-018-1106-z)
Supplement: Supplementary file 7 — Additional file 7. Stable carbon isotopic composition of substrates and fermentation gas. [file 13068_2018_1106_MOESM7_ESM.docx]

Additional file 7. Stable carbon isotopic composition of substrates and fermentation gas.

| **Carbon isotopic composition of gas samples** | | | | |
| --- | --- | --- | --- | --- |
| Week of cultivation | Day of sampling | δ^13^C_CO2_ (‰) | δ^13^C_CH4_ (‰) | α_(CO2-CH4)_ |
| 44 | 6 | 0.4 | -33.1 | 1.035 |
| 45 | 8 | 0.7 | -30.5 | 1.032 |
|  | 11 | 1.3 | -31.1 | 1.033 |
|  | 13 | 0.9 | -31.9 | 1.034 |
| 46 | 15 | 1.6 | -31.0 | 1.034 |
|  | 18 | 1.8 | -31.8 | 1.035 |
|  | 20 | 2.0 | -31.0 | 1.034 |
| 47 | 22 | 2.00 | -31.6 | 1.035 |
|  | 25 | 1.9 | -31.7 | 1.035 |
|  | 27 | 2.2 | -31.7 | 1.035 |
| 48 | 29 | 1.8 | -31.6 | 1.034 |
|  | 32 | 1.5 | -31.6 | 1.034 |
| 49 | 36 | 1.4 | -30.9 | 1.033 |
|  | 39 | 1.1 | -31.5 | 1.034 |
| 50 | 41 | 1.0 | -31.1 | 1.033 |
|  | 46 | 1.3 | -31.2 | 1.033 |
| Average | | 1.4 | -31.5 | 1.034 |
| Minimum | | 0.4 | -33.1 | 1.032 |
| Maximum | | 2.2 | -30.5 | 1.035 |
| **Carbon isotopic composition of substrates** | | | | |
| Substrate | | | δ^13^C (‰) | Amount |
| Yeast extract | | | -23.2 | 0.5 g/L |
| Sodium lactate | | | -23.0 | 7 g/L |
| Sodium butyrate | | | -44.9 | 1.3 g/L |
| Propionic acid | | | -31.1 | 0.99 g/L |
| Acetic acid | | | -42.3 | 1.05 g/L |
