# Supplementary material for: Methane-yielding microbial communities processing lactate-rich substrates: a piece of the anaerobic digestion puzzle
Source: Biotechnol Biofuels. 2018 Apr 21;11:116. doi: 10.1186/s13068-018-1106-z (PMC5910564; doi:10.1186/s13068-018-1106-z)
Supplement: Supplementary file 8 — Additional file 8. Performance of the M1B methane-yielding microbial community processing a lactate-rich artificial medium between 44th and 50th week of cultivation. [file 13068_2018_1106_MOESM8_ESM.docx]

| **M1B** | | | |
| --- | --- | --- | --- |
| 1. **Characteristics of the biogas** | | | |
| **Total Biogas production:**  L/working volume of the bioreactor/d | | 36.3 ± 3.3 | |
| **Composition of biogas [%]:** | |  | |
| methane | | 71.9 ± 0.4 | |
| carbon dioxide | | 28.0 ± 1.1 | |
| hydrogen | | 0.01 ± 0.01 | |
| hydrogen sulphide | | 0.01 ± 0.01 | |
| **Methane production:** | | | |
| L-CH_4_/working volume of the bioreactor/d | | 25.0 ± 2.0 | |
| L-CH_4_/g COD | | 3.4 ± 0.3 | |
| 1. **Characteristics of the substrate and effluent after the methanogenic process** | | | |
|  | **Substrate** | | **Effluent** |
| **COD (g O_2_/L)** | 12.5 ±1.8 | | 0.6 ± 0.3 |
| **concentration of sulphide** (mg/L) | <0.05 * | | 0.01 ± 0.0 |
| **pH** | 4.91 ± 0.12 | | 7.40 ± 0.1 |
| **Redox potential in the UASB** bioreactor (mV) ** |  | | (-310)–(-320) |

Additional file 8. Performance of the M1B methane-yielding microbial community processing a lactate-rich artificial medium between 44^th^ and 50^th^ week of cultivation.

* – the limit of quantification

** – according to hydrogen electrode
